# Practical Synthesis from Streptomycin and Regioselective Partial Deprotections of (−)-(1*R*,2*S*,3*R*,4*R*,5*S*,6*S*)-1,3-Di(deamino)-1,3-diazido-2,5,6-tri-*O*-benzylstreptamine

**DOI:** 10.1021/acs.joc.3c02922

**Published:** 2024-03-01

**Authors:** Niteshlal Kasdekar, Michael R. Spieker, David Crich

**Affiliations:** † Department of Pharmaceutical and Biomedical Sciences, University of Georgia, 250 West Green Street, Athens, Georgia 30602, United States; ‡ Department of Biochemistry and Molecular Biology, University of Georgia, 120 East Green Street, Athens, Georgia 30602, United States; § Department of Chemistry, University of Georgia, 302 East Campus Road, Athens, Georgia 30602, United States; ∥ Complex Carbohydrate Research Center, University of Georgia, 315 Riverbend Road, Athens, Georgia 30602, United States

## Abstract

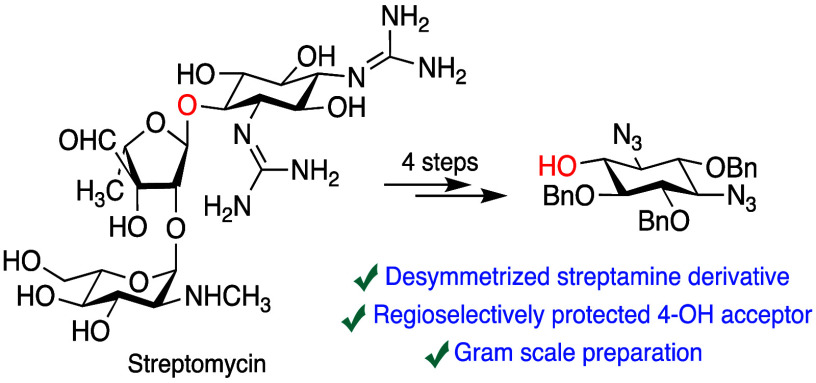

We describe the gram-scale synthesis of (−)-(1*R*,2*S*,3*R*,4*R*,5*S*,6*S*)-1,3-di(diamino)-1,3-diazido-2,5,6-tri-*O*-benzylstreptamine from streptomycin by (i) hydrolysis of the two streptomycin guanidine residues, (ii) reprotection of the amines as azides, (iii) protection of all alcohols as benzyl ethers, and (iv) glycosidic bond cleavage with HCl in methanol. Protocols for regioselective monodebenzylation and regioselective reduction of a single azide in the product are also described, providing four optically pure building blocks for exploitation in novel aminoglycoside synthesis.

The continued spread of multidrug resistant infectious diseases demands the equally continuous development of novel anti-infective agents with which to combat them.^1−4^ The aminoglycoside antibiotics (AGAs), with their well-understood mechanisms of action and resistance,^5−11^ excellent activity against Gram-negative pathogens,^7,12^ and wide commercial availability, are excellent starting materials for further development and as such have undergone something of a renaissance in recent years.^13,14^ These advantages are offset by nephrotoxic and ototoxic side effects^15,16^ such that next-generation AGA development programs target circumvention of resistance mechanisms concomitant with the reduction of these undesirable side effects.^17−20^

Although the first AGA to be isolated and developed, streptomycin **1**, is based on the streptamine core **2**, most common AGAs are constructed around 2-deoxystreptamine **3** and hence belong to the 2-deoxystreptamine class, which is subdivided into the 4,5-disubstituted and 4,6-disubstituted 2-deoxystreptamine AGAs, exemplified by neomycin B **4** and gentamicin C_1a_**5**, respectively. The hybrimycins, isolated from a mutant of *Streptomyces fradiae*,^21−26^ are 2-hydroxy analogs of the 4,5-AGAs that display parent-like levels of antibacterial activity and, at the target level, of inhibition of ribosomal protein synthesis,^21,23,24^ with hybrimycin A_1_**6** serving as an example of the class. In the 4,6-AGAs, the enzymatically derived 2-hydroxygentamicins **7** are reported to have comparable activity toward wild-type strains as the gentamicins **5** themselves^21,24^ and even greater activity than the gentamicins toward resistant strains.^27^ More recently, the 4,6-AGA, 2-hydroxyarbekacin **8**, has been reported to have excellent antibacterial activity against MRSA and *Pseudomonas aeruginosa*. Most importantly though in the context of the need for next generation AGAs with reduced toxicity it was reported that the 2-hydroxygentamicin **7** shows reduced toxicity in mice compared to the parent gentamicin,^27^ and that 2-hydroxyarbekacin **8** is less nephrotoxic than arbekacin **9** in vitro and in a rat model.^28^ Similarly, it is known streptomycin **1** itself displays low nephrotoxicity compared to other AGAs (Figure 1).^29,30^

**Figure 1:**
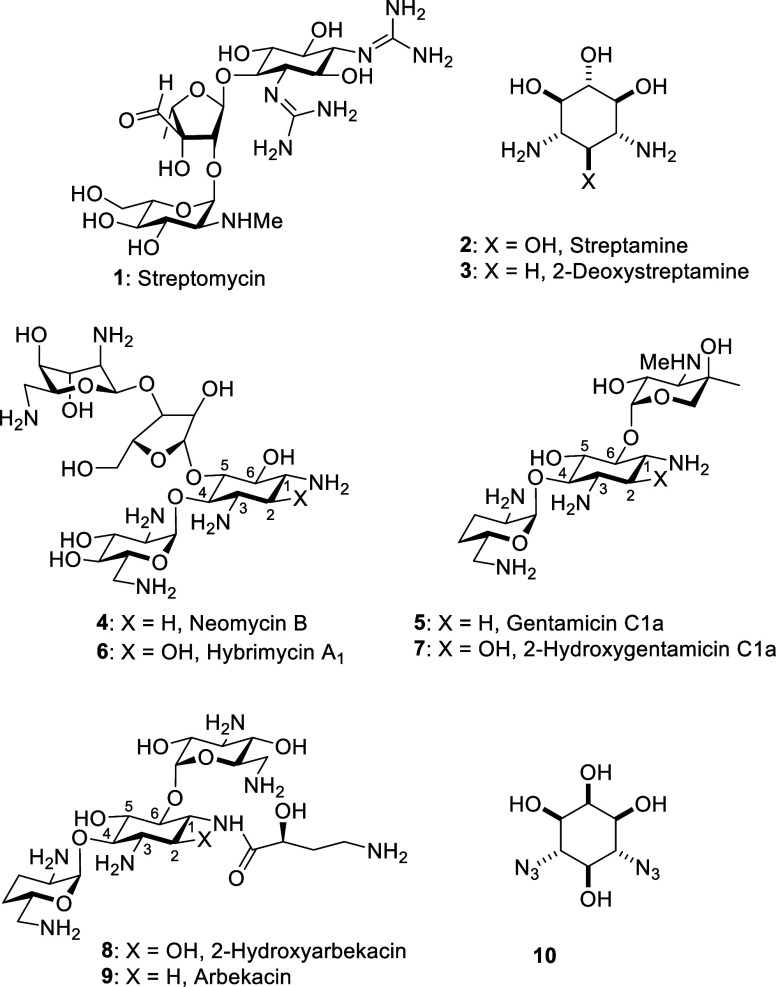
Streptamine and 2-deoxystreptamine and derived aminoglycoside antibiotics.

The retention of antibacterial activity by the 2-hydroxy variants of the AGAs coupled with the potential for reduced nephrotoxicity^28−30^ suggests a broader exploration of the 2-hydroxy AGAs, which in turn gives rise to the need for effective synthetic methods and building blocks. Streptamine **2** is commercial, or can be obtained by several straightforward literature methods from commercial inositol,^31−35^ but its use as starting material would require desymmetrization of this meso-compound in order to provide any enantiomerically pure AGA targets, and while progress has been made in desymmetrizing glycosylations of meso-diols, including inositol derivatives,^36−40^ these methods are not sufficient to enable the practical synthesis of meaningful quantities of compound.

Desymmetrizing glycosylation reactions of 2-deoxystreptamine derivatives themselves have been described by Nagorny and co-workers with catalysis by readily available chiral phosphoric acid catalysts but selectivities are not ideal, leading to the need for impractical chromatographic separations.^41^ Potentially, desymmetrization of the diazidostreptamine isomer **10**, available in four straightforward steps from myo-inositol,^31−34^ by ketal formation with d-camphor dimethyl acetal as described for *myo*-inositol^42^ followed by glycosylation could be of use, but ultimately we considered that the most practical way forward is exploitation of the desymmetrization of streptamine by nature in the form of streptomycin **1**. This approach is clearly related to the use of 2-hydroxygentamicin C1a **7** by Takahashi and co-workers as a substrate for the synthesis of 2-hydroxyarbekacin **8**, but as streptomycin **1** is widely commercially available on a large scale compared to the minor gentamicin component **8** clearly has broader potential. Accordingly, we describe here a straightforward gram-scale synthesis of a suitably regioselectively protected derivative of streptamine suitable for immediate use in glycosylation at the 4-position, as well as protocols for its regioselective deprotection giving ready access to several useful building blocks for use in medicinal chemistry campaigns.

Commercially available streptomycin sulfate **1** was reduced to dihydrostreptomycin with aqueous sodium borohydride as previously described.^43^ This was followed without purification by deguanylation in refluxing saturated aqueous barium hydroxide over 36 h^44^ and, again without purification by copper sulfate-catalyzed treatment with Stick’s reagent (imidazole-1-sulfonyl azide)^45−48^ in aqueous methanol for 16 h to give, after chromatographic purification, the anticipated 1,3-di(deamino)-1,3-diazidodihydrostreptomycin **11** and *N*′-methyl-1,3-di(deamino)-1,3-diazidodihydrostreptomycin derivative **12** in 52% and 14% yield, respectively, over the three steps. The isolation and characterization of **12** confirms the previously reported^49^ presence of *N*′-methylstreptomycin as an impurity in the commercial drug, a fact that was further supported by detection of an M+14 peak with formula C_22_H_41_N_7_O_12_ and 15% abundance relative to **1** in the ESIHRMS spectrum of the commercial material employed in this study. Compound **11** was then treated with benzyl bromide and sodium hydride in DMF at room temperature for 14 h to afford perbenzylated derivative **13** in 74% yield. Following earlier reports on the selective cleavage of the ribofuranosyl bond in streptomycin itself with either methanol or ethyl mercaptan in the presence of hydrogen chloride into streptidine and streptobiosaminide derivatives,^50,51^ compound **13** was heated to reflux with 3 N HCl in methanol for 16 h. After neutralization of the reaction mixture and removal of the volatiles the crude reaction was acetylated to facilitate chromatographic purification, which ultimately yielded the streptamine derivative **14** and the methyl streptobiosaminide **15** in 83% and 81% yields, respectively, the latter as essentially a single anomer whose configuration was established on the basis of NOE correlations and a ^3^*J*_H1,H2_ coupling constant of 4.1 Hz (Figure 2).^52^ Finally, the target desymmetrized streptamine mono-ol **16** was obtained in 92% yield on a gram scale by treatment of **14** with sodium methoxide (Scheme 1).

**Scheme 1:**
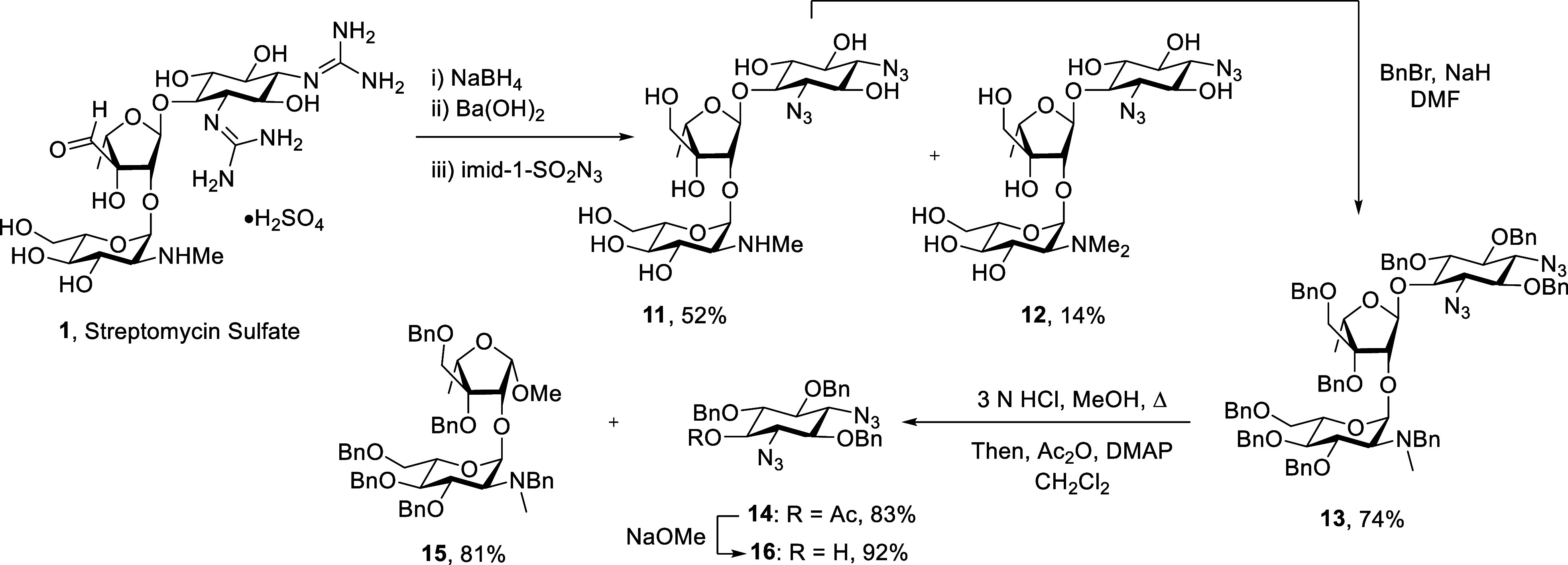
Synthesis of Streptamine Derivative **16** from Streptomycin **1**

**Figure 2:**
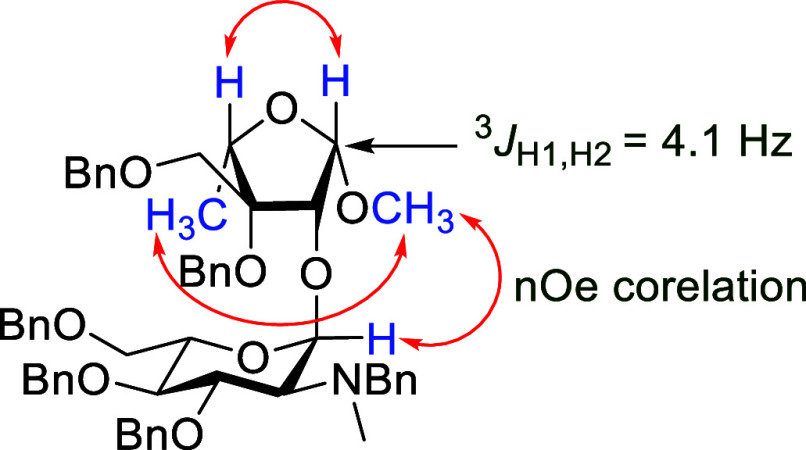
Diagnostic scalar coupling constant and NOE interactions for the assignment of the configuration of **15**.

With **16** available on gram scale, we turned to selective partial deprotection so as to provide a range of building blocks for use in the synthesis of novel AGAs and other systems. White light photolysis of **16** in acetonitrile in the presence of iodobenzene diacetate and iodine cleanly provided a diol **17** in 71% yield that was converted to the diacetate **18** in 90% yield to facilitate spectral interpretation (Scheme 2). The formation of **17** is the result of initial alkoxy radical generation followed by δ-hydrogen atom abstraction from the adjacent benzylic methylene group and eventually trapping by iodine and then hydrolysis;^53^ no evidence was found for the formation of a benzylidene acetal, presumably because it would span a *trans*-1,2-diol as opposed to the more usual *cis*-diol.^54^ Further selective functionalization of **17** is expected to follow the pattern established for related 1,2-diols.^20,55−58^ Reaction of **16** with boron trichloride in dichloromethane at −20 °C^59^ cleanly gave diols **19** and *meso*-**20** in 65 and 14% yield, respectively, with the regioselectivity an apparent function of proximity to the strongly electron-withdrawing azido groups. As with **17**, diols **19** and **20** were converted to their respective diacetates **21** and **22** for ease of structural elucidation (Scheme 2). Finally, treatment of **16** with stannous chloride and lithium iodide^60^ in ethyl acetate at 70 °C resulted in apparent proximity-induced regioselective reduction of a single of the two azido groups, whose location was identified following treatment of the crude reaction mixture with an excess of Boc_2_O and DMAP when the *N*-Boc oxazolidinone **23** was isolated in 67% yield (Scheme 2).

**Scheme 2:**
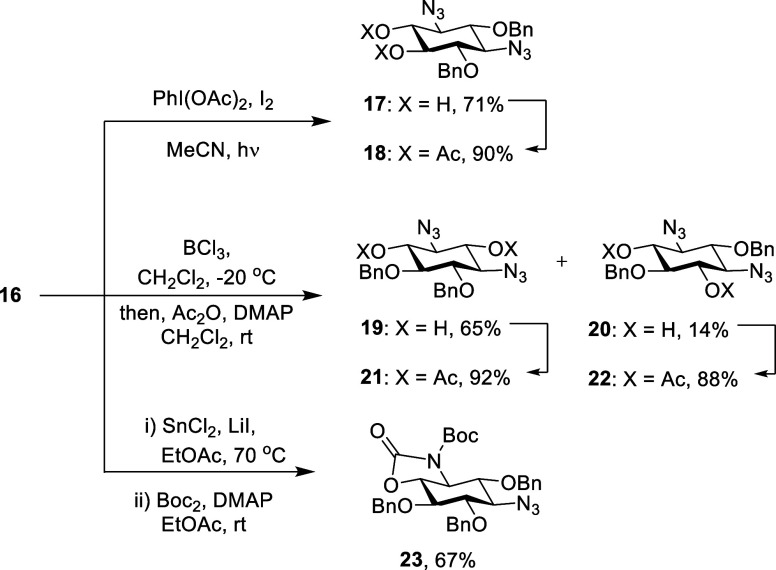
Regioselective Partial Deprotections of **16**

Overall, we have provided a convenient scalable route to four optically pure, regioselectively protected streptamine building blocks suitable for use in next-generation AGA syntheses from readily available streptomycin in a minimum of steps.

## Experimental Section

### General Experimental Methods

All reagents were purchased from commercial sources and used without further purification. Reaction mixtures were heated with the aid of an appropriately sized thermostatically controlled aluminum heating block. Thin-layer chromatography was carried out with 250 μm glass-backed silica plates, and the spots were revealed by UV absorption (254 nm) and by charring with a 20:80 v/v solution of sulfuric acid in ethanol or with a ceric ammonium molybdate solution. All organic solutions were concentrated under a vacuum at 30–45 °C on a rotary evaporator. Purification of crude residues was performed manually as well as by flash column chromatography. Specific rotations were recorded on an automatic polarimeter in CHCl_3_, MeOH at 589 nm and 23 ± 1 °C with a path length of 10 cm. Nuclear magnetic resonance (NMR) spectra of all compounds were obtained in CDCl_3_, CD_3_OD, or C_6_D_6_ at 500, 600, or 900 MHz, with chemical shifts (δ) calculated with respect to the residual solvent peak and given in ppm. Multiplicities are abbreviated as follows: s (singlet), d (doublet), t (triplet), q (quartet), dd (doublet of doublet), bs (broad singlet), and m (multiplet). Peak assignments were based on two-dimensional NMR (COSY, HSQC, and HMBC) experiments, and the configurational assignments were determined with the aid of selective 1D NOESY NMR experiments. High-resolution electrospray ionization (ESI) mass spectrometry spectra were recorded using a Thermo Scientific Orbitrap mass analyzer.

#### 1,3-Di(deamino)-1,3-diazidodihydrostreptomycin (**11**) and *N*′-Methyl-1,3-di(deamino)-1,3-diazidodihydrostreptomycin (**12**)

Streptomycin sulfate (10.0 g, 14.7 mmol, purchased from Sigma-Aldrich, CAS No. 3810-74-0) was dissolved in deionized water (70 mL) with stirring, and the pH was adjusted to 8.0 using triethylamine (∼0.6 mL). Sodium borohydride (0.34 g, 8.8 mmol) dissolved in deionized water (10 mL) was added dropwise to the stirred reaction mixture over a period of 5 min at room temperature (a slight elevation in reaction temperature was observed), which was then stirred at room temperature for 0.5 h before it was acidified to pH 1.5 by slow addition of 6 N H_2_SO_4_ (∼0.6 mL). After standing at room temperature for 10 min, the reaction mixture was added with stirring to methanol (350 mL) resulting in a white precipitate that was collected by vacuum filtration, washed with methanol (100 mL × 2), and dried under vacuum for 3 h to give crude dihydrostreptomycin sulfate (10.7 g) as confirmed by ESI mass spectrometry. This crude product (10.7 g, 14.7 mmol) was dissolved in deionized water (40 mL), treated with saturated aqueous Ba(OH)_2_ (250 mL), and stirred for 10 min at room temperature. The white precipitate of barium sulfate was filtered off, and the filtrate was heated to 125 °C for 36 h, after which it was cooled to room temperature, and the excess barium hydroxide was neutralized by addition of carbon dioxide (dry ice). The precipitates were filtered off and washed with water (100 mL), and the combined filtrate and washings were concentrated under reduced pressure at 60 °C to obtain the crude di(deguanidinyl)dihydrostreptomycin carbonate as a thick syrup (6.98 g) as confirmed by ESI mass spectrometry. This crude product (6.98 g, 14.0 mmol) was taken up in deionized water (80 mL), treated with NaHCO_3_ (11.7 g, 139.7 mmol) and CuSO_4_·5H_2_O (0.35 g, 1.40 mmol), and cooled to 0 °C in an ice bath before imidazole-1-sulfonyl azide hydrochloride (7.32 g, 34.9 mmol) was added portionwise with stirring over 10 min. The reaction mixture was held at 0 °C for 0.5 h, then allowed to come to room temperature, and stirred for 16 h. After completion, the reaction mixture was recooled to 0 °C in an ice bath, butylamine (0.5 mL) was added dropwise, and stirring was continued for 0.5 h before the solvents were evaporated under reduced pressure (below 40 °C) to give a crude mixture that was purified by silica gel column chromatography eluting with ammonical methanol in dichloromethane (gradients 25%, 30%, 35%, and 40%) to obtain compound **11** (4.54 g, 52% overall) and **12** (1.26 g, 14% overall).

##### Compound **11**:

*R*_*f*_ = 0.3 in 40% ammonical MeOH in CH_2_Cl_2_; [α]_*D*_^21^ = −77.0 (*c* = 2.5, MeOH); ^1^H NMR (900 MHz, CD_3_OD) δ 5.56 (d, *J* = 3.5 Hz, 1H, H1″), 5.40 (d, *J* = 1.8 Hz, 1H, H1′), 4.37 (d, *J* = 1.8 Hz, 1H, H2′), 4.25 (q, *J* = 6.4 Hz, 1H, H4′), 3.90–3.85 (m, 2H, H3″ and H6a″), 3.81 (dd, *J* = 12.0, 4.7 Hz, 1H, H6b″), 3.76 (ddd, *J* = 10.4, 4.7, 2.3 Hz, 1H, H4″), 3.64–3.56 (m, 2H, H6′), 3.48 (t, *J* = 9.4 Hz, 1H, H5″), 3.43 (t, *J* = 10.0 Hz, 1H, H4), 3.39 (t, *J* = 9.3 Hz, 1H, H2), 3.32–3.29 (m, 2H, H5 and H6), 3.25 (t, *J* = 9.9 Hz, 1H, H3), 3.22–3.18 (t, *J* = 9.9 Hz, 1H, H1), 3.18–3.16 (m, 1H, H2″), 2.88 (s, 3H, H5′), 1.22 (d, *J* = 6.4 Hz, 3H, H5′); ^13^C{^1^H} NMR (226 MHz, CD_3_OD) δ 107.4 (C1′), 95.4 (C1″), 86.0 (C2′), 82.1(C3′), 79.2 (C6), 79.0 (C4′), 74.9 (C4″), 74.7 (C2), 74.0 (C5), 73.8 (C1), 71.3 (C3″), 71.1 (C5″), 69.5 (C3), 68.7 (C4), 65.5 (C6′), 63.4 (C2″), 62.0 (C6′), 33.0 (NCH_3_), 13.9 (C5′); HRMS (ESI-TOF) *m*/*z*: [M + H]^+^ calculated for C_19_H_34_O_12_N_7_ 552.2260; found 552.2240.

##### Compound **12**:

*R*_*f*_ = 0.4 in 40% ammonical MeOH in CH_2_Cl_2_; [α]_D_^21^ = −88.4 (*c* = 1.0, MeOH); ^1^H NMR (900 MHz, CD_3_OD) δ 5.59 (d, *J* = 3.3 Hz, 1H, H1″), 5.37 (d, *J* = 1.9 Hz, 1H, H1′), 4.36 (d, *J* = 1.9 Hz, 1H, H2′), 4.26 (q, *J* = 6.3 Hz, 1H, H4′), 4.05 (ddd, *J* = 10.6, 8.5, 1H, H3″), 3.87 (dd, *J* = 11.7, 1.9 Hz, 1H, H6a″), 3.79 (dd, *J* = 11.9, 4.8 Hz, 1H, H6b″), 3.76 (ddd, *J* = 10.0, 4.4, 2.0 Hz, 1H, H5″), 3.63–3.57 (m, 2H, H6′), 3.49 (t, *J* = 9.2 Hz, 1H, H4″), 3.42 (t, *J* = 9.9 Hz, 1H, H2), 3.38 (t, *J* = 9.2 Hz, 1H, H5), 3.35–3.32 (m, 2H, H2″ and H6), 3.28 (t, *J* = 9.7 Hz, 1H, H4), 3.24 (t, *J* = 9.9 Hz, 1H, H3), 3.19 (t, *J* = 9.6 Hz, 1H, H1), 3.06 (s, 6H, N(CH_3_)_2_), 2.62 (d, *J* = 1.4 Hz, 1H), 1.20 (d, *J* = 6.3 Hz, 3H, H5′); ^13^C{^1^H} NMR (226 MHz, CD_3_OD) δ 107.4 (C1′), 95.9 (C1″), 84.9 (C2″), 81.9 (C3′), 79.3 (C4′), 79.1 (C4), 74.8 (C5″), 74.4 (C5), 74.0 (C6), 73.9 (C1), 72.1 (C4″), 69.5 (C3), 69.1 (C3″), 68.7 (C2), 67.6 (C2′), 65.5 (C6′), 62.1 (C6″), 42.1 (N(CH_3_)_2_, 14.0 (C5′); HRMS (ESI-TOF) *m*/*z*: [M + H]^+^: calculated for C_20_H_36_O_12_N_7_ 566.2416; found 566.2416.

#### 1,3-Di(deamino)-1,3-diazido-2,5,6-tri-*O*-benzyl-4-*O*-(3′,6′-di-*O*-benzyl-2′-*O*-(2″-*N*-benzyl-3″,4″,6″-tri-*O*-benzyldihydrostreptomycin (**13**)

Sodium hydride (4.96 g, 124.0 mmol, 60% in mineral oil) was added in two portions to an ice-cold stirred solution of **11** (4.56 g, 8.27 mmol) in DMF (120 mL) under an Ar atmosphere, followed after 10 min by dropwise addition of benzyl bromide (11.9 mL, 99.2 mmol) over 10 min. The reaction mixture was allowed to come to room temperature and was stirred for 16 h, after which it was quenched by dropwise addition of methanol (5 mL), diluted with ethyl acetate (200 mL), and washed with ice-cold water (200 mL × 2) and brine (200 mL), and the organic layer was dried over Na_2_SO_4_ and concentrated under reduced pressure. The crude reaction mixture was purified by silica gel column chromatography eluting with ethyl acetate in hexane (gradients 5%, 10%, and 15%) to obtain **13** (8.32 g, 74%): *R*_*f*_ = 0.35 in 15% EtOAc in hexane; [α]_D_^21^ = −31.5 (*c* = 1.2, CHCl_3_); ^1^H NMR (500 MHz, CDCl_3_) δ 7.47–7.17 (m, 45H, Ar), 5.76 (d, *J* = 3.5 Hz, 1H, H1′), 5.14 (d, *J* = 3.3 Hz, 1H, H1″), 4.97–4.71 (m, 12H, 6 × CH_2_Ph), 4.59–4.34 (m, 4H, 2 × CH_2_Ph), 4.54 (d, *J* = 3.5 Hz, 1H, H2′), 4.31 (q, *J* = 6.4 Hz, 1H, H4′), 4.09 (dd, *J* = 10.9, 8.5 Hz, 1H, H3″), 3.89 (dt, *J* = 10.1, 2.6 Hz, 1H, H5″), 3.82 (s, 2H, NCH_2_Ph), 3.76 (dd, *J* = 10.1, 8.5 Hz, 1H, H4″), 3.70–3.61 (m, 3H, H6″ and H4), 3.54 (d, *J* = 9.9 Hz, 1H, H6a′), 3.43–3.35 (m, 4H, H6b′ and H2, H5, H6), 3.27 (t, *J* = 9.7 Hz, 1H, H3), 3.1 5 (t, *J* = 9.9 H z, 1H, H1), 2.94 (dd, *J* = 10.9, 3.4 Hz, 1H, H2″), 2.39 (s, 3H, NCH_3_), 1.13 (d, *J* = 6.5 Hz, 3H, H5′); ^13^C{^1^H} NMR (126 MHz, CDCl_3_) δ 140.6, 139.4, 139.1, 138.6, 138.5, 138.0, 138.0, 137.7, 137.2, 128.8, 128.6, 128.6, 128.5, 128.5, 128.4, 128.4, 128.3, 128.3, 128.2, 128.2, 128.1, 128.0, 127.9, 127.8, 127.7, 127.6, 127.5, 127.5, 127.5, 127.3, 127.1, 126.8, 126.7 (aromatic), 105.9 (C1′), 101.5 (C1″), 84.6 (C3′), 84.0 (C2′), 82.4 (C2), 81.0 (C3), 80.4 (C1), 79.8(C4″), 79.0 (C3″), 78.2 (C4′), 76.0 (C4), 75.9, 75.5, 75.4, 74.5, 73.7, 73.7, 73.4 (8 × CH_2_Ph), 71.3 (C5″), 69.2 (C6′), 68.6 (C6″), 67.3 (C5), 67.3 (C6), 67.1 (CH_2_Ph), 65.3 (C2″), 62.0 (NCH_2_Ph), 38.6 (NCH_3_), 13.3 (C5′); HRMS (ESI-TOF) *m*/*z*: [M + H]^+^ calculated for C_82_H_88_O_12_N_7_ 1362.6391; found 1362.6390.

#### 4-*O*-Acetyl-1,3-di(deamino)-1,3-diazido-2,5,6-tri-*O*-benzylstreptamine (**14**) and Methyl 3′,6′-Di-*O*-benzyl-2-*O*-(2″-*N*-benzyl-3″,4″,6″-tri-*O*-benzyl)-β-streptobiosaminide (**15**)

Compound **13** (4.0 g, 2.45 mmol) was suspended in 3 N HCl in methanol (40 mL), and dichloromethane (4.0 mL) was added until a clear solution was obtained. The reaction mixture was then heated to reflux with stirring for 16 h before it was cooled in an ice bath, and triethylamine (5 mL) was added dropwise. The solvents were evaporated under reduced pressure below 45 °C, and the resulting thick syrup was taken up in ethyl acetate (80 mL), washed with saturated aqueous NaHCO_3_ (80 mL × 2) and brine (80 mL), dried over Na_2_SO_4_, and concentrated under reduced pressure. The residue was purified by silica gel column chromatography eluting with ethyl acetate in hexane (gradients 5%, 10%, 15%, and 20%) to obtain an inseparable mixture of **15** and **16** (4.35 g), which was dissolved in CH_2_Cl_2_ (20 mL), treated with acetic anhydride (0.6 mL, 5.90 mmol) and DMAP (0.25 g, 2.1 mmol), and stirred at room temperature for 0.5 h. After completion, the solvents were removed under reduced pressure and the residue was purified by silica gel column chromatography eluting with ethyl acetate in hexane (gradient 5%, 10%, 15% and 20%) to give **14** (1.33 g, 83%) and **15** (2.12 g, 81%).

##### Compound **14**:

*R*_*f*_ = 0.55 in 20% EtOAc in hexane; [α]_D_^21^ = −4.1 (*c* = 1.0, CHCl_3_); ^1^H NMR (900 MHz, CDCl_3_) δ 7.43–7.23 (m, 15H, Ar), 4.92 (t, *J* = 10.2 Hz, 1H, H4), 4.86–4.79 (m, 5H, CH_2_Ph), 4.63 (d, *J* = 11.4 Hz, 1H, CH_2_Ph), 3.52 (t, *J* = 9.6 Hz, 1H, H5), 3.49 (t, *J* = 10.3 Hz, 1H, H1), 3.46 (t, *J* = 10.3 Hz, 1H, H3), 3.37 (t, *J* = 9.7 Hz, 1H, H2), 3.21 (t, *J* = 9.9 Hz, 1H, H6), 1.99 (s, 3H, CH_3_); ^13^C{^1^H} NMR (226 MHz, CDCl_3_) δ 169.9 (C=O), 137.9, 137.5, 137.1, 128.8, 128.7, 128.7, 128.7, 128.5, 128.5, 128.3, 128.1, 127.8 (aromatic), 81.3 (C5), 80.9 (C2), 79.4 (C6), 76.3 (CH_2_Ph), 76.2 (CH_2_Ph), 75.8 (CH_2_Ph), 71.8 (C4), 67.4 (C1), 65.3 (C3), 20.9 (CH_3_); HRMS (ESI-TOF) *m*/*z*: [M + Na]^+^ calculated for C_29_H_30_O_5_N_6_Na 565.2169; found 565.2156.

##### Compound **15**:

*R*_*f*_ = 0.45 in 20% EtOAc in hexane; [α]_D_^21^ = −41.8 (*c* = 1.0, CHCl_3_); ^1^H NMR (500 MHz, CDCl_3_) δ 7.40–7.11 (m, 30H, aromatic), 5.11 (d, *J* = 4. One Hz, 1H, H1′), 5.06 (d, *J* = 3.3 Hz, 1H, H1″), 4.96 (ABq, *J* = 11.2 Hz, 2H, CH_2_Ph), 4.85–4.63 (m, 4H, 2 × CH_2_Ph), 4.58–4.44 (m, 4H, 2 × CH_2_Ph), 4.42 (d, *J* = 4.1 Hz, 1H, H2′), 4.38 (q, *J* = 6.4 Hz, 1H, H4′), 4.05 (dd, *J* = 10.9, 8.3 Hz, 1H, H3″), 3.90–3.75 (m, 5H, NCH_2_Ph, H4″, H5″ and H6a″), 3.65 (dd, *J* = 10.5, 1.9 Hz, 1H, H6b″), 3.58 (d, *J* = 9.9 Hz, 1H, H6a′), 3.48 (d, *J* = 9.9 Hz, 1H, H6b′), 3.40 (s, 3H, OCH_3_), 2.99 (dd, *J* = 10.9, 3.3 Hz, 1H, H2″), 2.40 (s, 3H, NCH_3_), 1.31 (d, *J* = 6.5 Hz, 3H, H5′); ^13^C{^1^H} NMR (126 MHz, CDCl_3_) δ 140.6, 139.4, 139.0, 138.4, 138.0, 137.9, 128.5, 128.5, 128.4, 128.4, 128.3, 128.2, 128.1, 127.9, 127.8, 127.8, 127.8, 127.7, 127.6, 127.2, 126.9, 126.8 (aromatic), 107.2 (C1′), 102.2 (C1″), 85.8 (C3′), 83.9 (C2′), 80.3 (C4″), 78.9 (C3″), 77.6 (C4′), 74.8 (C5″), 73.7, 73.7, 73.6, 71.3 (4 × CH_2_Ph), 69.1 (C6′), 68.5 (C6″), 67.6 (CH_2_Ph), 65.8 (C2″), 61.1 (CH_2_Ph), 56.0 (OCH_3_), 38.9 (NCH_3_), 13.4 (C5′); HRMS (ESI-TOF) *m*/*z*: [M + H]^+^ calculated for C_56_H_64_O_9_N 894.4575; found 894.4568.

#### 1,3-Di(deamino)-1,3-diazido-2,5,6-tri-*O*-benzylstreptamine (**16**)

Sodium methoxide (0.26 g, 4.90 mmol) was added to a stirred solution of **14** (1.33 g, 2.45 mmol) in anhydrous CH_2_Cl_2_:MeOH (1:1 v/v, 16 mL) at room temperature and stirring continued for 6 h before the reaction mixture was neutralized with Amberlite IRC120 H^+^ resin and filtered through a cotton wool plug and the filtrate concentrated under reduced pressure. The residue was subjected to silica gel column chromatography eluting with ethyl acetate in hexane (gradients 10%, 20%) to give **16** (1.12 g, 92%): *R*_*f*_ = 0.45 in 20% EtOAc in hexane; [α]_D_^21^ = −4.4 (*c* = 1.0, CHCl_3_); ^1^H NMR (900 MHz, CDCl_3_) δ 7.39–7.24 (m, 15H, Ar), 4.85 (d, *J* = 11.2 Hz, 1H, CH_2_Ph), 4.81–4.74 (m, 4H, 2 × CH_2_Ph), 4.68 (d, *J* = 11.2 Hz, 1H, CH_2_Ph), 3.42 (t, *J* = 10.0 Hz, 1H, H1), 3.33 (t, *J* = 9.9. Hz, 1H, H5) 3.35–3.28 (m, 2H, H3, H4), 3.23 (t, *J* = 9.6 Hz, 1H, H2), 3.08 (t, *J* = 9.5 Hz, 1H, H6); ^13^C{^1^H} NMR (226 MHz, CDCl_3_) δ 138.0, 137.5, 137.1, 128.7, 128.6, 128.6, 128.6, 128.6, 128.3, 128.3, 128.3, 128.3, 128.2, 128.1, 128.0, 127.9 (aromatic), 82.9 (C5), 80.8 (C2), 79.5 (C6), 75.9 (CH_2_Ph), 75.9 (CH_2_Ph), 75.7 (CH_2_Ph), 72.7 (C4), 67.6 (C1), 66.8 (C3); HRMS (ESI-TOF) *m*/*z*: [M + Na]^+^ calculated for C_27_H_28_O_4_N_6_Na 523.2064; found 523.2062.

#### 1,3-Di(deamino)-1,3-diazido-2,6-di-*O*-benzylstreptamine (**17**)

A solution of **16** (24 mg, 0.05 mmol) and (diacetoxyiodo)benzene (23 mg, 0.07 mmol) in anhydrous acetonitrile (0.8 mL) was stirred with shielding from ambient light for 0.5 h before iodine (7 mg, 0.03 mmol) was added, and the reaction mixture was irradiated with white light (300 W, tungsten lamp) for 2 h. The reaction mixture was cooled to room temperature, diluted with EtOAc, and washed with saturated aqueous Na_2_S_2_O_3_. The aqueous layer was extracted with EtOAc, and the combined organic layer was washed with brine (20 mL), dried over Na_2_SO_4_, and concentrated under reduced pressure. The crude mixture was purified by silica gel column chromatography eluting with ethyl acetate in hexane (gradient 20%, 30%, and 40%) to afford diol **17** (13.7 mg, 71%) as a colorless thick syrup: *R*_*f*_ = 0.35 in 40% EtOAc in hexane; [α]_D_^21^ = −49.6 (*c* = 1.0, CHCl_3_); ^1^H NMR (500 MHz, benzene-*d*_6_) δ 7.44 (d, *J* = 7.6 Hz, 2H, Ar), 7.37 (d, *J* = 7.5 Hz, 2H, Ar), 7.23–7.18 (m, 4H, Ar), 7.14–7.10 (m, 2H, Ar), 4.77 (d, *J* = 11.3 Hz, 1H, CH_2_Ph), 4.69 (d, *J* = 12.4 Hz, 3H, CH_2_Ph), 3.06 (t, *J* = 9.3 Hz, 1H, H5), 3.01 (t, *J* = 9.9 Hz, 1H, H1), 2.86 (t, *J* = 9.8 Hz, 1H, H3), 2.79 (td, *J* = 9.6, 5.1 Hz, 2H, H4 and H6), 2.71 (t, *J* = 9.7 Hz, 1H, H2), 2.29 (s, 1H, OH), 2.23 (s, 1H, OH); ^13^C{^1^H} NMR (126 MHz, benzene-*d*_6_) δ 138.7, 138.1, 128.8, 128.7, 128.7, 128.4, 128.4, 128.2, 128.0 (aromatic), 80.1 (C2), 79.5 (C6), 75.6 (C5), 75.3 (CH_2_Ph), 75.2 (CH_2_Ph), 72.8 (C4), 67.4 (C1), 67.1 (C3); HRMS (ESI-TOF) *m*/*z*: [M + Na]^+^ calculated for C_20_H_22_N_6_O_4_Na 433.1600; found 433.1586.

#### 4,5-Di-*O*-acetyl-1,3-di(deamino)-1,3-diazido-2,6-di-*O*-benzylstreptamine (**18**)

Diol **17** (13.7 mg) was treated with Ac_2_O (20 μL, 0.201 mmol) and DMAP (5 mg, 0.040 mmol) in CH_2_Cl_2_ (1.0 mL) for 0.5 h at room temperature before it was quenched with MeOH (50 μL). The solvents were removed under reduced pressure, and the residue was purified by silica gel column chromatography to obtain diester **18** (15 mg, 90%) as a colorless oil: *R*_*f*_ = 0.40 in 20% EtOAc in hexane; [α]_D_^21^ = −52.4 (*c* = 1.0, CHCl_3_); ^1^H NMR (500 MHz, benzene-*d*_6_) δ 7.40 (d, *J* = 7.5 Hz, 2H, Ar), 7.30 (d, *J* = 7.5 Hz, 2H, Ar), 7.25–7.17 (m, 4H, Ar), 7.13–7.09 (m, 2H, Ar), 5.08 (t, *J* = 9.7 Hz, 1H, H5), 4.86 (t, *J* = 10.3 Hz, 1H, H4), 4.68–4.58 (m, 3H, CH_2_Ph), 4.45 (d, *J* = 11.4 Hz, 1H, CH_2_Ph), 2.95–2.88 (m, 2H, H1 and H3), 2.85 (t, *J* = 9.9 Hz, 1H, H6), 2.65 (t, *J* = 9.8 Hz, 1H, H2), 1.74 (s, 3H, CH_3_), 1.62 (s, 3H, CH_3_); ^13^C{^1^H} NMR (126 MHz, benzene-*d*_6_) δ 169.3 (C=O), 169.2 (C=O), 138.2, 137.9, 128.7, 128.7, 128.6, 128.4, 128.3, 128.2, 128.0, 128.0 (aromatic), 78.9 (C6), 78.7 (C2), 75.7 (CH_2_Ph), 75.5 (CH_2_Ph), 73.1 (C5), 70.8 (C4), 67.0 (C1), 64.8 (C3), 20.2 (CH_3_), 20.2 (CH_3_); HRMS (ESI-TOF) *m*/*z*: [M + Na]^+^ calculated for C_24_H_26_N_6_O_6_Na 517.1806; found 517.1801.

#### 1,3-Di(deamino)-1,3-diazido-5,6-di-*O*-benzylstreptamine (**19**) and 1,3-Di(deamino)-1,3-diazido-2,5-di-*O*-benzylstreptamine (**20**)

BCl_3_ (220 μL, 1 M in CH_2_Cl_2_, 0.22 mmol) was added to a stirred solution of **16** (50 mg, 0.10 mmol) in anhydrous CH_2_Cl_2_ (2.0 mL) cooled to −20 °C. After stirring for 2 h, the reaction was quenched by addition of MeOH (100 μL), the reaction mixture was diluted with CH_2_Cl_2_ and washed with saturated aqueous NaHCO_3_, and the aqueous layer was extracted with CH_2_Cl_2_. The combined organic layer was washed with brine, dried over Na_2_SO_4_, and concentrated under reduced pressure to give a residue that was purified by silica gel column chromatography eluting with ethyl acetate in hexane (gradients 10%, 20%, and 30%) to obtain **19** (27 mg, 65%) and **20** (6 mg, 14%) both as colorless thick syrups.

##### **19**:

*R*_*f*_ = 0.30 in 30% EtOAc in hexane; [α]_D_^21^ = −58.8 (*c* = 1.0, CHCl_3_); ^1^H NMR (500 MHz, CDCl_3_) δ 7.42–7.28 (m, 10H, Ar), 4.93 (d, *J* = 11.3 Hz, 1H, PhCH_2_), 4.86 (s, 2H, PhCH_2_), 4.76 (d, *J* = 11.3 Hz, 1H, PhCH_2_), 3.48–3.39 (m, 3H, H5, H4 and H1), 3.34 (td, *J* = 9.2, 3.6 Hz, 2H, H2 and H6), 3.25 (t, *J* = 9.8, Hz, 1H, H3), 2.69 (s, 1H, OH), 2.53 (s, 1H, OH); ^13^C{^1^H} NMR (126 MHz, CDCl_3_) δ 138.0, 137.5, 128.9, 128.7, 128.4, 128.3, 128.3, 128.1 (aromatic), 83.4 (C6), 80.9 (C5), 76.0 (CH_2_Ph), 75.9 (CH_2_Ph), 73.1 (C4), 72.0 (C2), 67.5 (C3), 66.6 (C1); HRMS (ESI-TOF) *m*/*z*: [M + Na]^+^ calculated for C_20_H_22_N_6_O_4_Na 433.1600; found 433.1586.

##### **20**:

*R*_*f*_ = 0.35 in 30% EtOAc in hexane; ^1^H NMR (600 MHz, C_6_D_6_) δ 7.45–7.41 (m, 2H, Ar), 7.29–7.16 (m, 6H, Ar), 7.15–7.09 (m, 2H, Ar), 4.69 (s, 2H, CH_2_Ph), 4.65 (s, 2H, CH_2_Ph), 2.96–2.90 (m, 2H, H4 and H6), 2.88–2.79 (m, 3H, H1, H3, H5), 2.69 (t, *J* = 9.7 Hz, 1H, H2), 1.90 (s, 2H, 2 × OH); ^13^C{^1^H} NMR (151 MHz, C_6_D_6_) δ 138.9, 138.1, 128.8, 128.7, 128.6, 128.6, 128.3, 128.1, 128.1, 128.0 (aromatic), 82.0 (C5), 79.2 (C2), 75.5 (CH_2_Ph), 74.9 (CH_2_Ph), 73.1 (C4), 73.1 (C6), 67.5 (C1), 67.5(C3); HRMS (ESI-TOF) *m*/*z*: [M + Na]^+^ calculated for C_20_H_22_N_6_O_4_Na 433.1600; found 433.1588.

#### 2,4-Di-*O*-acetyl-1,3-di(deamino)-1,3-diazido-5,6-di-*O*-benzylstreptamine (**21**)

The diol **19** (26 mg, 0.06 mmol) was treated with Ac_2_O (20 μL, 0.20 mmol) and DMAP (5 mg, 0.04 mmol) in anhydrous CH_2_Cl_2_ (1.0 mL) and stirred for 0.5 h at room temperature before concentration under reduced pressure to give a residue that was subjected to silica gel column chromatography eluting with ethyl acetate in hexane (gradients 10% and 20%) to obtain **21** (24.8 mg, 92%) as a colorless oil: *R*_*f*_ = 0.40 in 20% EtOAc in hexane; [α]_D_^21^ = −50.6 (*c* = 1.0, CHCl_3_); ^1^H NMR (500 MHz, benzene-*d*_6_) δ 7.35–7.30 (m, 2H, Ar), 7.24 (d, *J* = 7.5 Hz, 2H, Ar), 7.20–7.16 (m, 4H, Ar), 7.13–7.06 (m, 2H, Ar), 5.06 (t, *J* = 10.2 Hz, 1H, H4), 4.88 (t, *J* = 10.4 Hz, 1H, H2), 4.67 (d, *J* = 11.6 Hz, 1H, CH_2_Ph), 4.62–4.53 (m, 2H, CH_2_Ph), 4.50 (d, *J* = 11.6 Hz, 1H, CH_2_Ph), 3.13 (t, *J* = 9.6 Hz, 1H, H5), 2.95 (t, *J* = 10.2 Hz, 1H, H1), 2.85 (t, *J* = 10.5 Hz, 1H, H3), 2.78 (t, *J* = 9.7 Hz, 1H, H6), 1.76 (s, 3H, CH_3_), 1.65 (s, 3H, CH_3_); ^13^C{^1^H} NMR (126 MHz, benzene-*d*_6_) δ 168.8 (C=O), 168.7 (C=O), 138.5, 138.3, 128.7, 128.7, 128.4, 128.4, 128.2, 128.0, 127.9, 127.7 (aromatic), 81.2 (C5), 80.3 (C6), 76.0 (CH_2_Ph), 75.3 (CH_2_Ph), 71.4 (C4), 70.4 (C2), 65.2 (C1), 63.3 (C3), 20.3 (CH_3_), 20.2 (CH_3_); HRMS (ESI-TOF) *m*/*z*: [M + Na]^+^ calculated for C_24_H_26_N_6_O_6_Na 517.1806; found 517.1809.

#### 4,6-Di-*O*-acetyl-1,3-di(deamino)-1,3-diazido-2,5-di-*O*-benzylstreptamine (**22**)

The diol **20** (6.5 mg, 0.015 mmol) was treated with Ac_2_O (20 μL, 0.20 mmol) and DMAP (5 mg, 0.04 mmol) in anhydrous CH_2_Cl_2_ (1.0 mL) and stirred for 0.5 h at room temperature before concentration under reduced pressure to give a residue that was subjected to silica gel column chromatography eluting with ethyl acetate in hexane (gradients 10% and 20%) to obtain **22** (7 mg, 88%) as a colorless oil: *R*_*f*_ = 0.35 in 20% EtOAc in hexane; ^1^H NMR (500 MHz, benzene-*d*_6_) δ 7.39 (d, *J* = 7.6 Hz, 2H, Ar), 7.25–7.17 (m, 6H, Ar), 7.15–7.03 (m, 2H, Ar), 5.02 (t, *J* = 10.2 Hz, 2H, H2 and H4), 4.55 (s, 2H, CH_2_Ph), 4.45 (s, 2H, CH_2_Ph), 3.15 (t, *J* = 9.8 Hz, 1H, H5), 2.89 (t, *J* = 10.2 Hz, 2H, H1 and H3), 2.54 (t, *J* = 9.9 Hz, 1H, H6), 1.68 (s, 6H, 2 × CH_3_); ^13^C{^1^H} NMR (126 MHz, benzene-*d*_6_) δ 168.7 (2 × C=O), 138.3, 137.9, 128.7, 128.7, 128.7, 128.4, 128.2, 128.0 (aromatic), 79.4 (C5), 78.9 (C2), 76.0 (CH_2_Ph), 74.4 (CH_2_Ph), 71.4 (C4 and C6), 65.4 (C1 and C3), 20.3 (2 × CH_3_); HRMS (ESI-TOF) *m*/*z*: [M + Na]^+^ calculated for C_24_H_26_N_6_O_6_Na 517.1806; found 517.1809.

#### 1,3-Di(deamino)-1-azido-3-*N*-*tert*-butylcarboxy-2,5,6-tri-*O*-benzyl-3,4-oxazolidinostreptamine (**23**)

A mixture of CrCl_2_ (41 mg, 0.34 mmol) and LiI (112 mg, 0.84 mmol) in moist ethyl acetate (2.5 mL) was heated to 70 °C for 0.5 h, then the mono-ol **16** (42 mg, 0.083 mmol) dissolved in ethyl acetate (1.0 mL) was added dropwise, and stirring was continued for 4 h at same temperature. The reaction mixture was cooled to room temperature, diluted with EtOAc (30 mL), and washed with saturated aqueous Na_2_S_2_O_3_ (30 mL), and the aqueous layer was extracted with EtOAc (20 mL × 2). The organic layers were combined, washed with brine (30 mL), dried over Na_2_SO_4_, and concentrated under reduced pressure to obtain a crude product (46 mg) which was taken up in EtOAc (1.5 mL), treated with Boc_2_O (15 mg, 0.17 mmol) and DMAP (5 mg, 0.04 mmol), and stirred for 1 h. The reaction was quenched by addition of MeOH (100 μL), the solvents were removed under vacuum, and the residue was subjected to silica gel column chromatography eluting with ethyl acetate in hexane (gradient 5%, 10%, and 20%) to afford compound **23** (34 mg, 67%) as a colorless thick syrup: *R*_*f*_ = 0.30 in 20% EtOAc in hexane; [α]_D_^21^ = −86.8 (*c* = 1.0, CHCl_3_); ^1^H NMR (500 MHz, benzene-*d*_6_) δ 7.50 (d, *J* = 7.6 Hz, 2H, Ar), 7.37–7.17 (m, 10H, Ar), 7.15–7.07 (m, 3H, Ar), 4.80–4.73 (m, 2H, CH_2_Ph), 4.68–4.59 (m, 2H, CH_2_Ph), 4.51–4.45 (m, 2H, CH_2_Ph), 3.49 (t, *J* = 9.7 Hz, 1H, H3), 3.29 (t, *J* = 9.6 Hz, 1H, H5), 3.12 (t, *J* = 9.1 Hz, 1H, H1), 3.10–2.99 (m, 2H, H4 and H6), 2.93 (t, *J* = 9.3 Hz, 1H, H2), 1.43 (s, 9H, 3 × CH_3_); ^13^C{^1^H} NMR (126 MHz, benzene-*d*_6_) δ 153.5 (C=O), 151.5 (C=O), 138.1, 137.9, 137.7, 137.6, 129.3 128.4, 128.3, 128.3, 128.0, 127.8, 127.7 (aromatic), 83.8 (quat), 81.7 (C6), 79.5 (C5), 79.4 (C2), 76.0 (C4), 75.4 (CH_2_Ph), 73.8 (CH_2_Ph), 73.3 (CH_2_Ph), 68.7 (C1), 59.0 (C3), 27.5 (3 × CH_3_); HRMS (ESI-TOF) *m*/*z*: [M + Na]^+^ calculated for C_33_H_36_N_4_O_7_Na 623.2476; found 623.2464.

## Data Availability

The data underlying this study are available in the published article and its Supporting Information.

## Supporting Information Available

Copies of NMR spectra of all compounds (PDF)
