# Supplementary material for: Practical Synthesis from Streptomycin and Regioselective Partial Deprotections of (−)-(1R,2S,3R,4R,5S,6S)-1,3-Di(deamino)-1,3-diazido-2,5,6-tri-O-benzylstreptamine
Source: J Org Chem. 2024 Mar 1;89(6):4225–31. doi: 10.1021/acs.joc.3c02922 (PMC10949228; doi:10.1021/acs.joc.3c02922)

Supporting Information

for

Practical Synthesis from Streptomycin and Regioselective Partial Deprotections of (-)-1*R*,2*S*,3*R*,4*R*,5*S*,6*S*-1,3-Di(deamino)-1,3-diazido-2,5,6-tri-*O*-benzylstreptamine

Niteshlal Kasdekar,[a] Michael R Spieker[a,b] and David Crich[a,c,d,*]

a) Department of Pharmaceutical and Biomedical Sciences, University of Georgia, 250 West Green Street, Athens, GA 30602, USA

b) Department of Biochemistry and Molecular Biology, University of Georgia, 120 East Green Street, Athens, GA 30602, USA

c) Department of Chemistry, University of Georgia, 302 East Campus Road, Athens, GA 30602, USA

d) Complex Carbohydrate Research Center, University of Georgia, 315 Riverbend Road, Athens, GA 30602, USA

Email: David.Crich@uga.edu

Table of contents

**Compound**	**Spectral Charts**
1,3-Bis(deamino)-1,3-diazido-dihydrostreptomycin **(11)**	S3-S6
N'-Methyl-1,3-bis(deamino)-1,3-diazido-dihydrostreptomycin **(12)**	S7-S11
1,3-Bis(deamino)-1,3-diazido-2,5,6-tri-*O*-benzyl-4-*O*-(3',6'-di-*O*-benzyl-2'-*O*-(2''-N-benzyl-3'',4'',6''-tri-*O*-benzyl-dihydrostreptomycin **(13)**	S12-S15
1,3-Di(deamino)-1,3-diazido-2,5,6-tri-*O*-benzylstreptamine **(14)**	S16-S19
Methyl Streptobiosaminide **(15)**	S20-S23
4-*O*-Acetyl-1,3-di(deamino)-1,3-diazido-2,5,6-tri-*O*-benzylstreptamine **(16)**	S24-S27
1,3-Di(deamino)-1,3-diazido-2,6-di-*O*-benzylstreptamine (**17**)	S28-S31
4,5-Di-*O*-acetyl-1,3-di(deamino)-1,3-diazido-2,6-di-*O*-benzylstreptamine (**18**)	S32-S35
1,3-Di(deamino)-1,3-diazido-5,6-di-*O*-benzylstreptamine (**19**)	S36-S39
1,3-Di(deamino)-1,3-diazido-2,5-di-*O*-benzylstreptamine (**20**)	S40-S43
2,4-Di-*O*-acetyl-1,3-di(deamino)-1,3-diazido-5,6-di-*O*-benzylstreptamine (**21**)	S44-S47
4,6-Di-*O*-acetyl-1,3-di(deamino)-1,3-diazido-2,5-di-*O*-benzylstreptamine (**22**)	S48-S51
1,3-Di(deamino)-1-azido-3-*N*-tert-butylcarboxy-2,5,6-tri-*O*-benzyl-3,4-oxazolidino-streptamine (**23**)	S52-S55

Spectral Charts

^{1}H NMR (900 MHz, CD_3OD) spectrum of 1,3-bis(deamino)-1,3-diazido-dihydrostreptomycin (**11**)

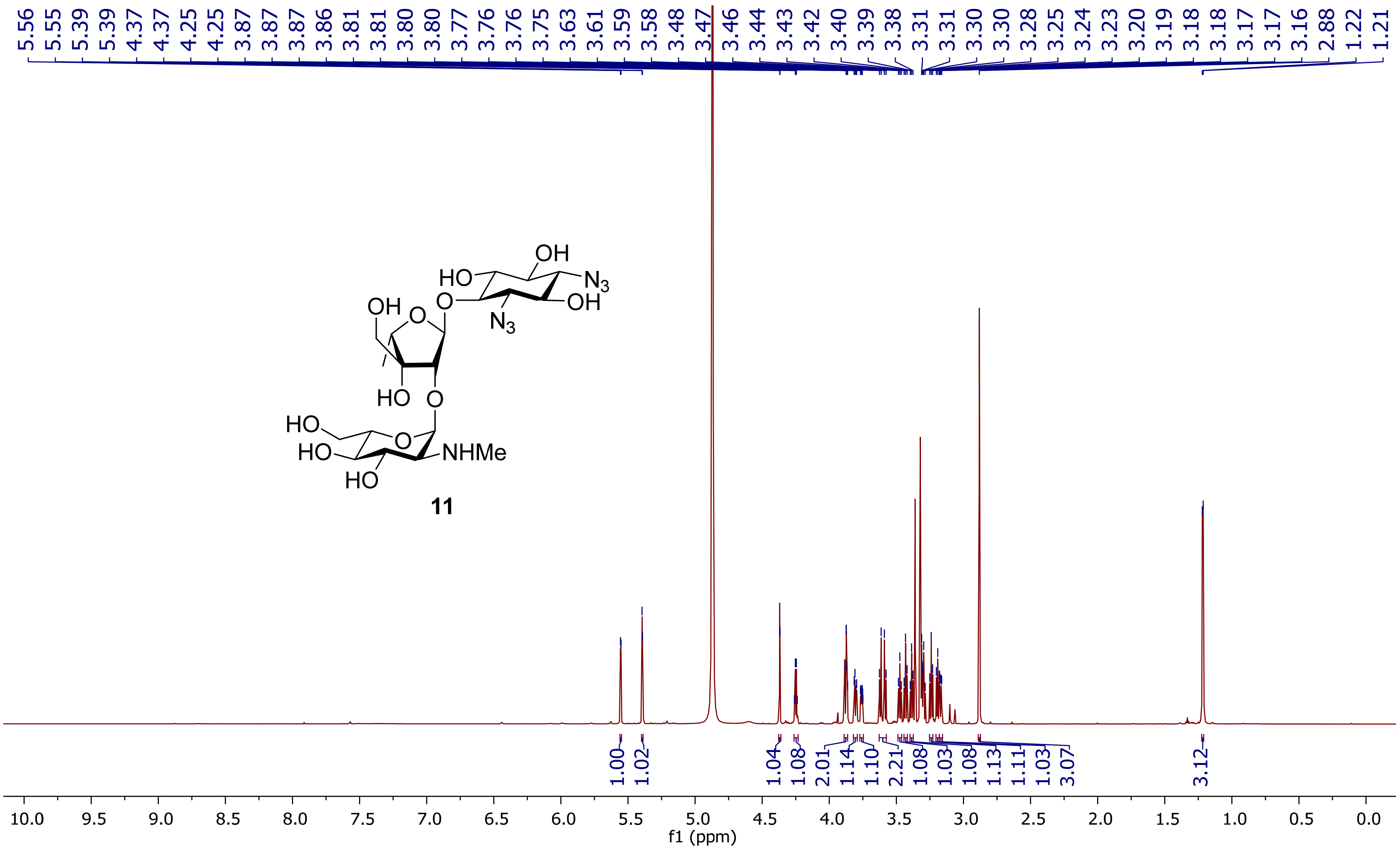

$^{13}C\{^{1}H\}$ NMR (226 MHz, CD_3OD) spectrum of 1,3-bis(deamino)-1,3-diazido-dihydrostreptomycin (**11**)

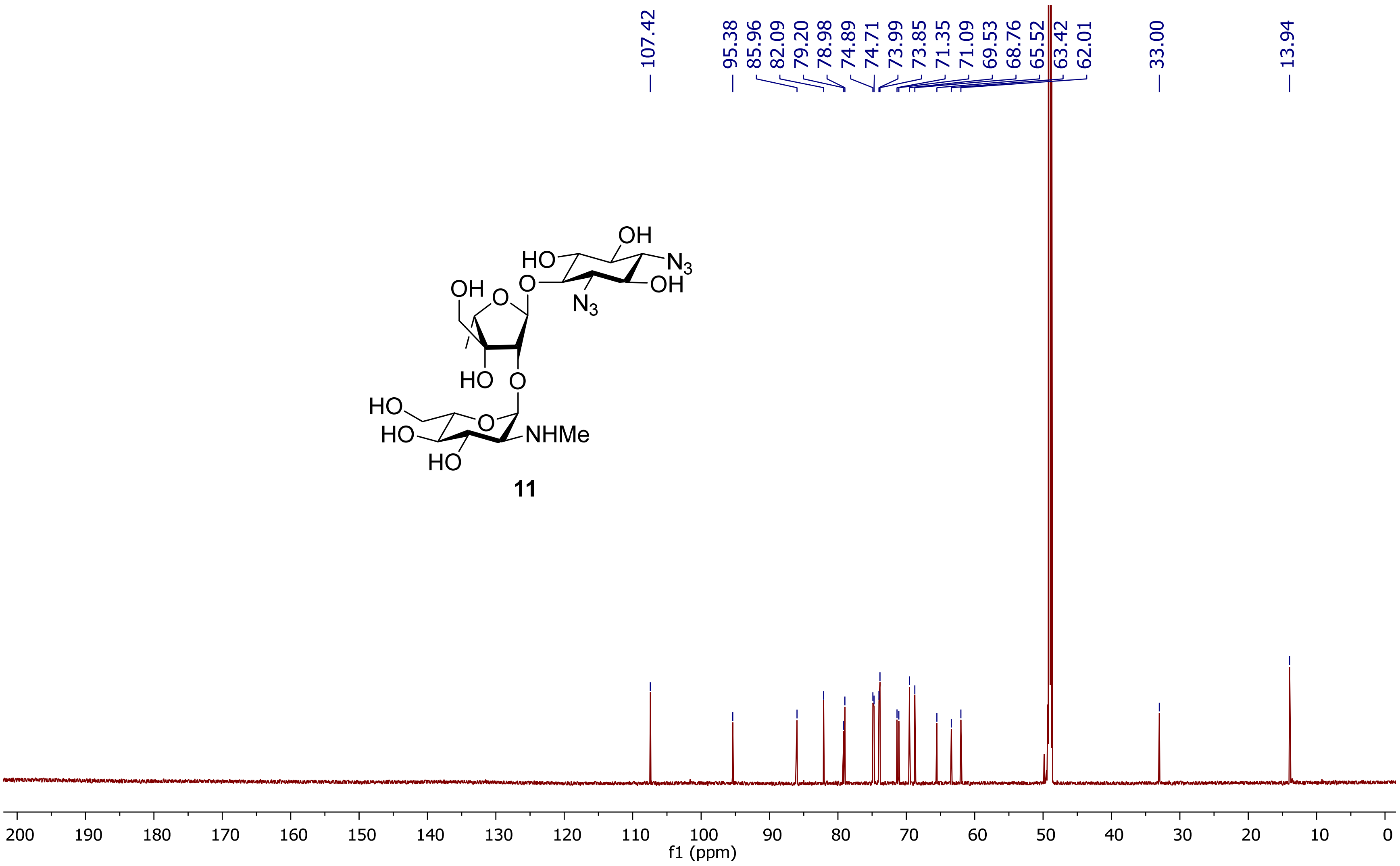

COSY NMR (900 MHz, CD_3OD) spectrum 1,3-bis(deamino)-1,3-diazido-dihydrostreptomycin (**11**)

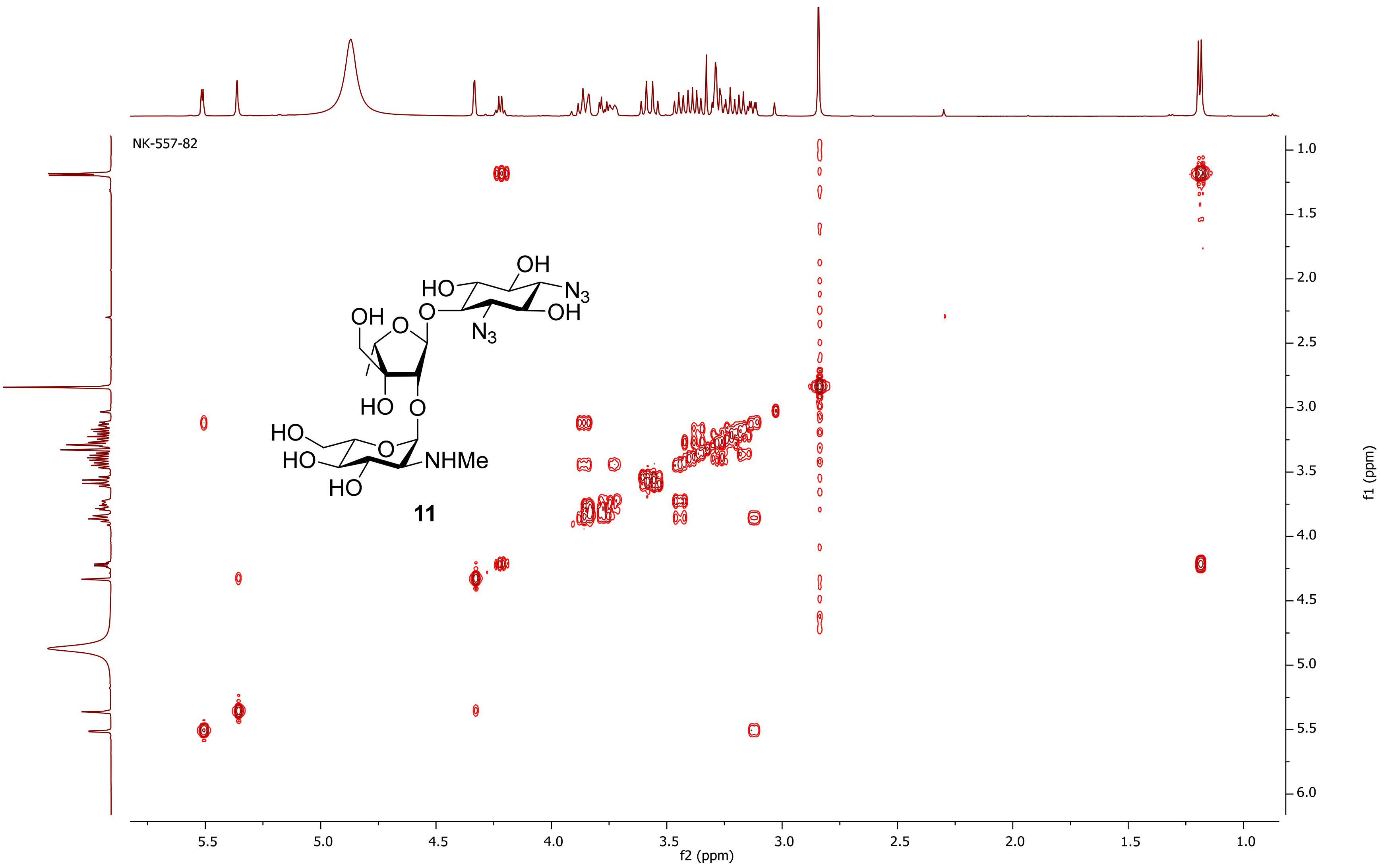

HSQC NMR (900 MHz, CD_3OD) spectrum of 1,3-bis(deamino)-1,3-diazido-dihydrostreptomycin (**11**)

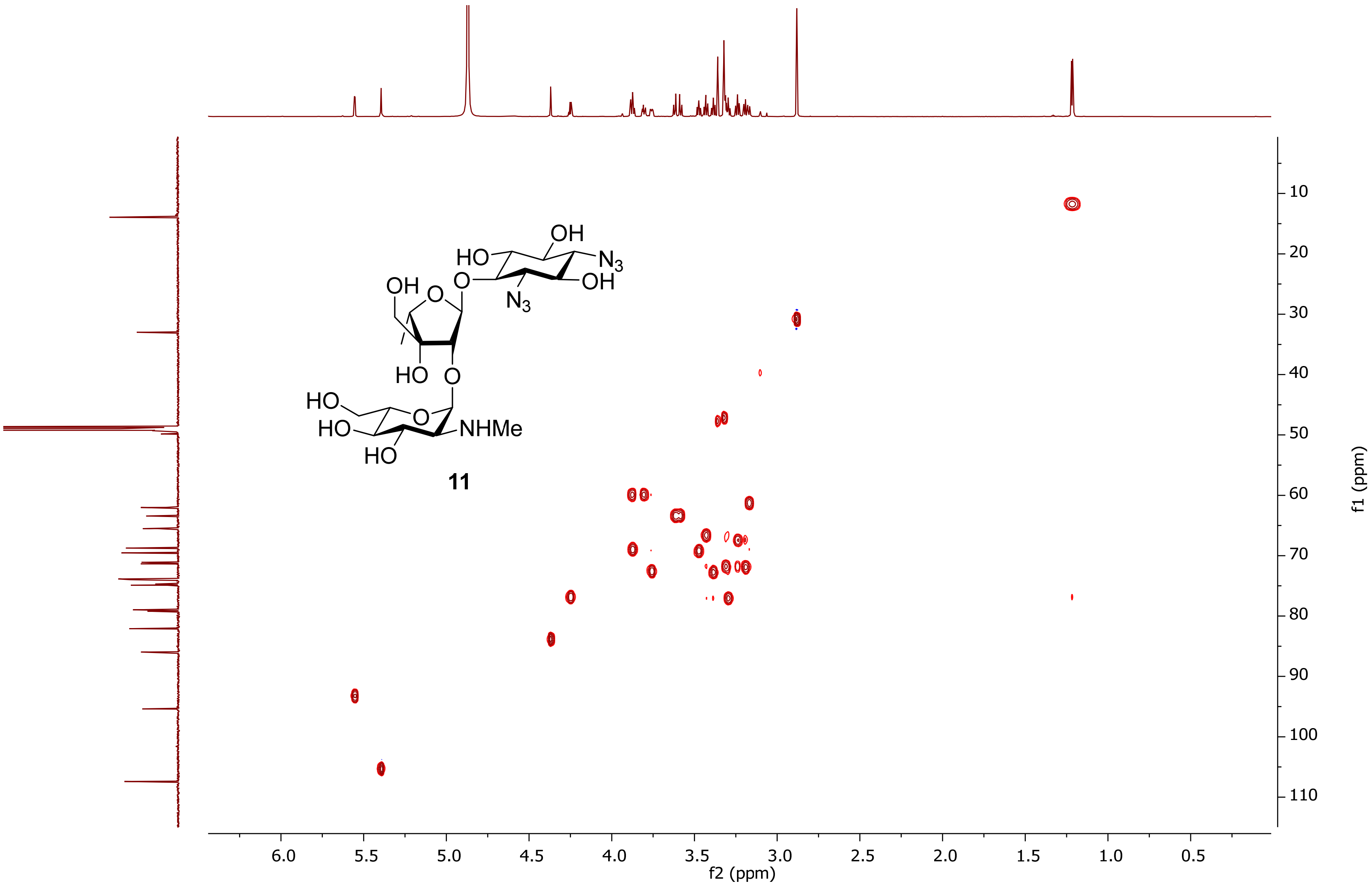

^{1}H NMR (900 MHz, CD_3OD) spectrum of *N'*-methyl-1,3-bis(deamino)-1,3-diazido-dihydrostreptomycin (**12**)

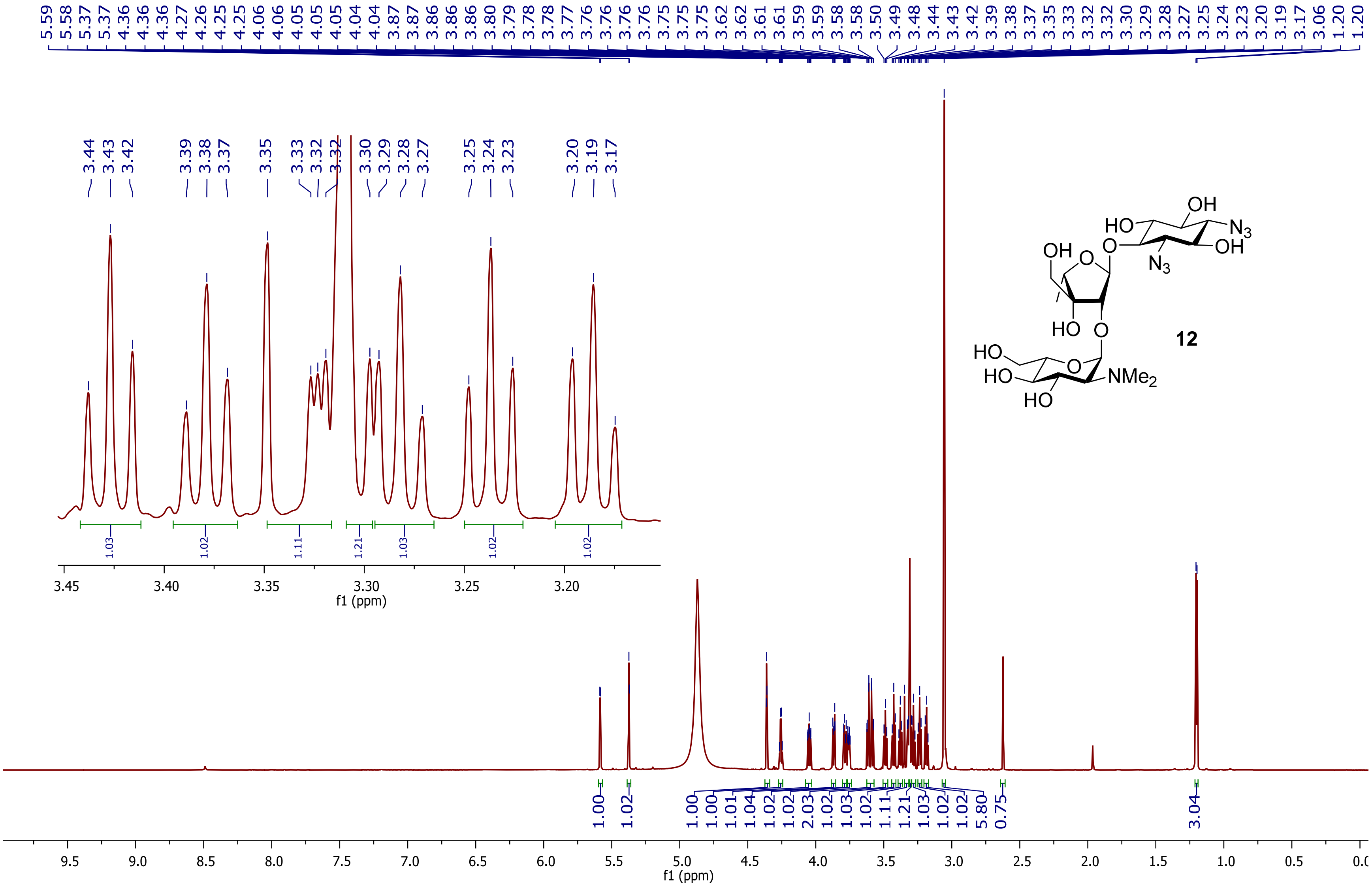

$^{13}C\{^1H\}$ NMR (226 MHz, CD_3OD) spectrum of *N'*-methyl-1,3-bis(deamino)-1,3-diazido-dihydrostreptomycin (**12**)

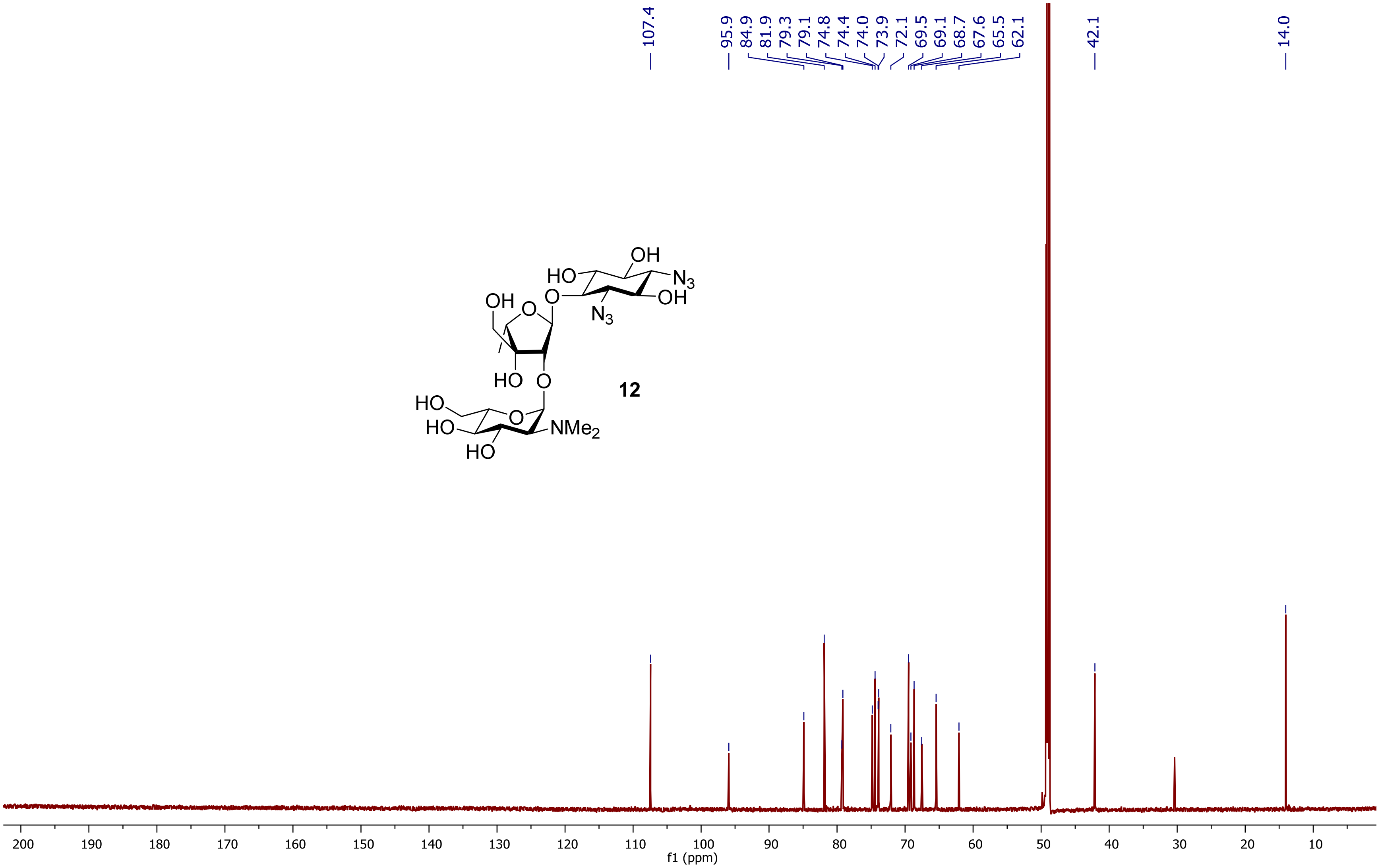

COSY NMR (900 MHz, CD_3OD) spectrum of *N'*-methyl-1,3-bis(deamino)-1,3-diazido-dihydrostreptomycin (**12**)

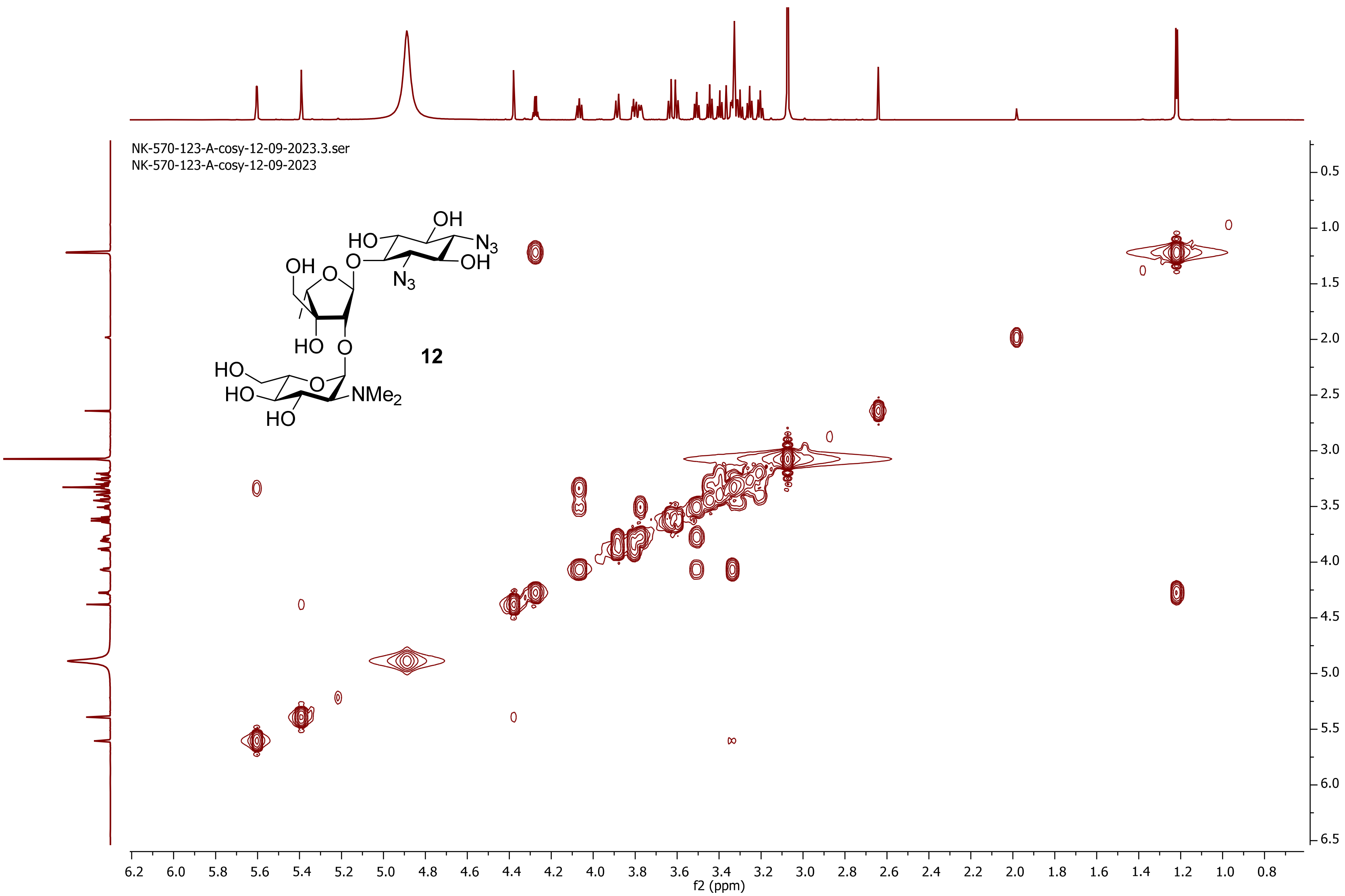

HSQC NMR (900 MHz, CD_3OD) spectrum of *N'*-methyl-1,3-bis(deamino)-1,3-diazido-dihydrostreptomycin (**12**)

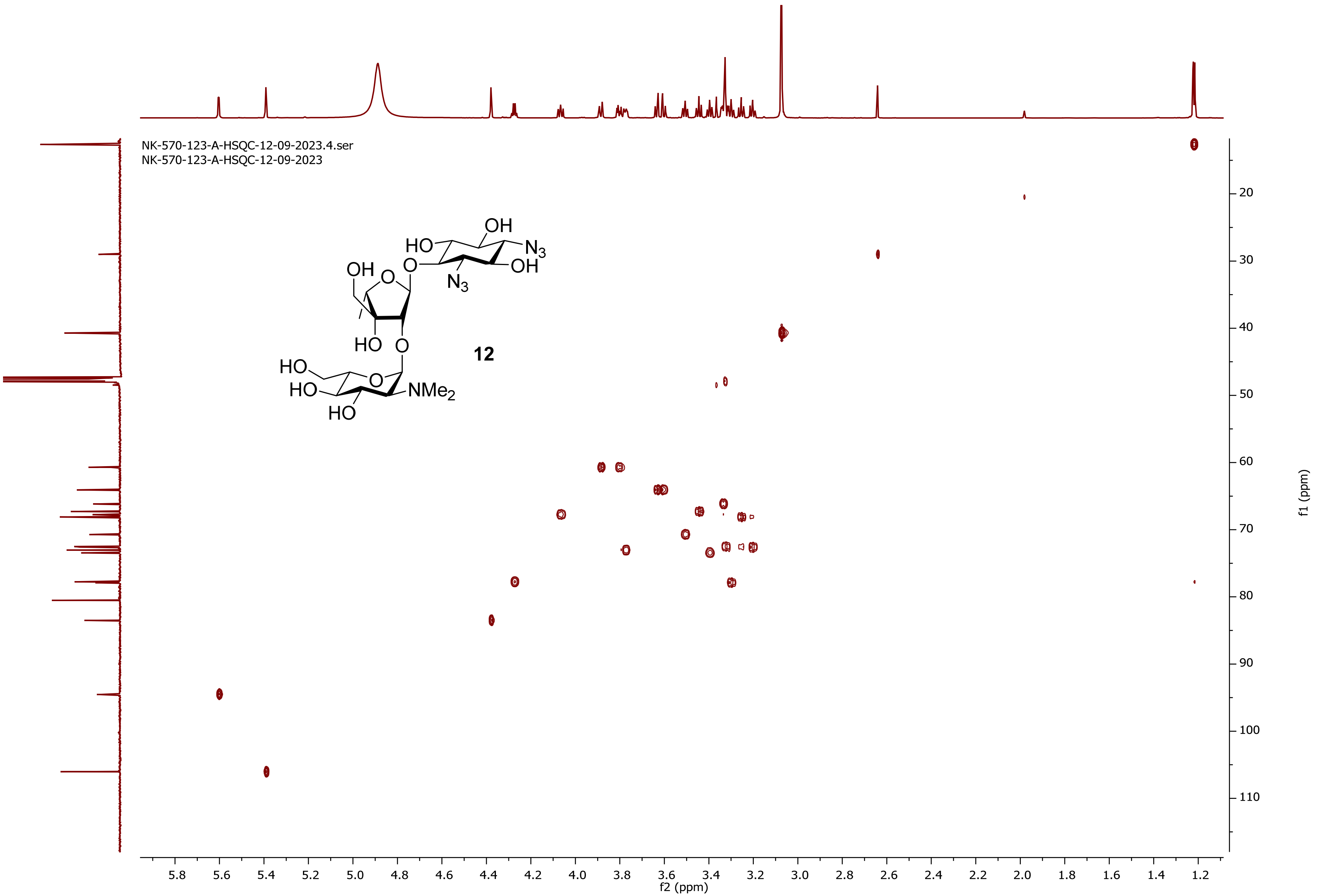

HMBC NMR (900 MHz, CD_3OD) spectrum of *N'*-methyl-1,3-bis(deamino)-1,3-diazido-dihydrostreptomycin (**12**)

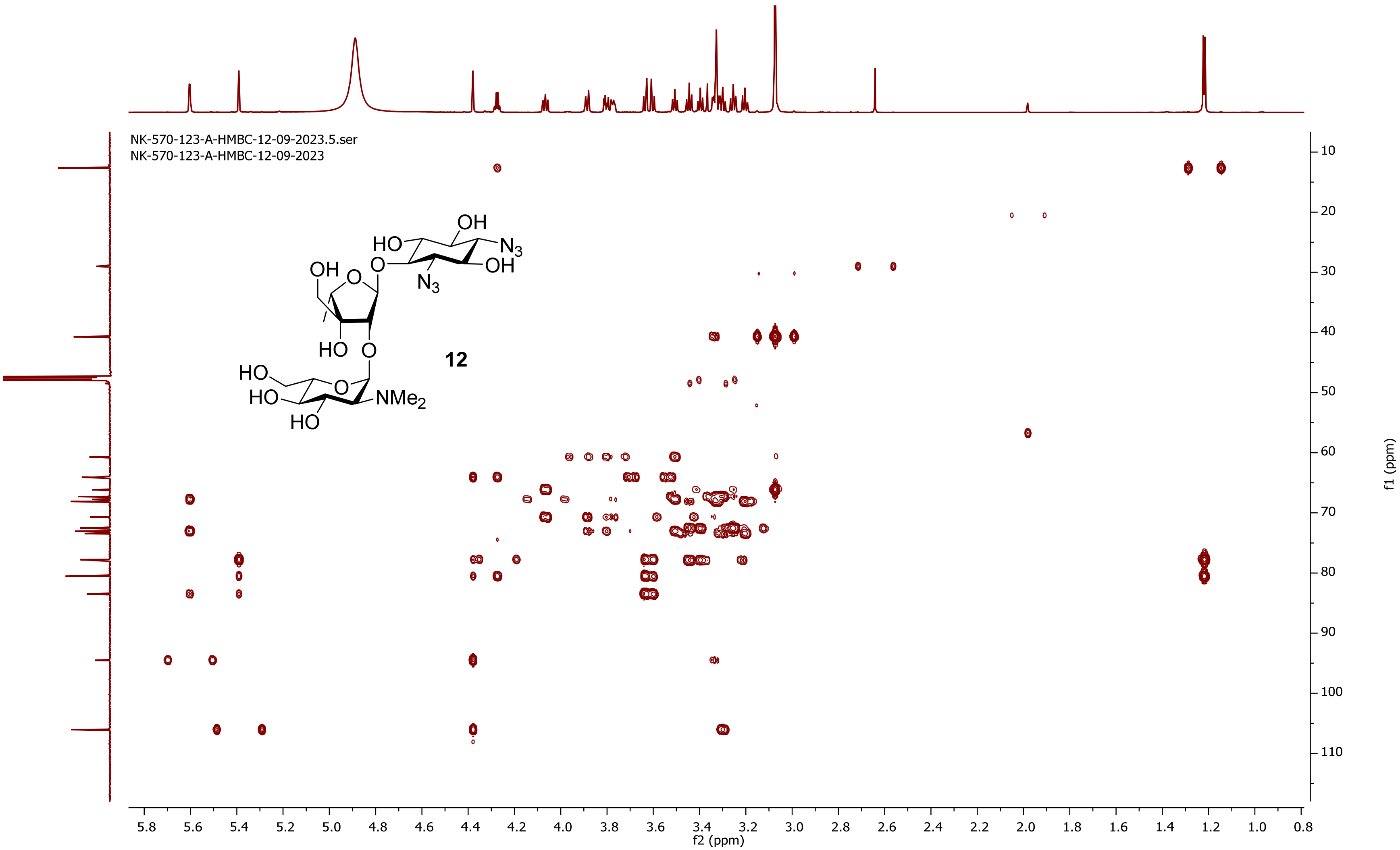

^1H NMR (500 MHz, $CDCl_3$) spectrum of 1,3-bis(deamino)-1,3-diazido-2,5,6-tri-*O*-benzyl-4-*O*-(3',6'-di-*O*-benzyl-2'-*O*-(2''-N-benzyl-3'',4'',6''-tri-*O*-benzyl-dihydrostreptomycin (**13**)

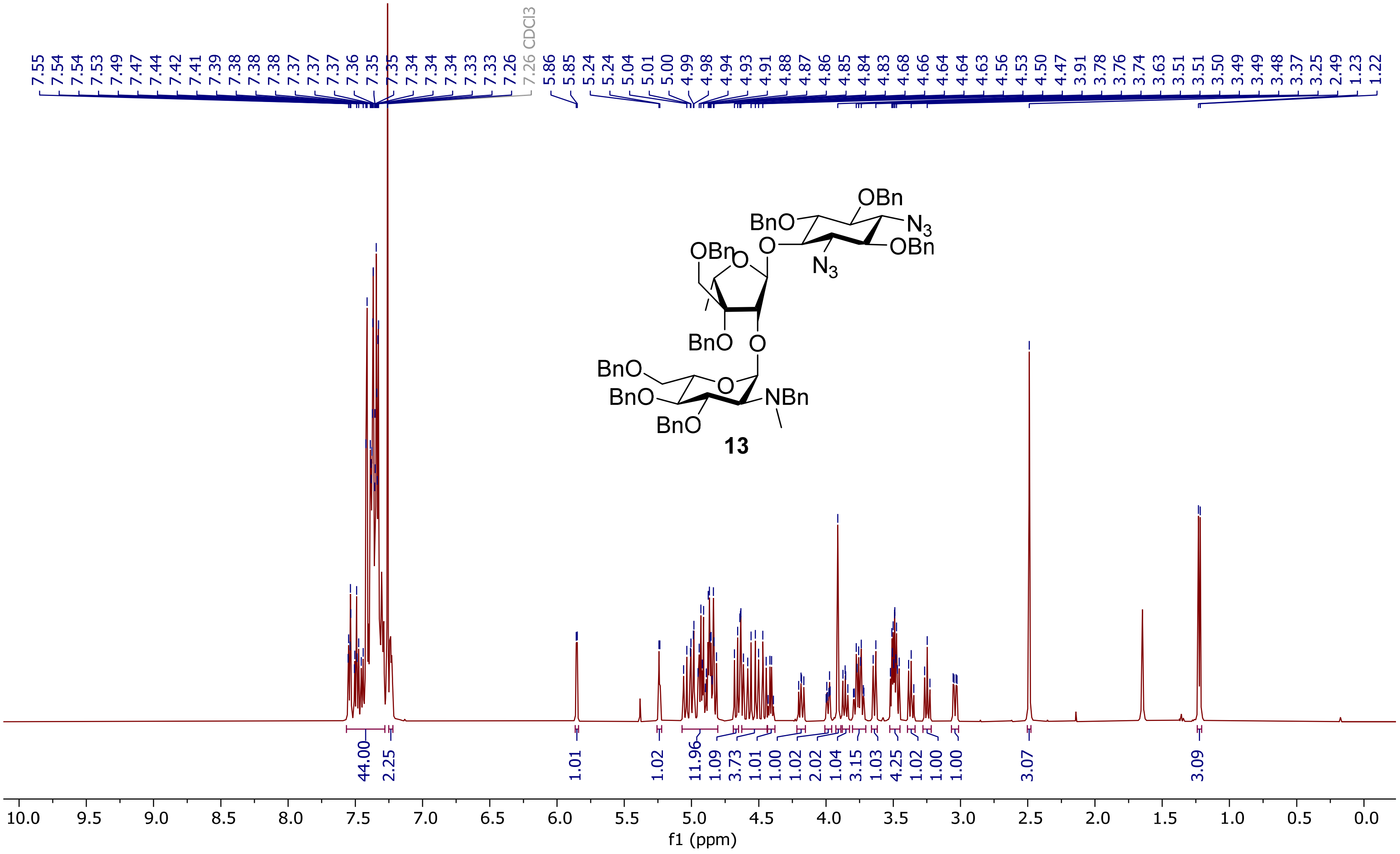

$^{13}C\{^{1}H\}$ NMR (126 MHz, $CDCl_3$) spectrum of 1,3-bis(deamino)-1,3-diazido-2,5,6-tri-*O*-benzyl-4-*O*-(3',6'-di-*O*-benzyl-2'-*O*-(2''-*N*-benzyl-3'',4'',6''-tri-*O*-benzyl-dihydrostreptomycin (**13**)

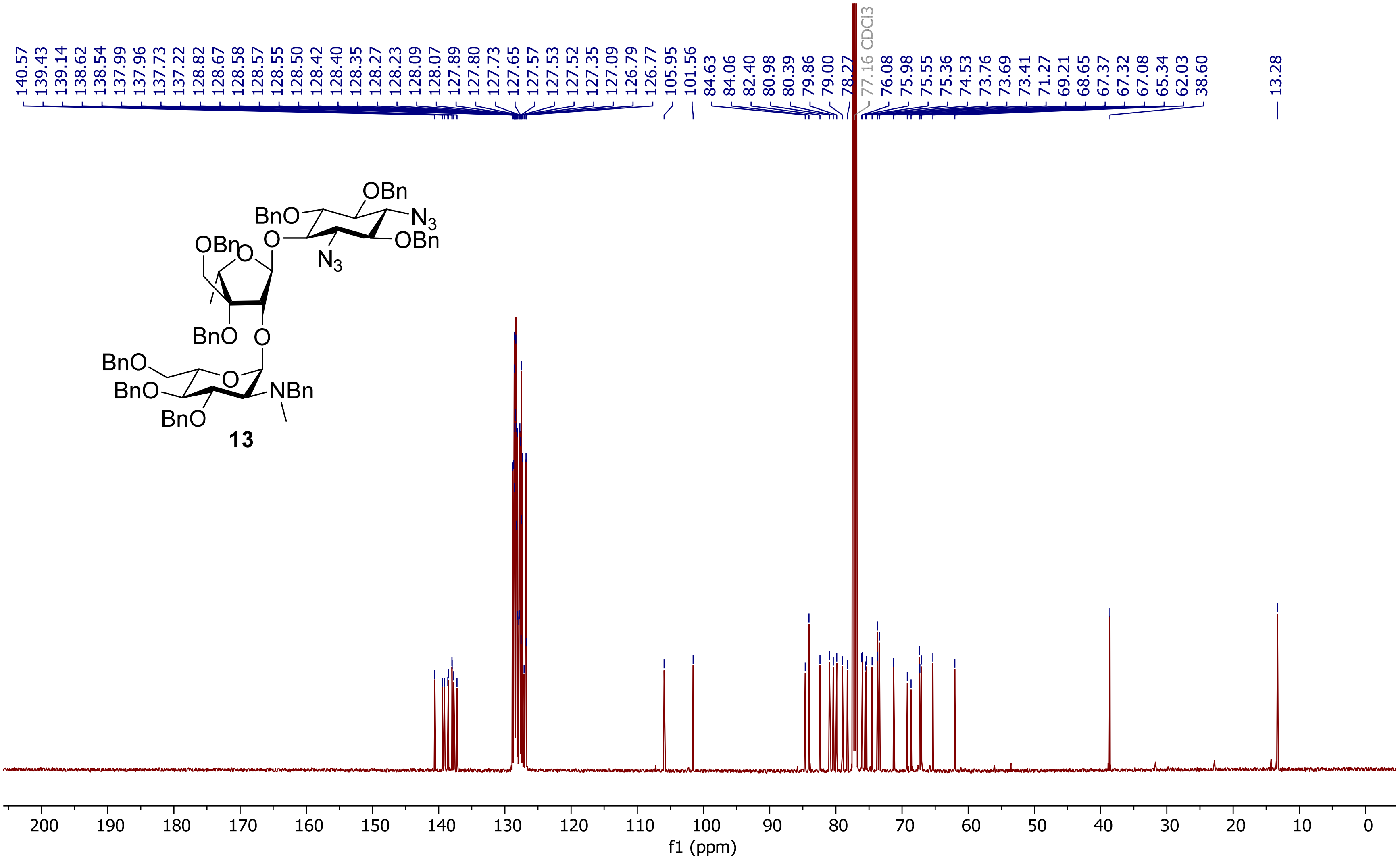

COSY NMR (500 MHz, $CDCl_3$) spectrum of 1,3-bis(deamino)-1,3-diazido-2,5,6-tri-*O*-benzyl-4-*O*-(3',6'-di-*O*-benzyl-2'-*O*-(2''-*N*-benzyl-3'',4'',6''-tri-*O*-benzyl-dihydrostreptomycin (**13**)

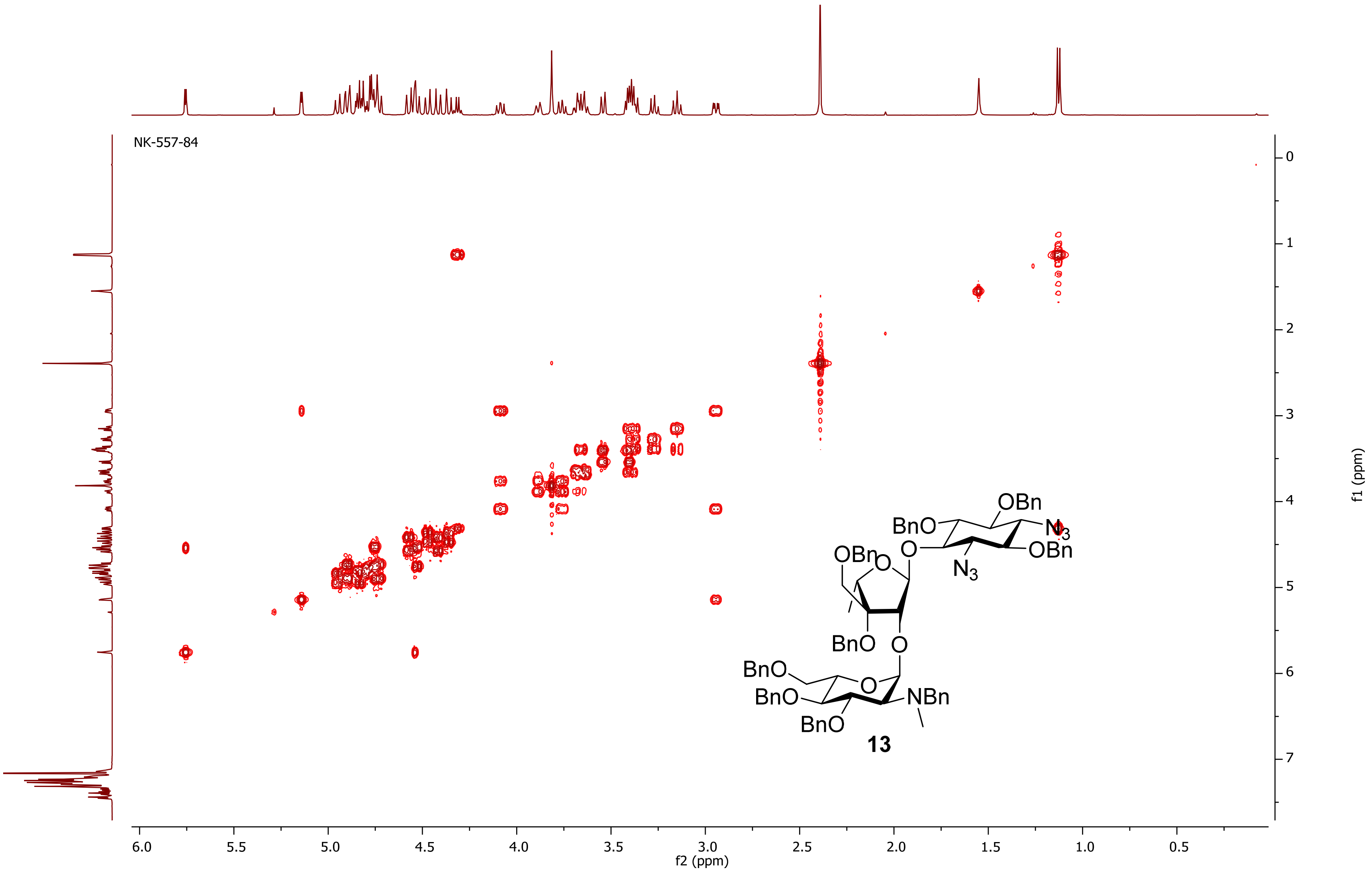

HSQC NMR (500 MHz, $CDCl_3$) spectrum of 1,3-bis(deamino)-1,3-diazido-2,5,6-tri-*O*-benzyl-4-*O*-(3',6'-di-*O*-benzyl-2'-*O*-(2''-*N*-benzyl-3'',4'',6''-tri-*O*-benzyl-dihydrostreptomycin (**13**)

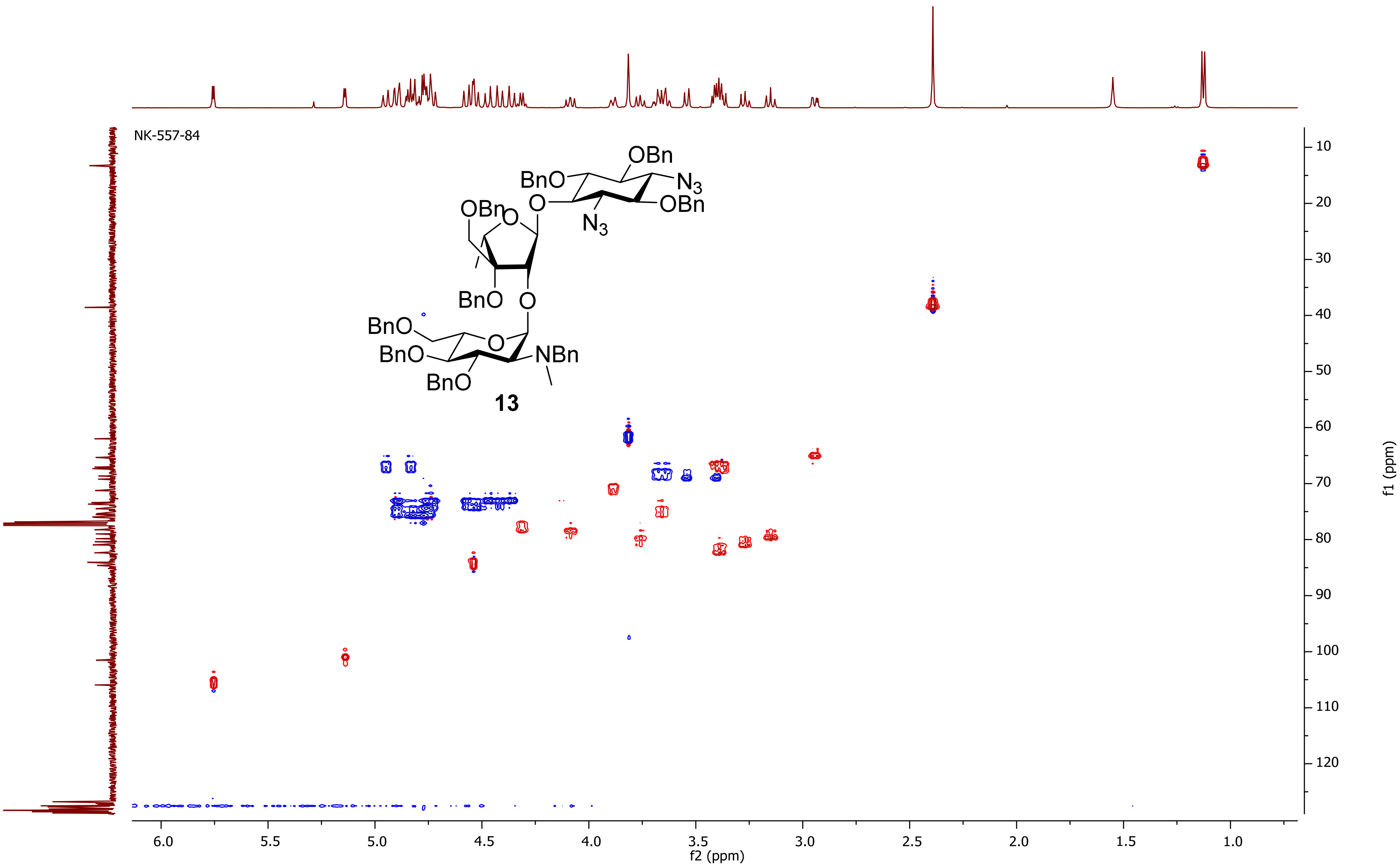

^{1}H NMR (900 MHz, $CDCl_3$) spectrum of 1,3-bis(deamino)-1,3-diazido-4-O-acetyl-2,5,6-tri-*O*-benzylstreptamine (**14**)

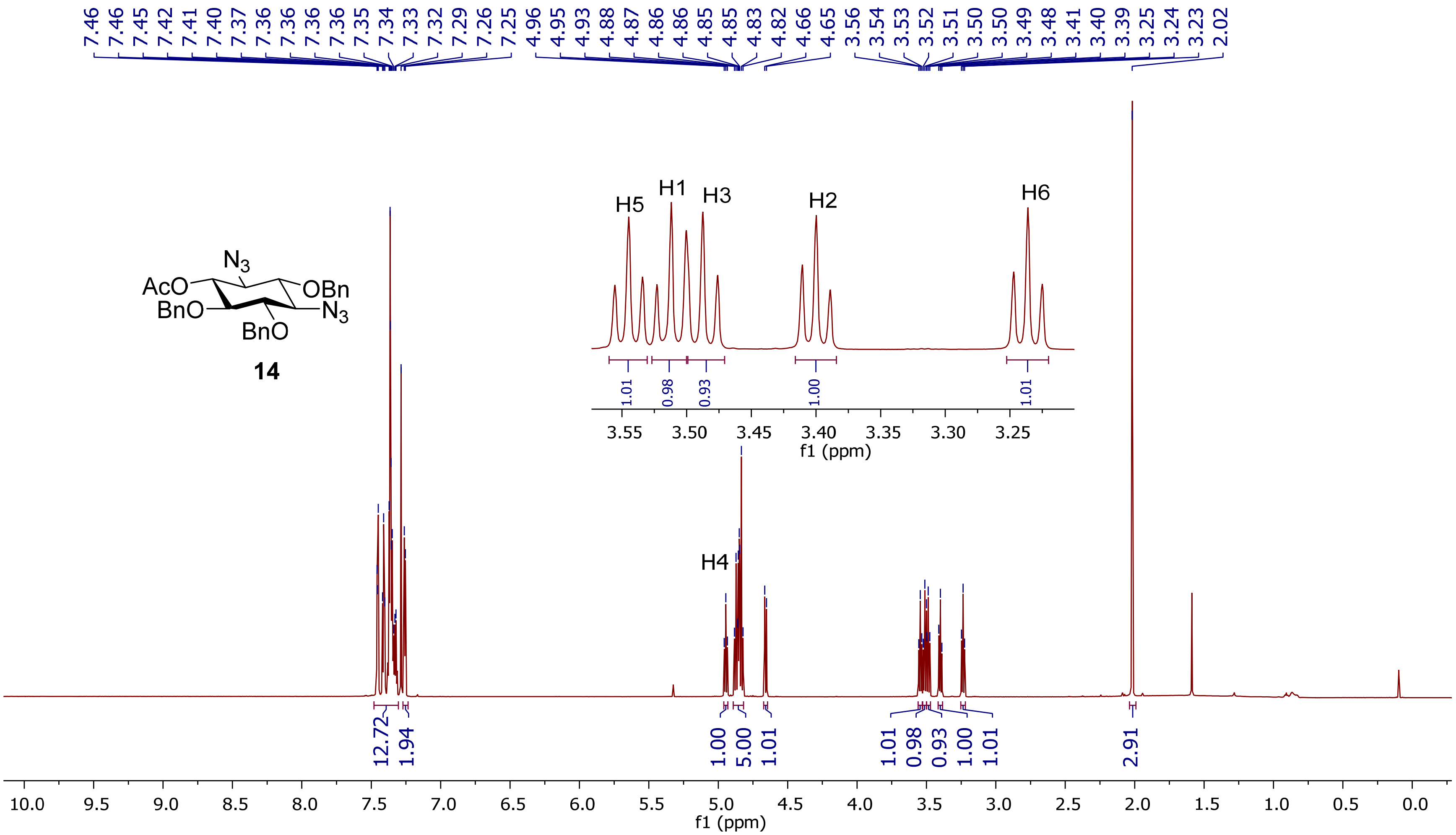

$^{13}C\{^{1}H\}$ NMR (226 MHz, $CDCl_3$) spectrum of 4-O-acetyl-1,3-di(deamino)-1,3-diazido-2,5,6-tri-*O*-benzylstreptamine (**14**)

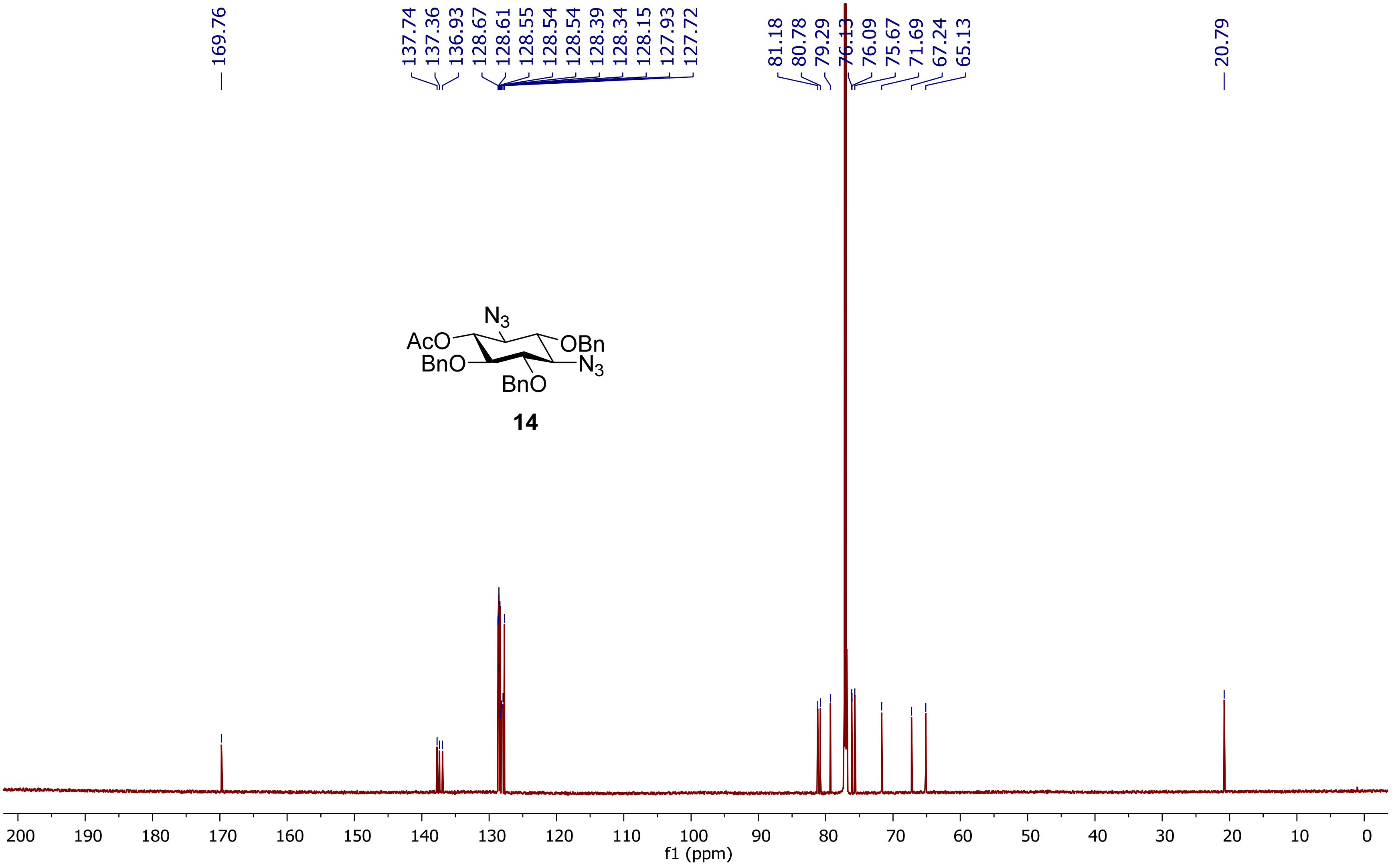

COSY NMR (900 MHz, $CDCl_3$) spectrum of 4-O-acetyl-1,3-di(deamino)-1,3-diazido-2,5,6-tri-*O*-benzylstreptamine (**14**)

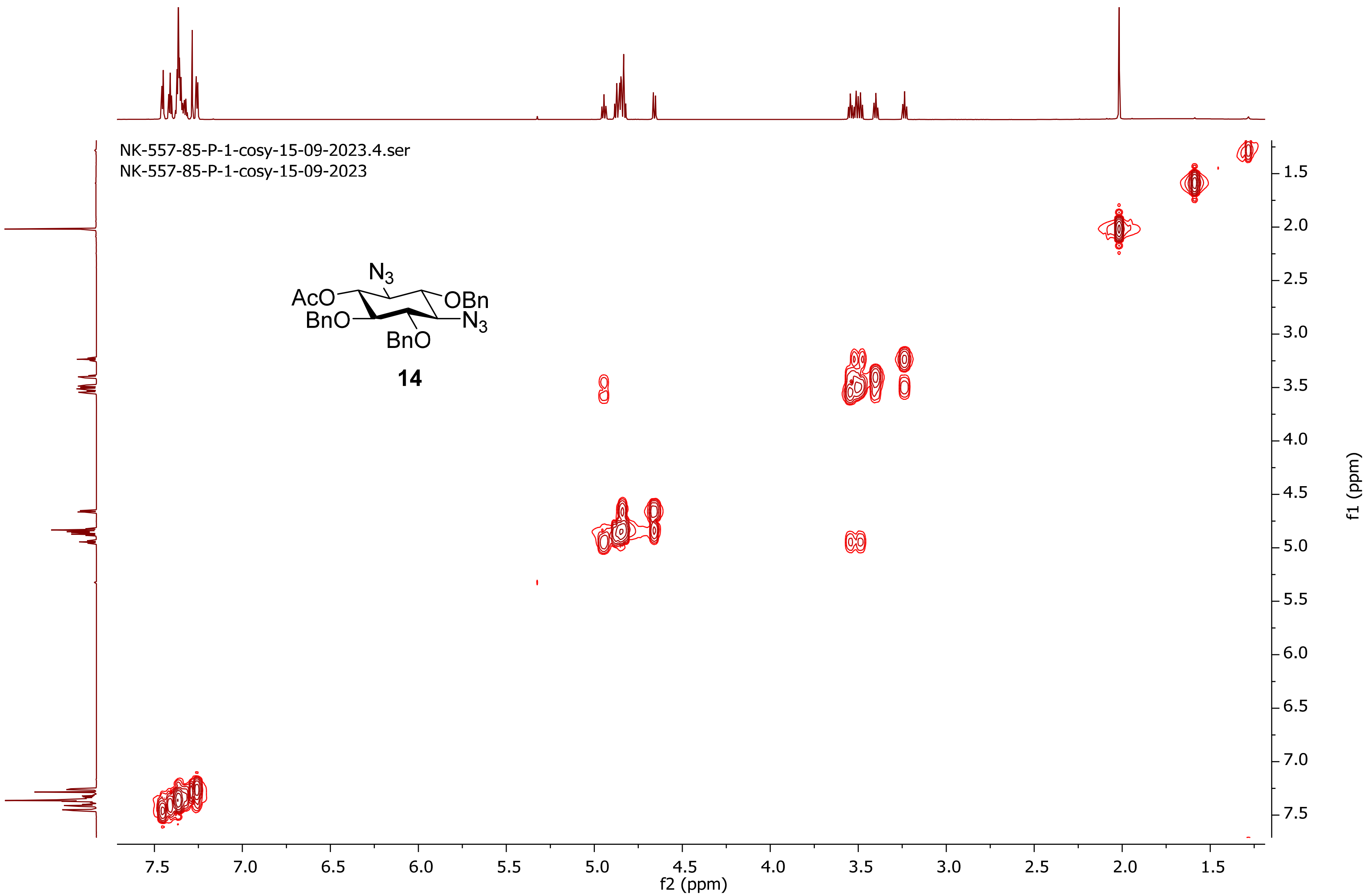

HSQC NMR (900 MHz, $CDCl_3$) spectrum of 4-O-acetyl-1,3-di(deamino)-1,3-diazido-2,5,6-tri-*O*-benzylstreptamine (**14**)

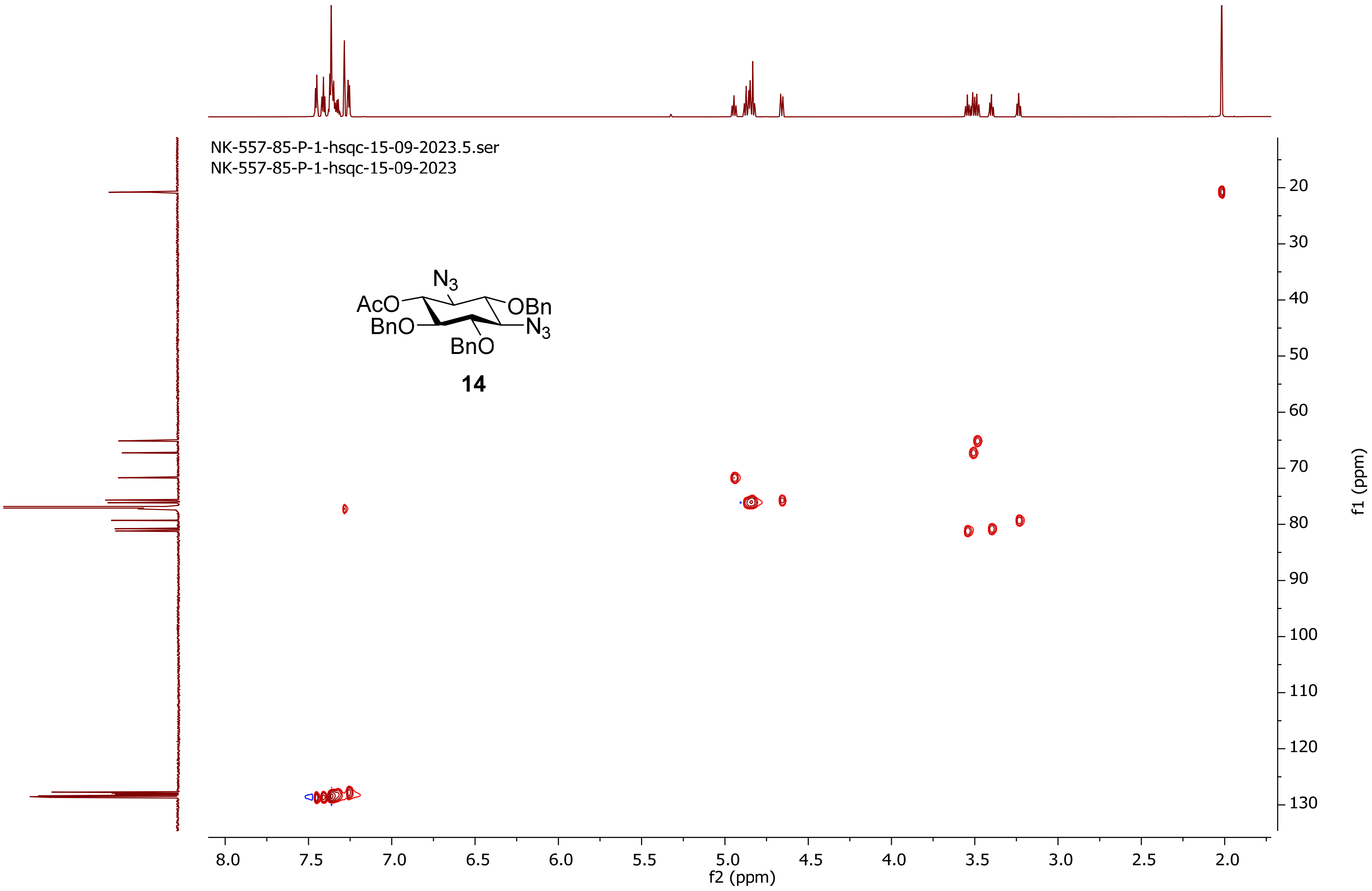

^{1}H NMR (500 MHz, $CDCl_3$) spectrum of Methyl 3',6'-di-*O*-benzyl-2-*O*-(2''-*N*-benzyl-3'',4'',6''-tri-*O*-benzyl)-β-streptobiosaminide (**15**)

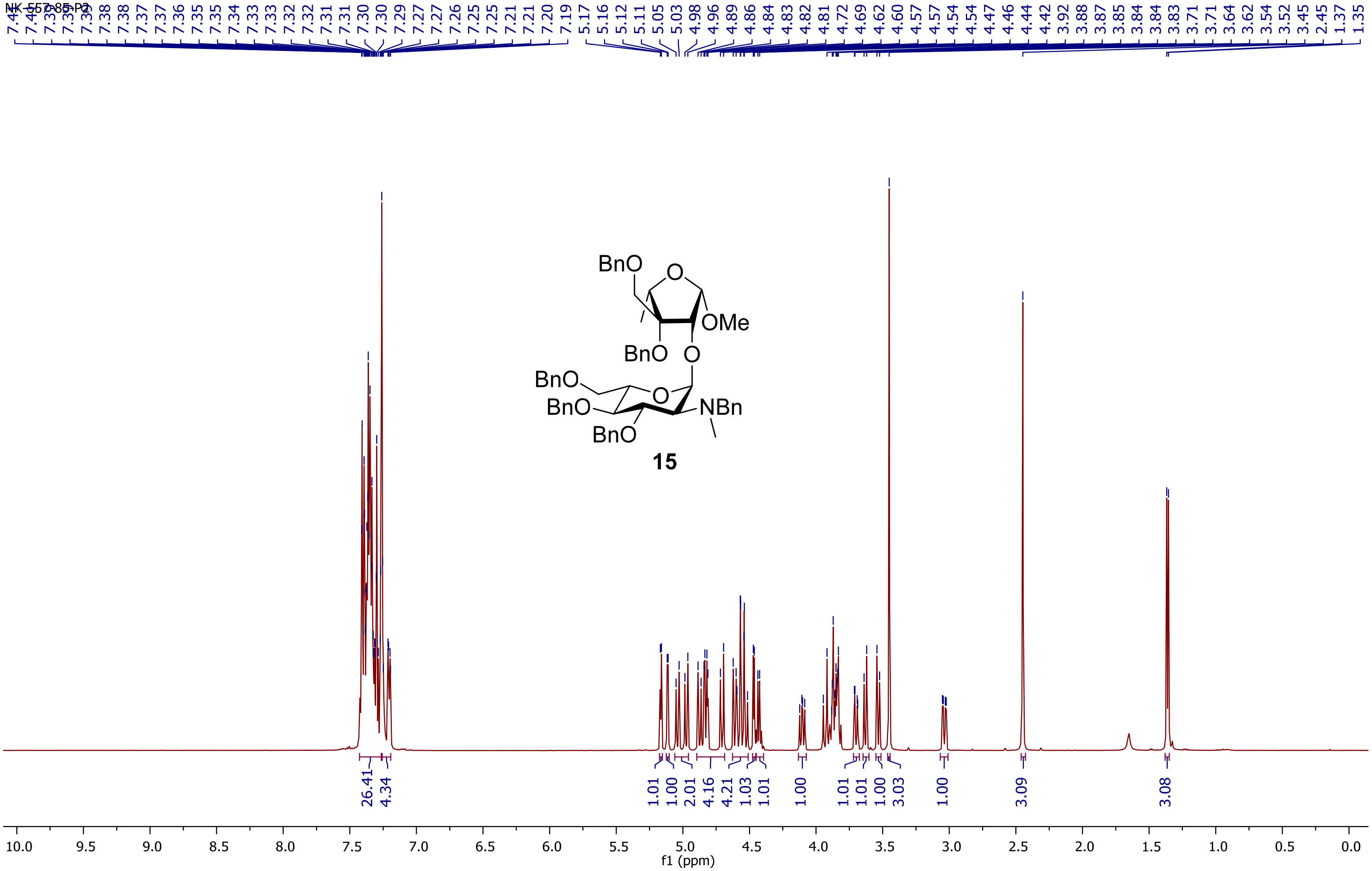

$^{13}C\{^{1}H\}$ NMR (126 MHz, $CDCl_3$) spectrum of Methyl 3',6'-di-*O*-benzyl-2-*O*-(2''-*N*-benzyl-3'',4'',6''-tri-*O*-benzyl)-*β*-streptobiosaminide (**15**)

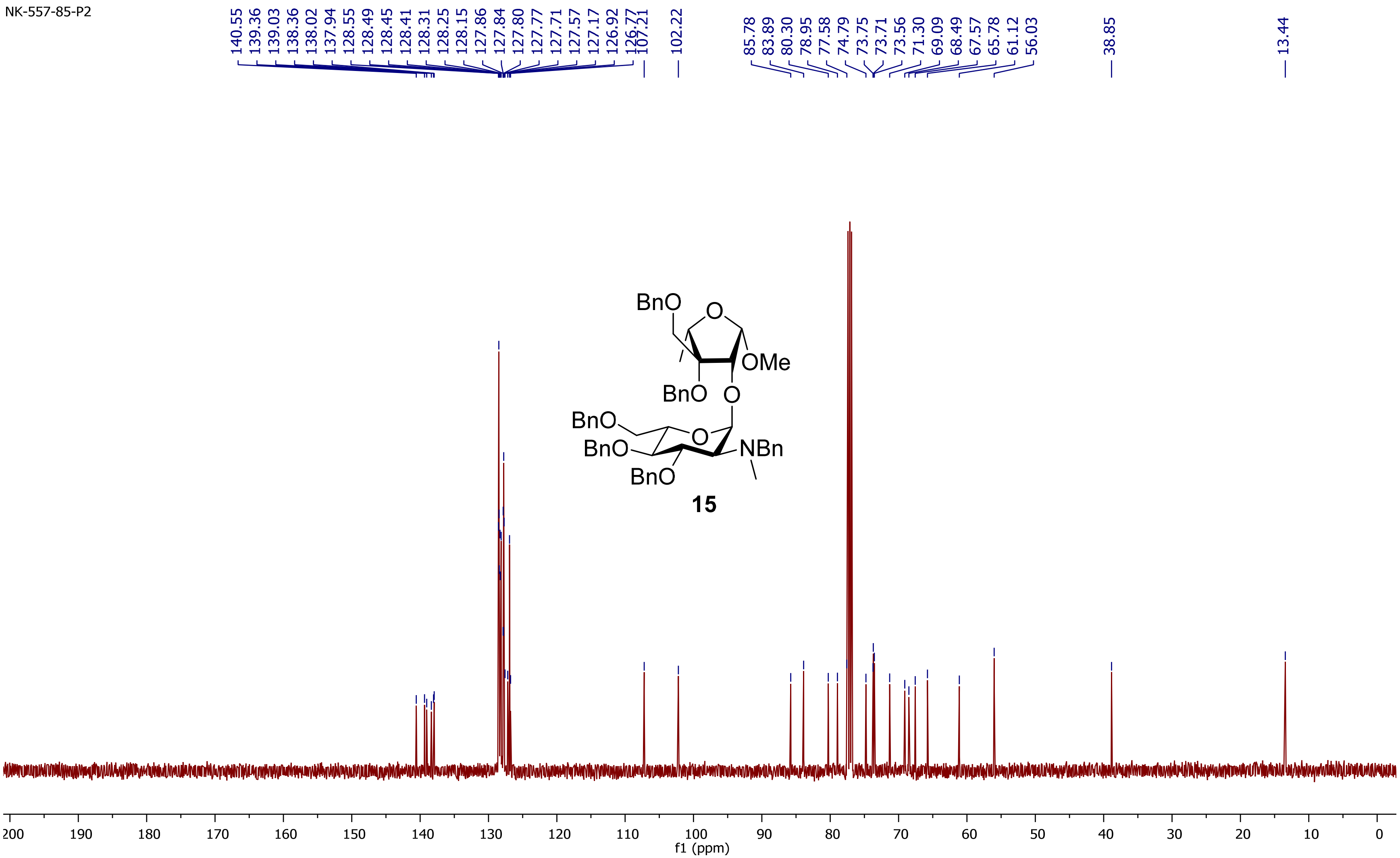

COSY NMR (500 MHz, $CDCl_3$) spectrum of Methyl 3’,6’-di-*O*-benzyl-2-*O*-(2’’-*N*-benzyl-3’’,4’’,6’’-tri-*O*-benzyl)-*β*-streptobiosaminide (**15**)

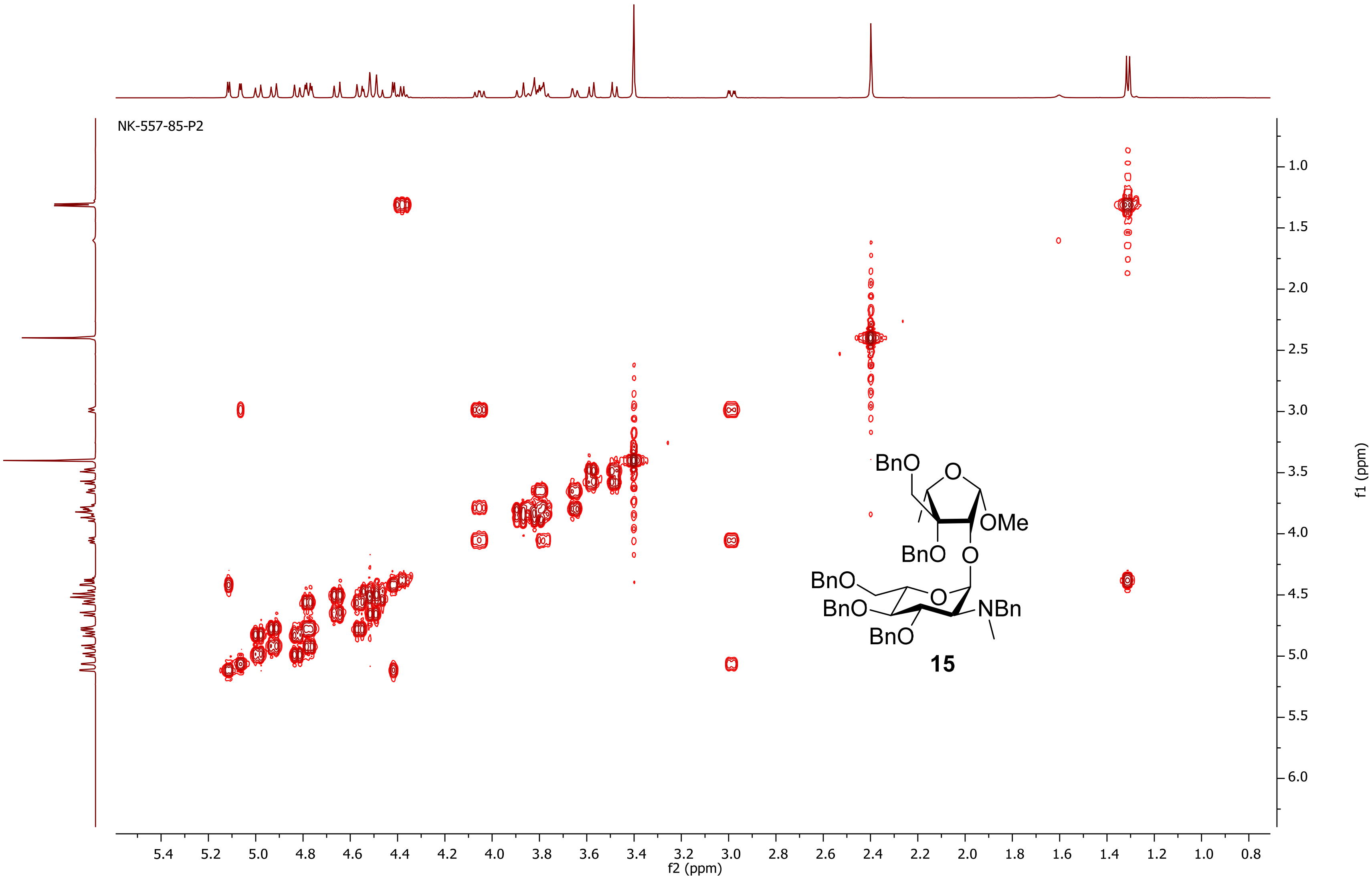

HSQC NMR (500 MHz, $CDCl_3$) spectrum of Methyl 3’,6’-di-*O*-benzyl-2-*O*-(2’’-*N*-benzyl-3’’,4’’,6’’-tri-*O*-benzyl)-β-streptobiosaminide (**15**)

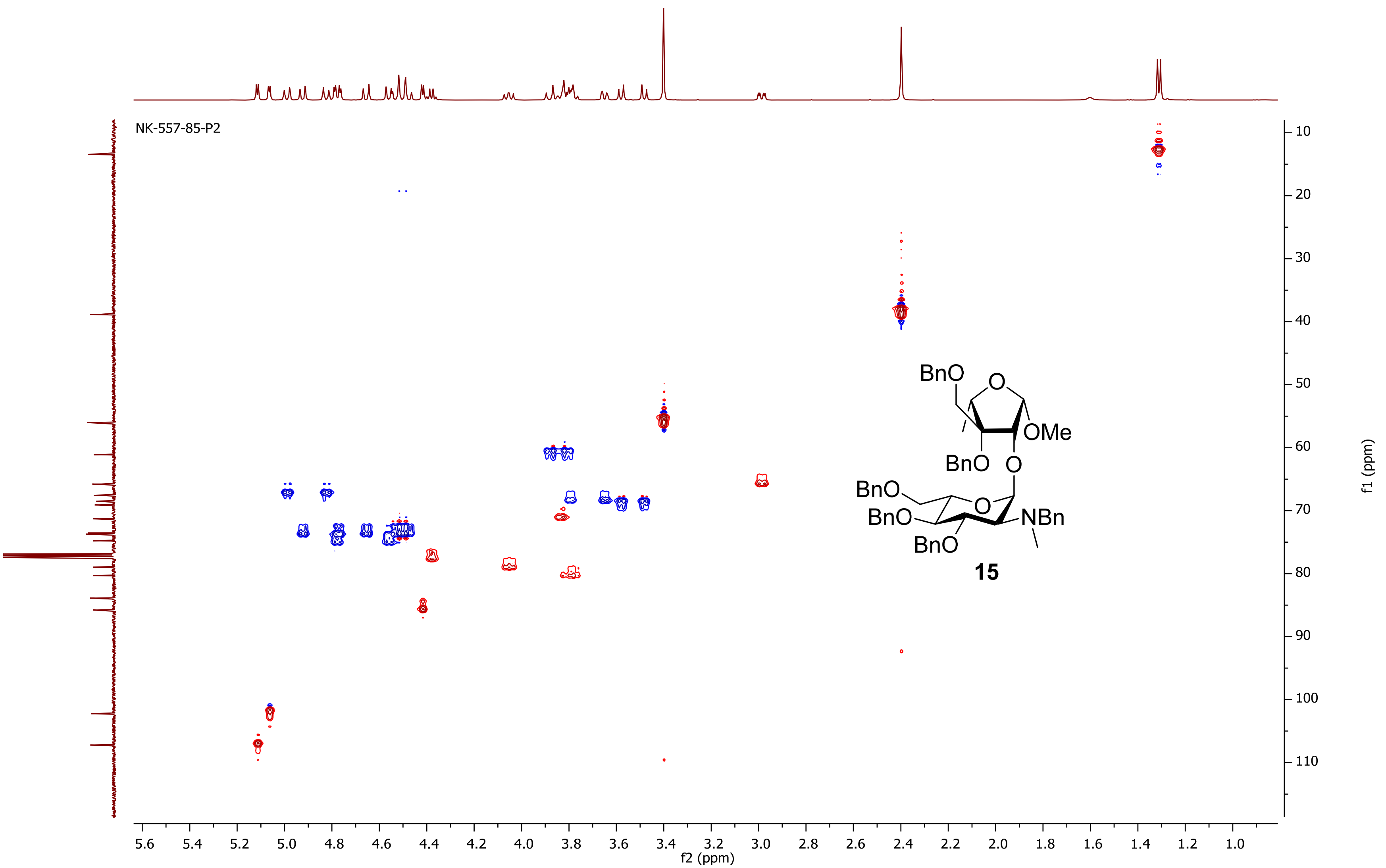

^{1}H NMR (900 MHz, $CDCl_3$) spectrum of 1,3-Di(deamino)-1,3-diazido-2,5,6-tri-*O*-benzyl streptamine (**16**)

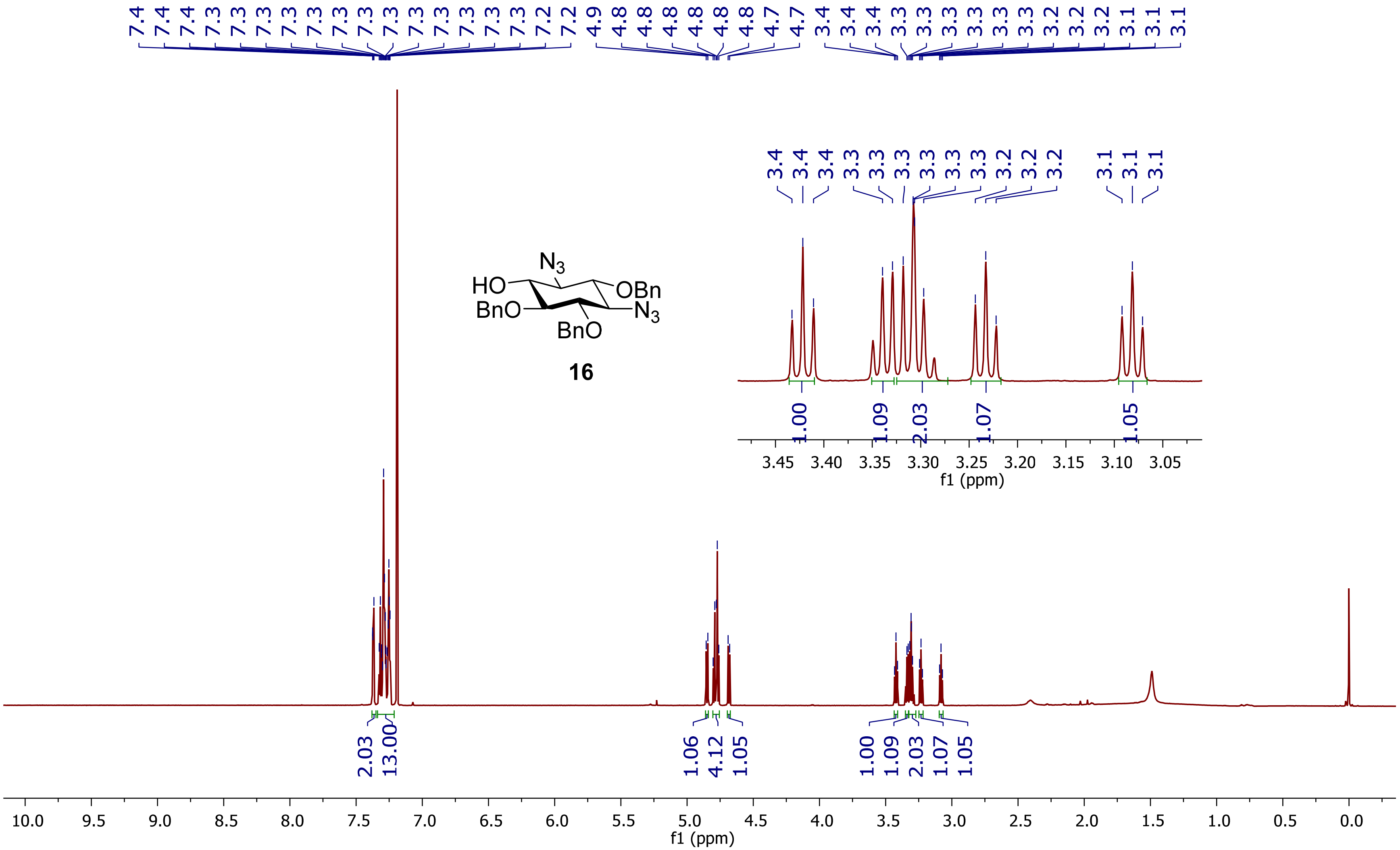

$^{13}C\{^{1}H\}$ NMR (226 MHz, $CDCl_3$) spectrum of 1,3-Di(deamino)-1,3-diazido-2,5,6-tri-*O*-benzyl streptamine (**16**)

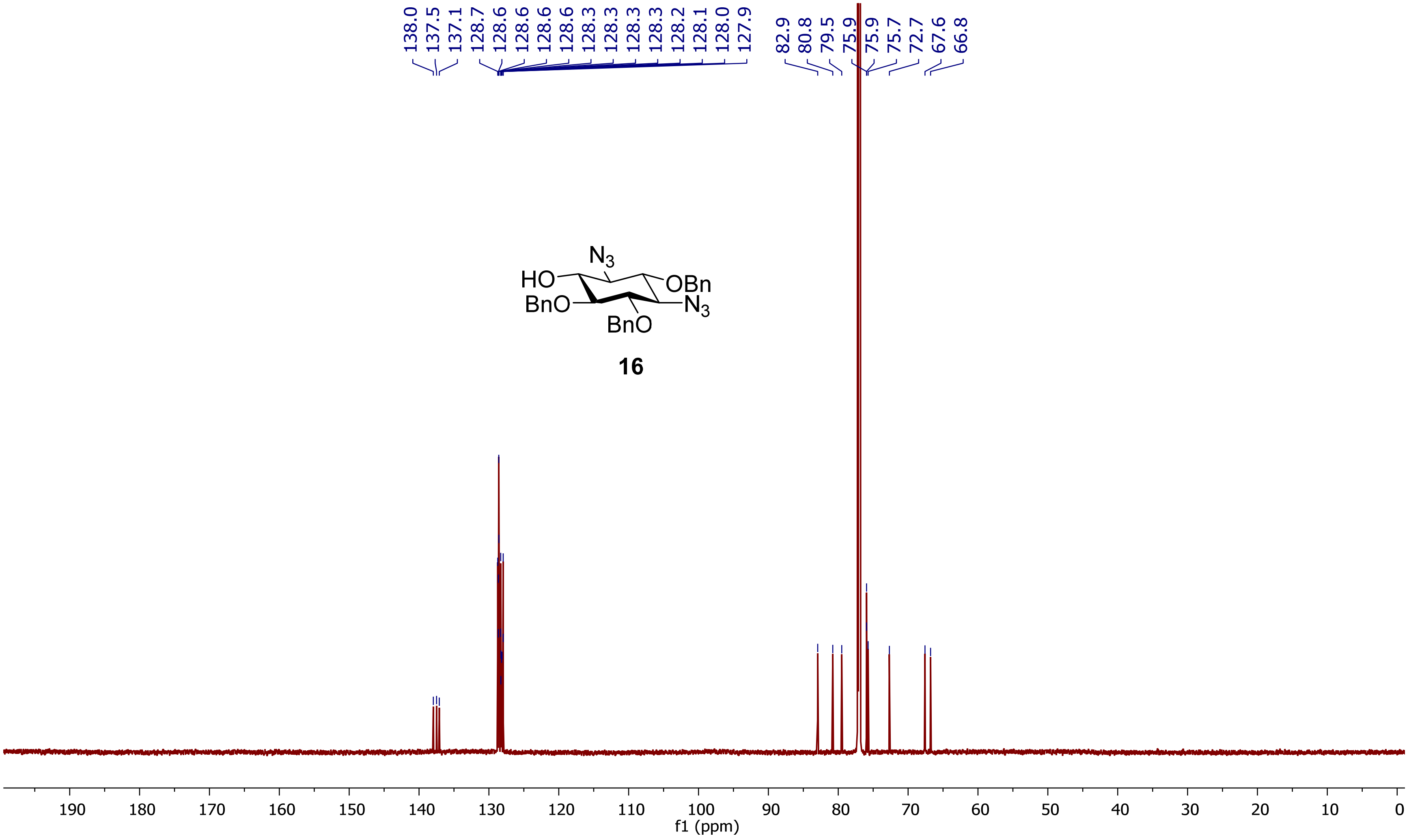

COSY NMR (900 MHz, $CDCl_3$) spectrum of 1,3-Di(deamino)-1,3-diazido-2,5,6-tri-*O*-benzyl streptamine (**16**)

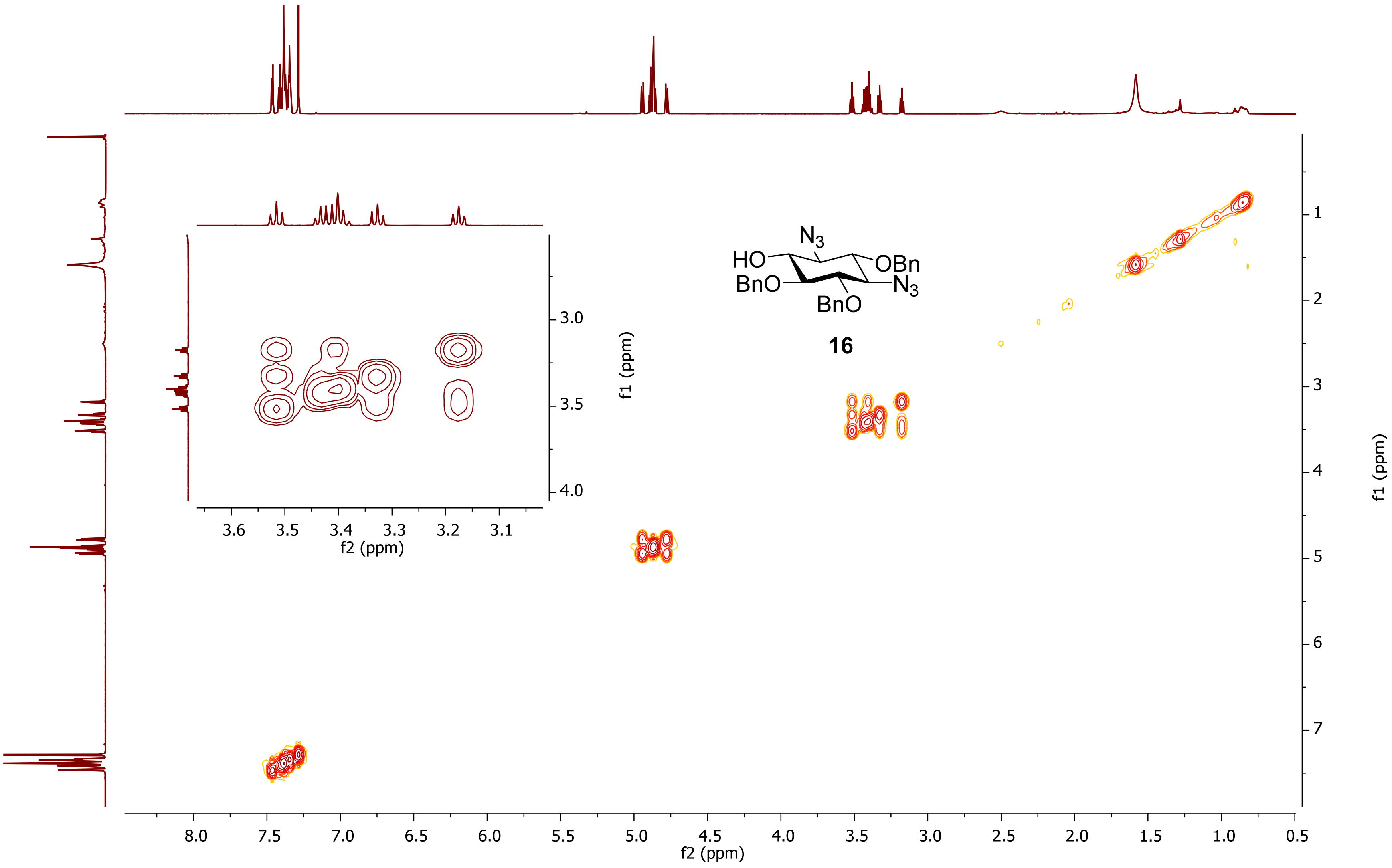

HSQC NMR (900 MHz, $CDCl_3$) spectrum of 1,3-Di(deamino)-1,3-diazido-2,5,6-tri-*O*-benzyl streptamine (**16**)

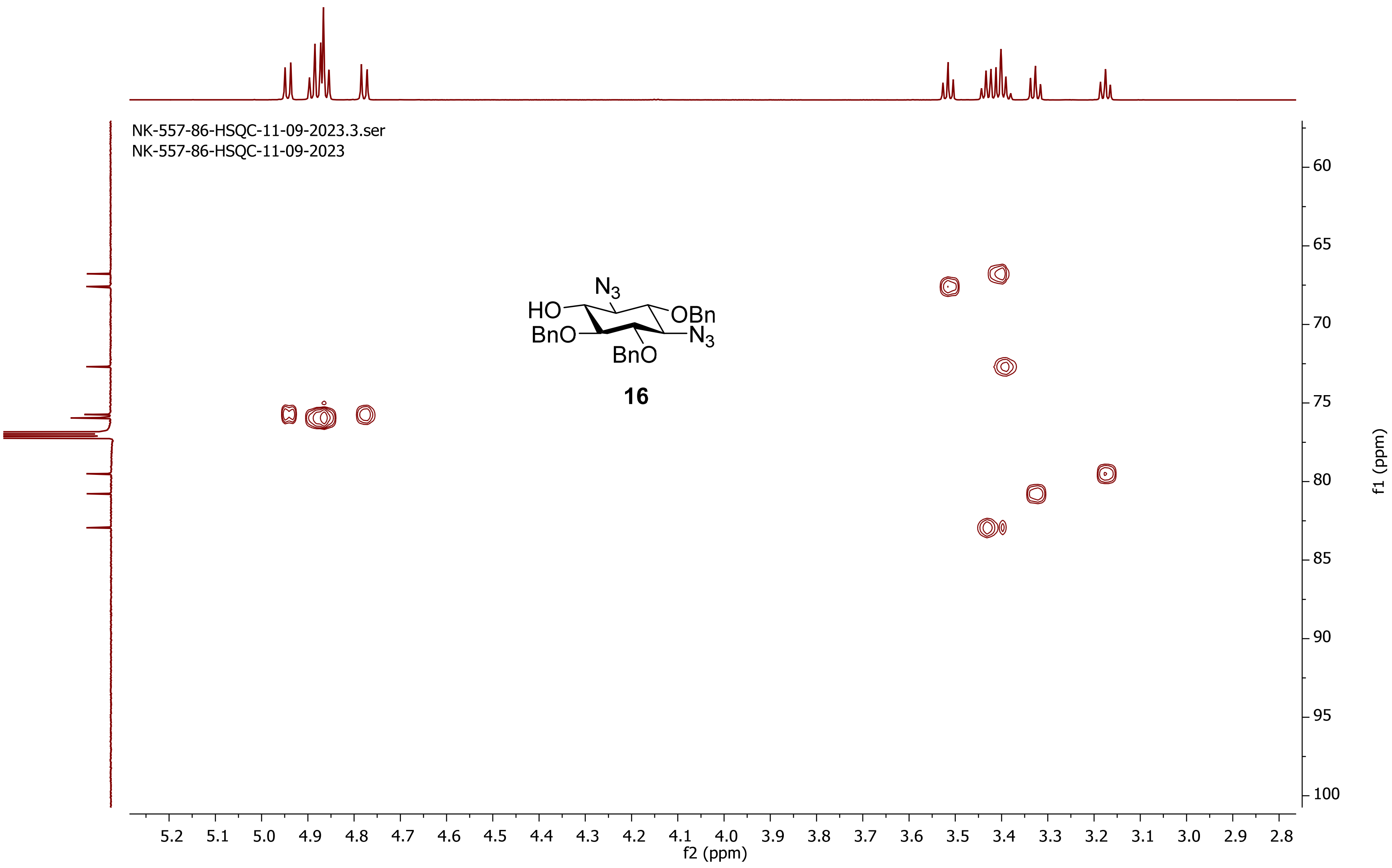

^{1}H NMR Spectra (500 MHz, Benzene-d_6) of 1,3-Di(deamino)-1,3-diazido-2,6-di-*O*-benzylstreptamine (**17**)

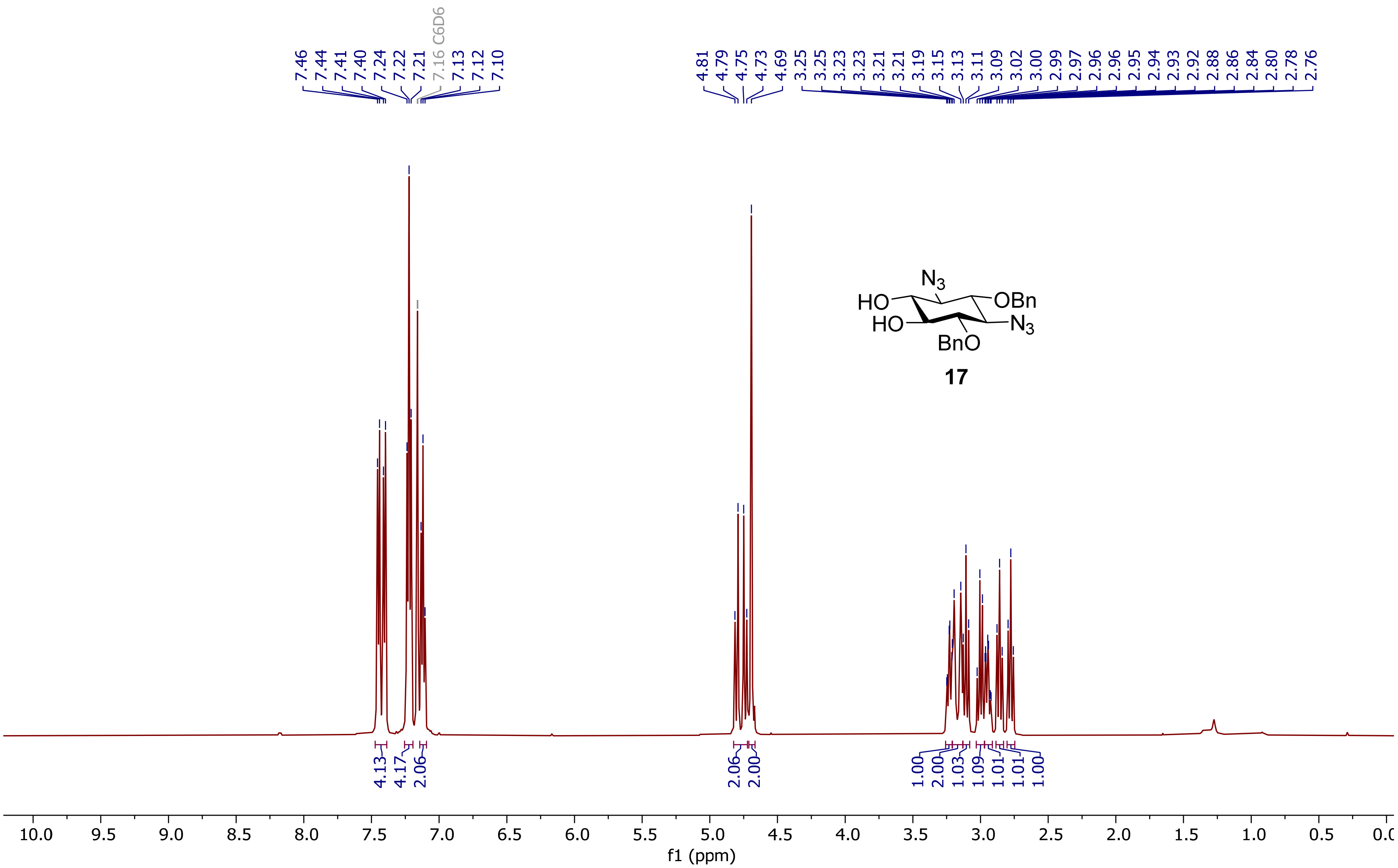

$^{13}C\{^{1}H\}$ NMR Spectra (126 MHz, Benzene-d_6) of 1,3-Di(deamino)-1,3-diazido-2,6-di-*O*-benzylstreptamine (**17**)

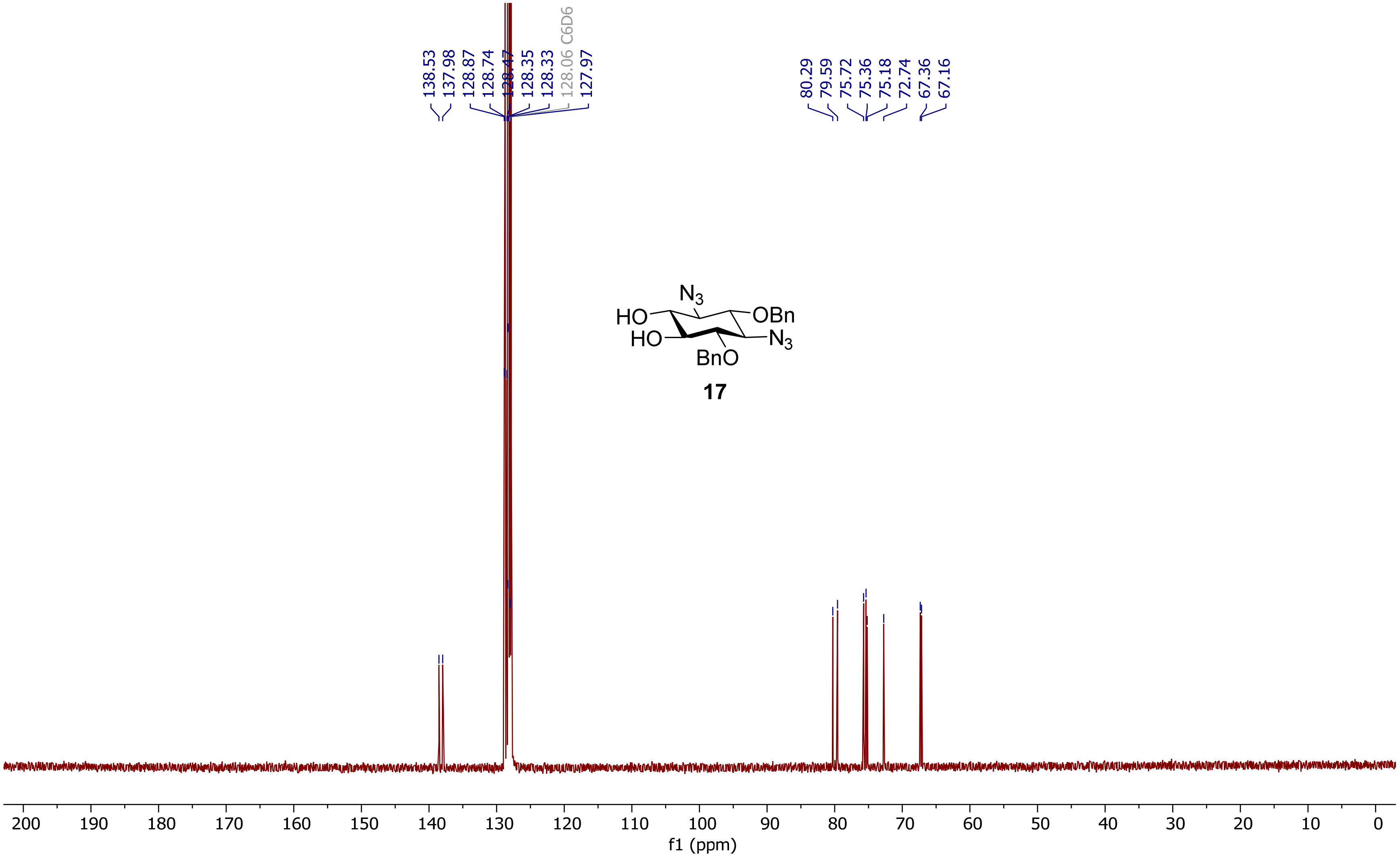

^{1}H-^{1}H COSY NMR Spectra (500 MHz, Benzene-d_6) of 1,3-Di(deamino)-1,3-diazido-2,6-di-*O*-benzylstreptamine (**17**)

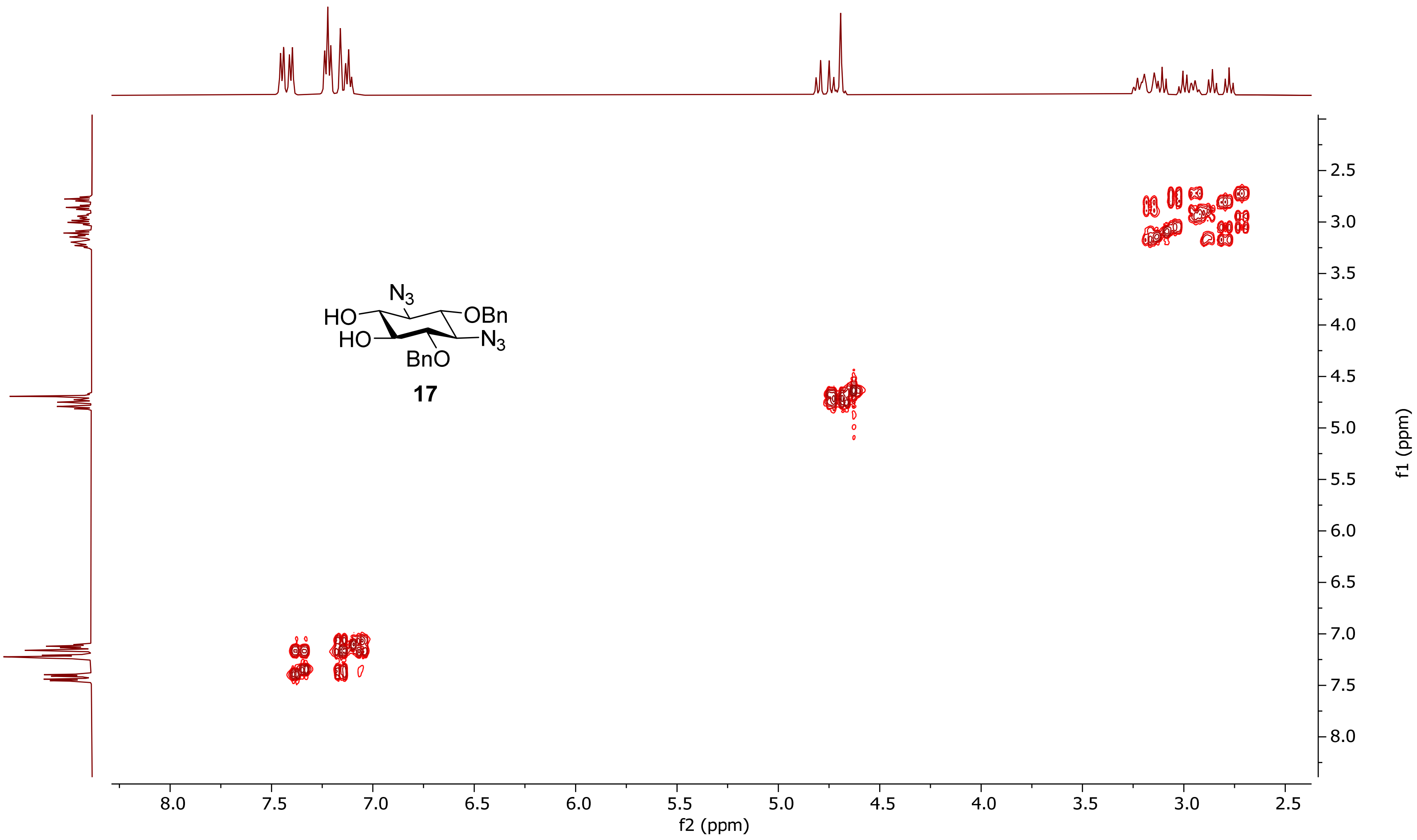

HSQC NMR Spectra (500 MHz, Benzene-d_6) of 1,3-Di(deamino)-1,3-diazido-2,6-di-*O*-benzylstreptamine (**17**)

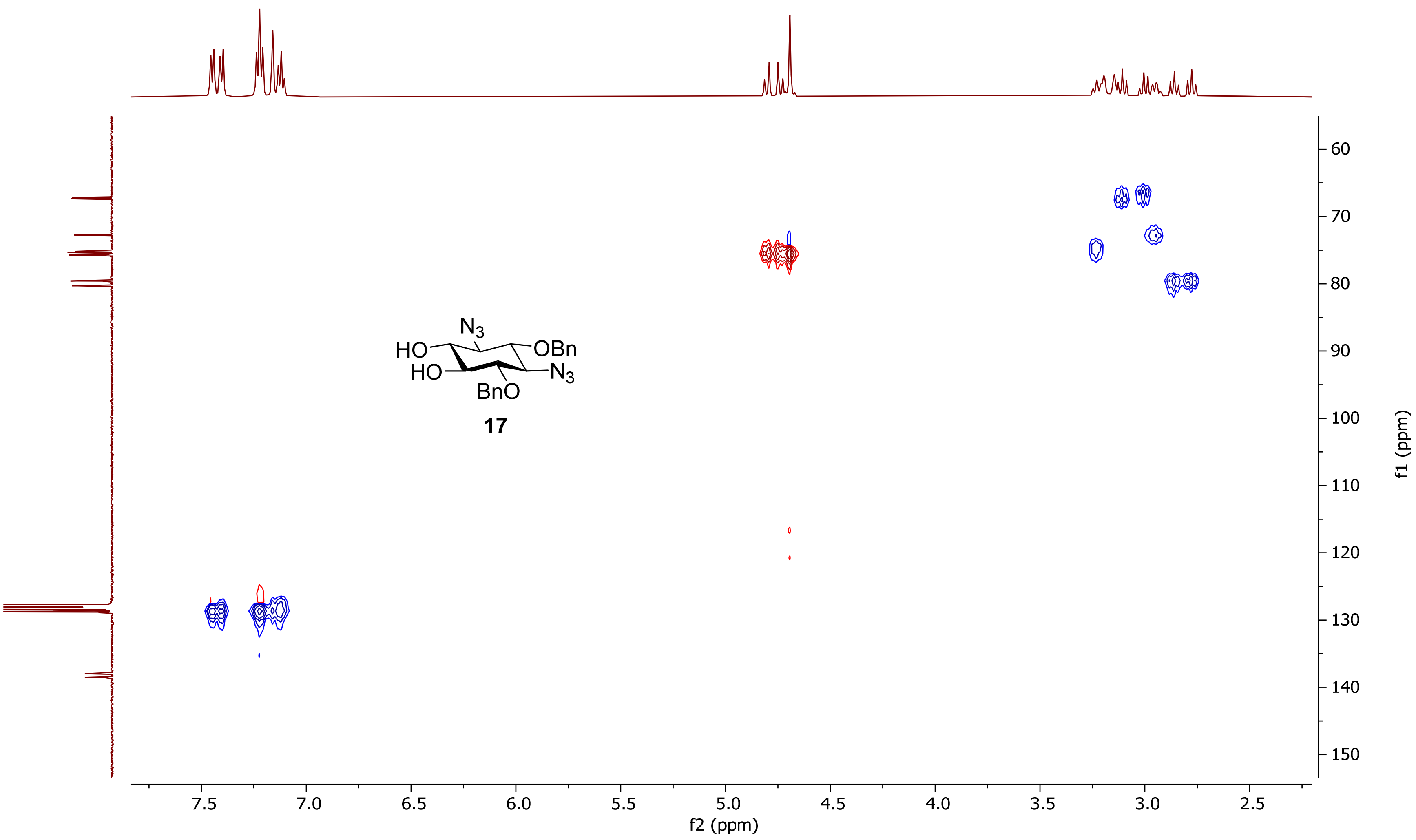

^{1}H NMR Spectra (500 MHz, Benzene-d_6) of 4,5-Di-*O*-acetyl-1,3-di(deamino)-1,3-diazido-2,6-di-*O*-benzylstreptamine (**18**)

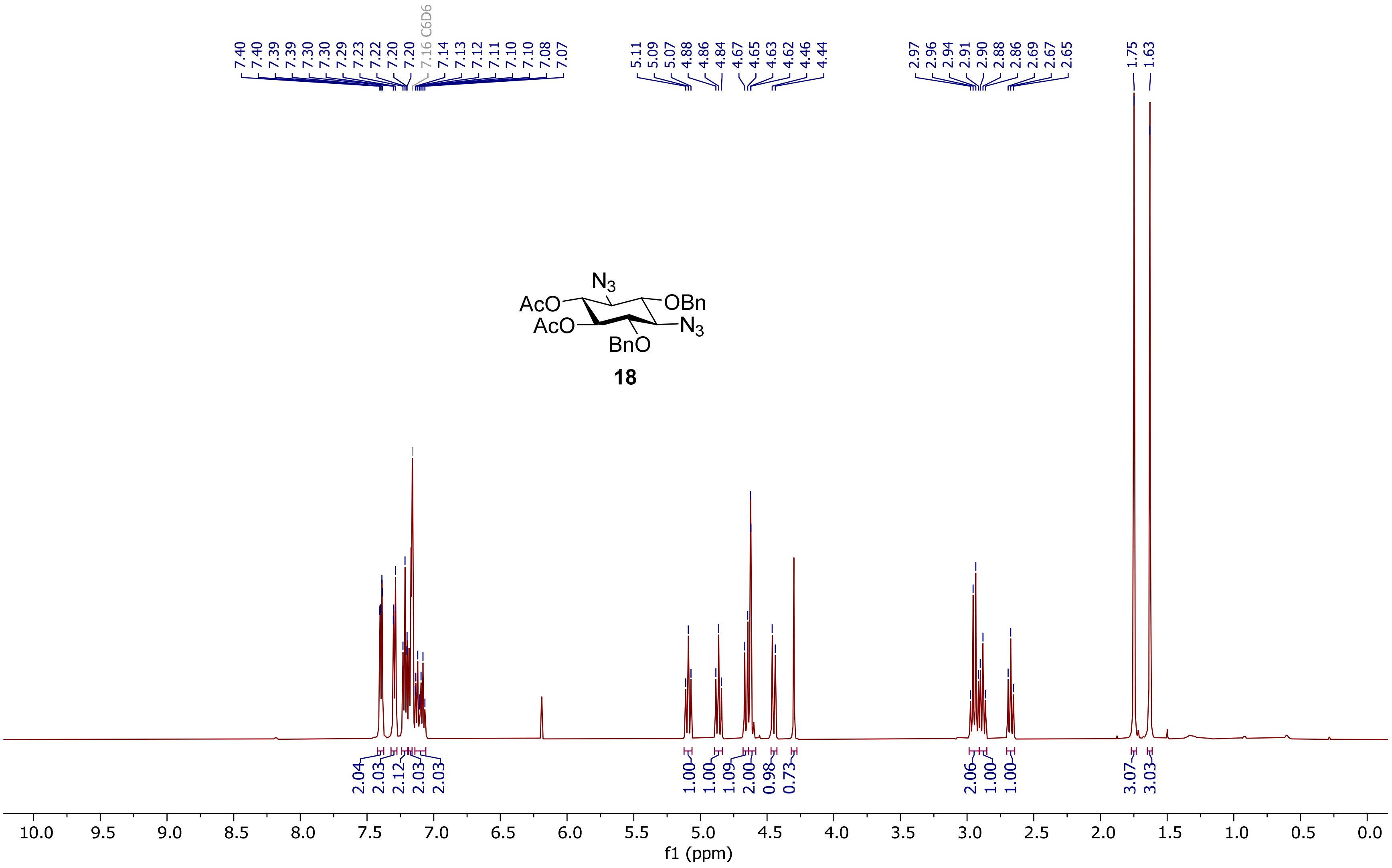

$^{13}C\{^{1}H\}$ NMR Spectra (126 MHz, Benzene-d_6) of 4,5-Di-*O*-acetyl-1,3-di(deamino)-1,3-diazido-2,6-di-*O*-benzylstreptamine (**18**)

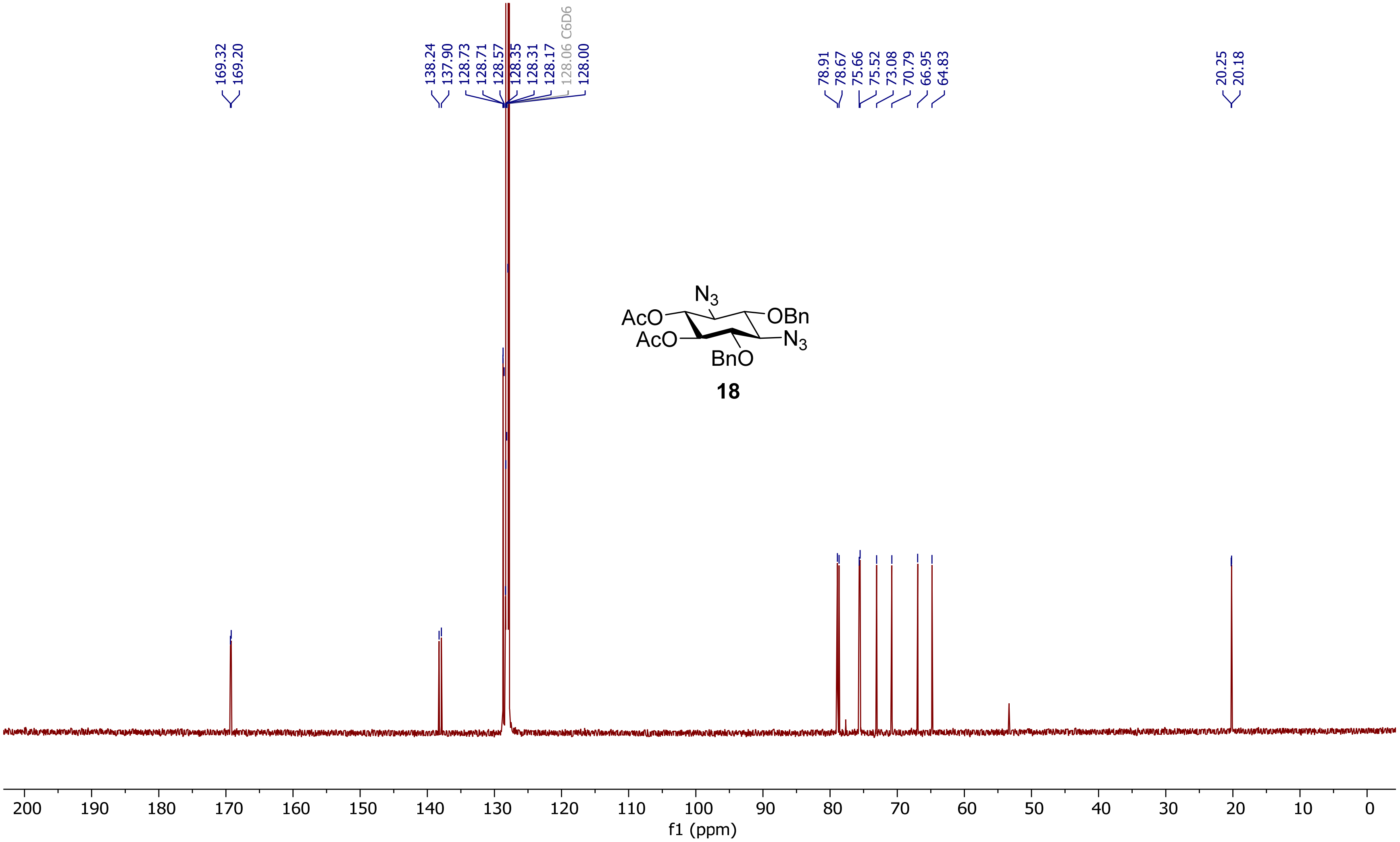

^{1}H-^{1}H COSY NMR Spectra (500 MHz, Benzene-d_6) of 4,5-Di-*O*-acetyl-1,3-di(deamino)-1,3-diazido-2,6-di-*O*-benzylstreptamine (**18**)

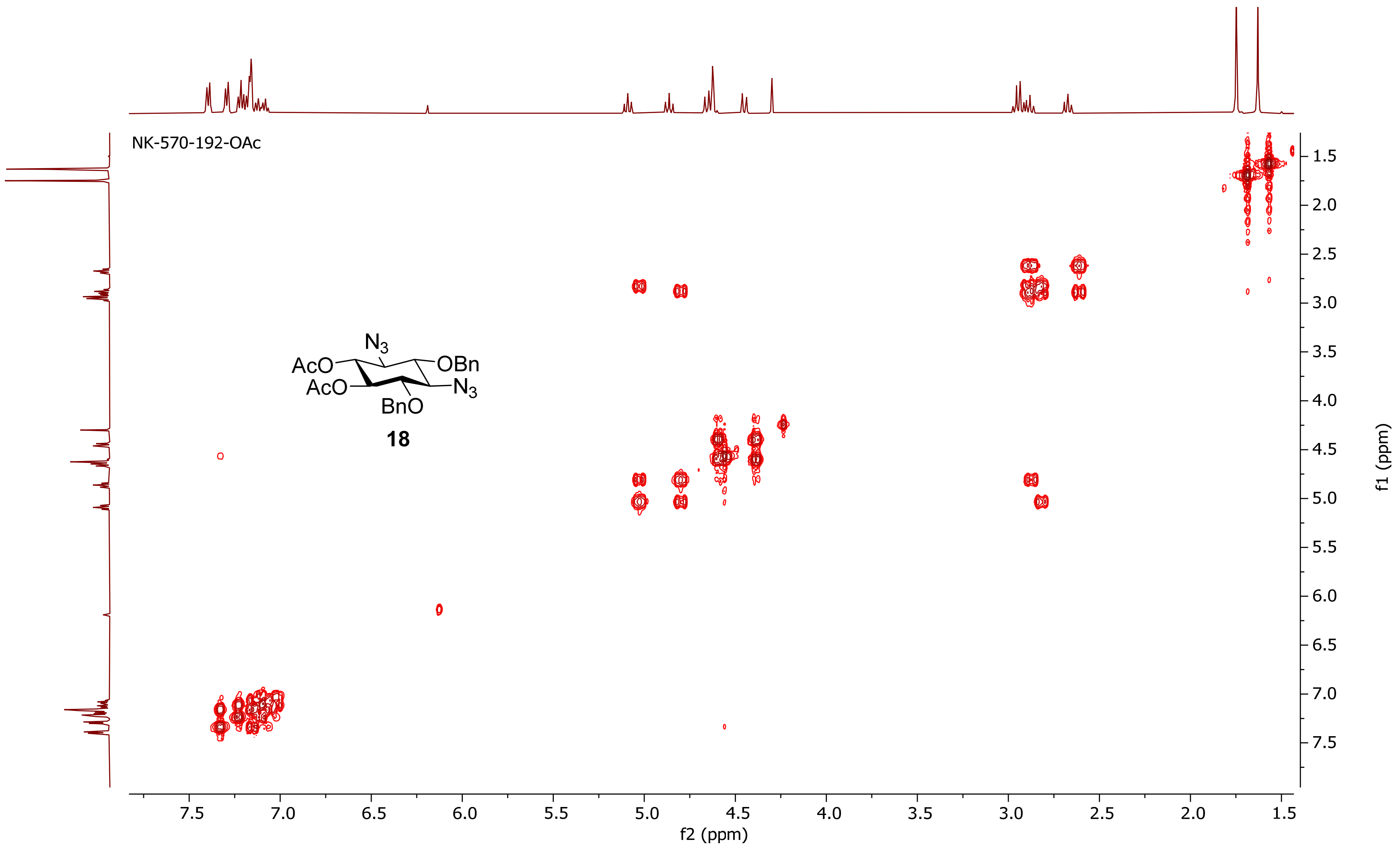

HSQC NMR Spectra (500 MHz, Benzene-d_6) of 4,5-Di-*O*-acetyl-1,3-di(deamino)-1,3-diazido-2,6-di-*O*-benzylstreptamine (**18**)

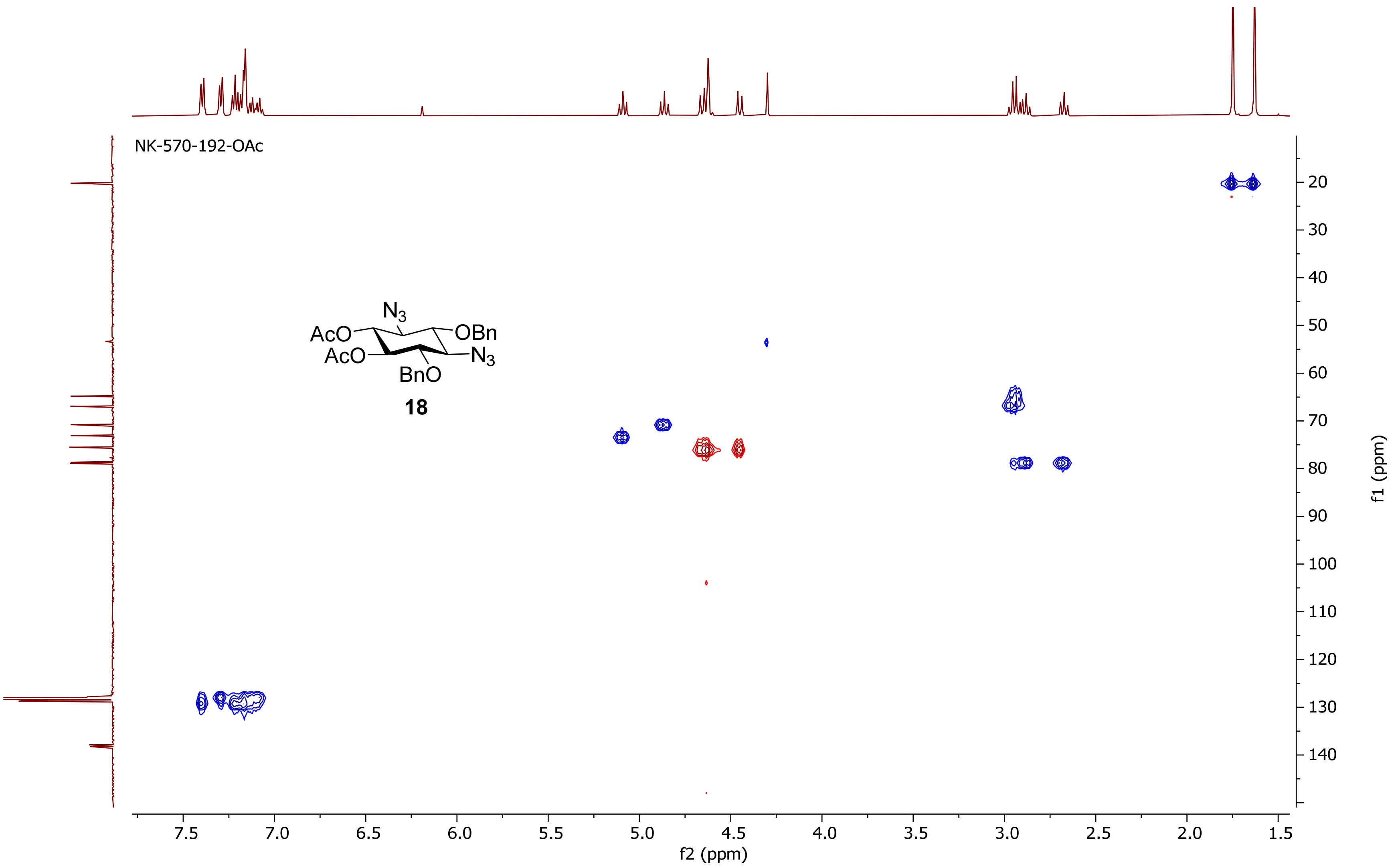

^{1}H NMR Spectra (500 MHz, $CDCl_3$) of 1,3-Di(deamino)-1,3-diazido-5,6-di-*O*-benzylstreptamine (**19**)

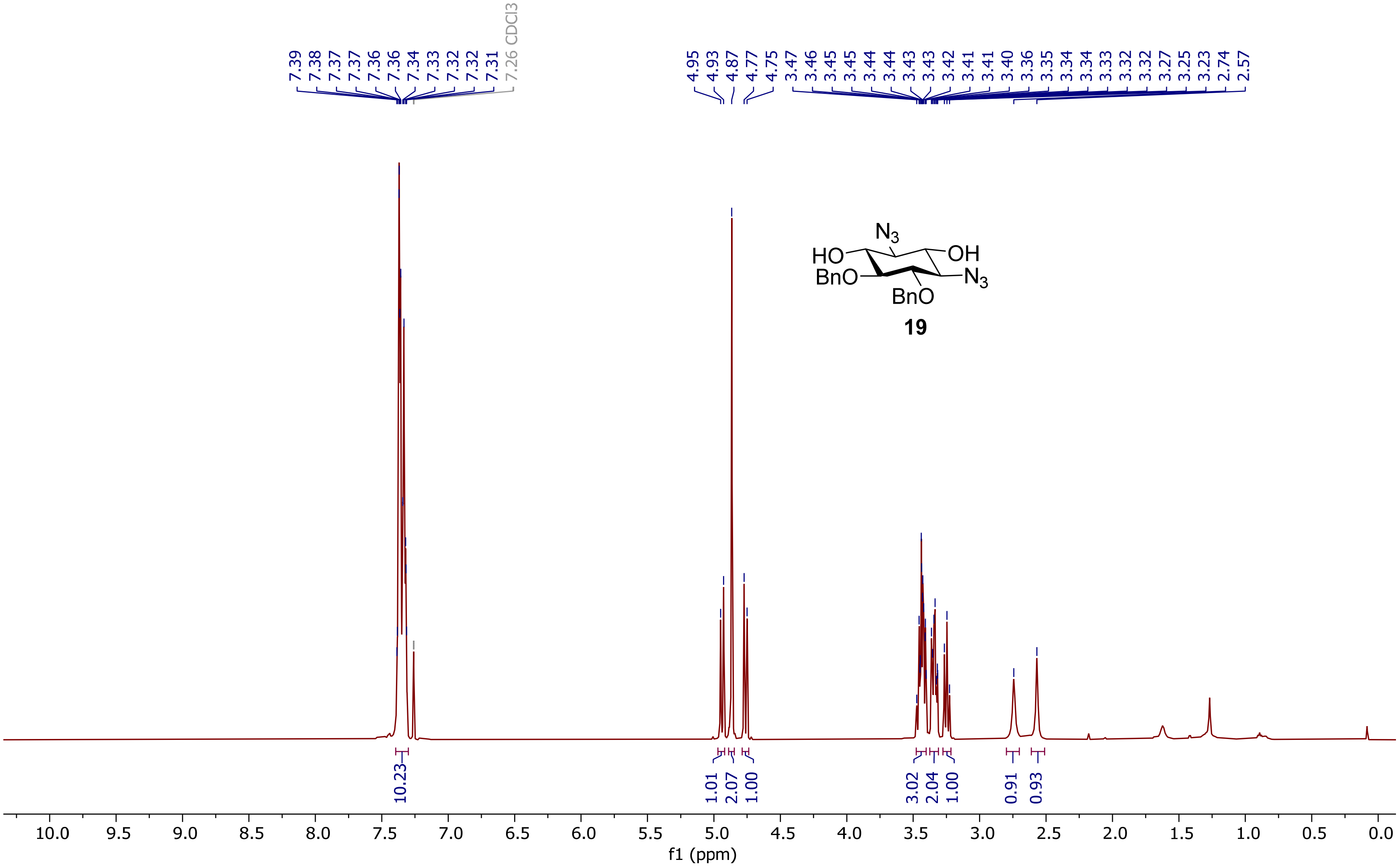

$^{13}C\{^1H\}$ NMR Spectra (126 MHz, $CDCl_3$) of 1,3-Di(deamino)-1,3-diazido-5,6-di-*O*-benzylstreptamine (**19**)

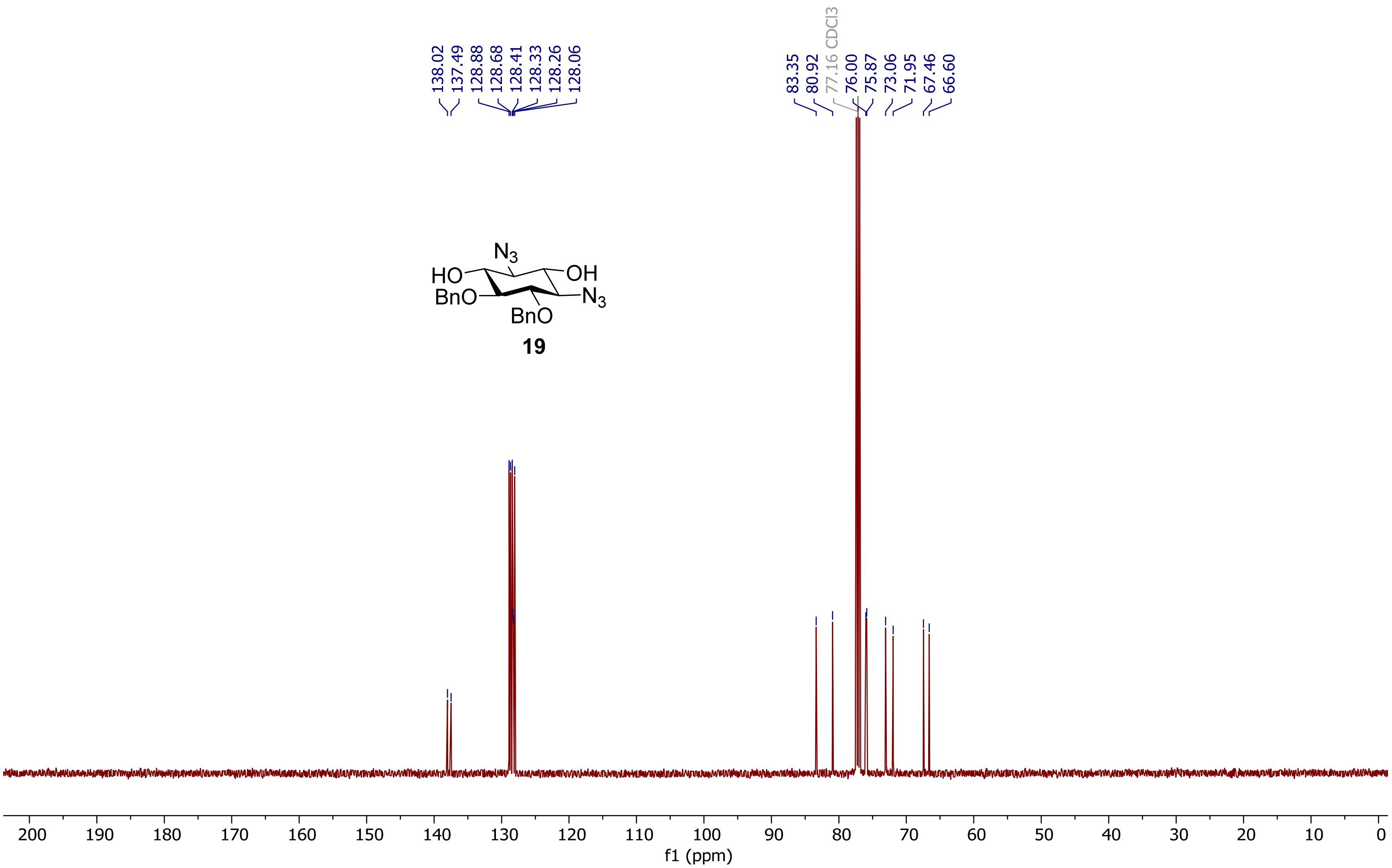

^{1}H-^{1}H COSY NMR Spectra (500 MHz, $CDCl_3$) of 1,3-Di(deamino)-1,3-diazido-5,6-di-*O*-benzylstreptamine (**19**)

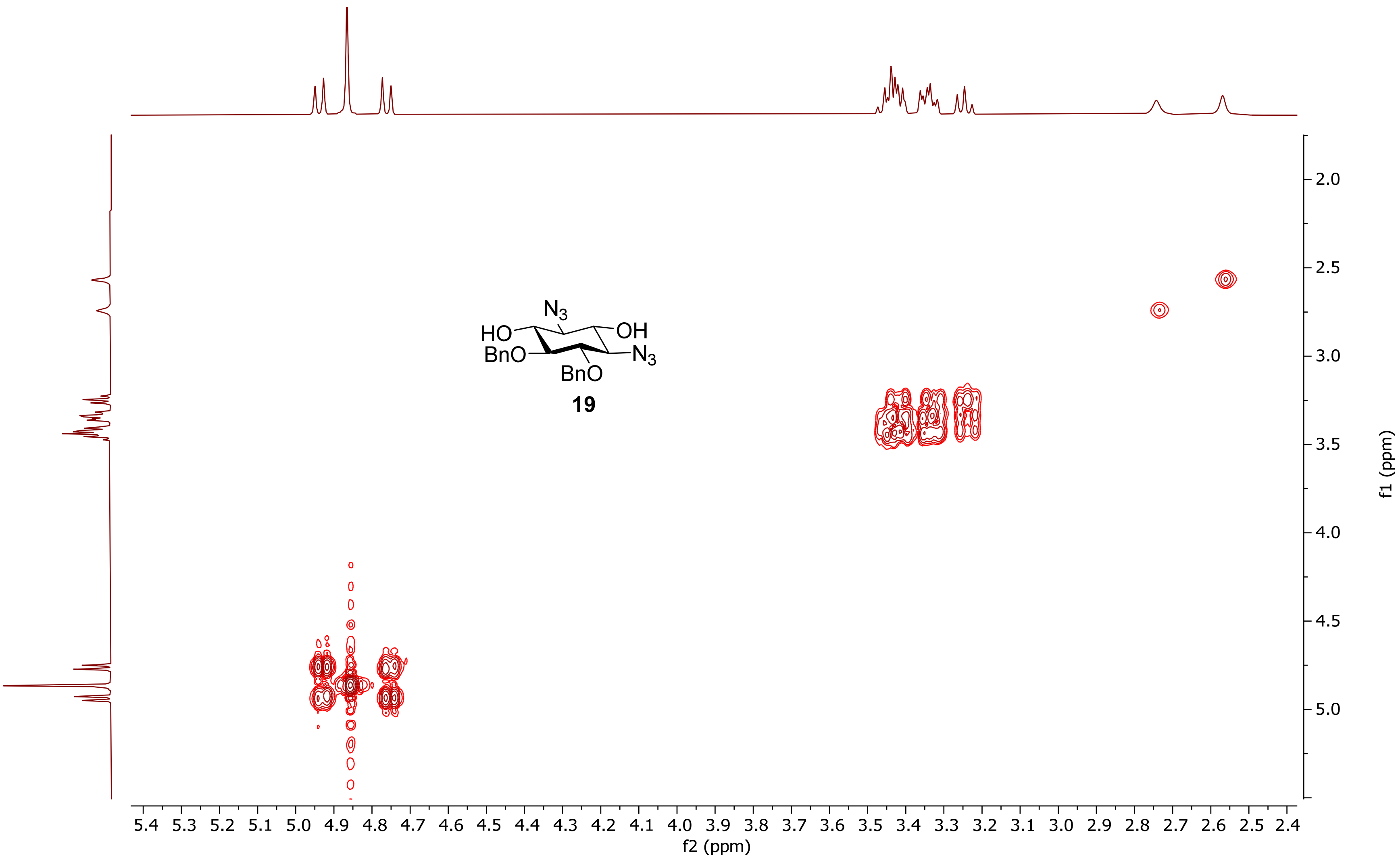

HSQC NMR Spectra (500 MHz, $CDCl_3$) of 1,3-Di(deamino)-1,3-diazido-5,6-di-*O*-benzylstreptamine (**19**)

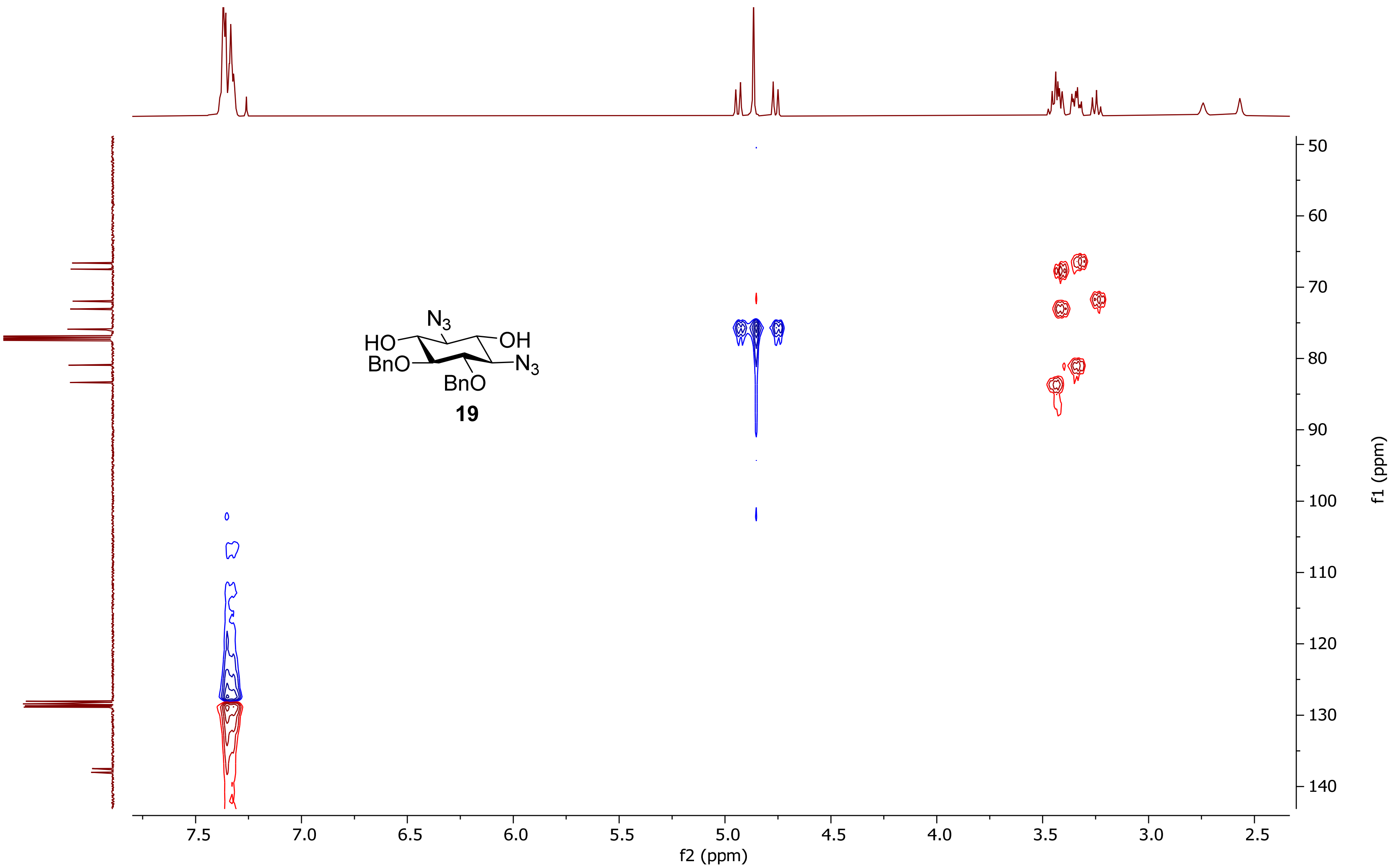

^{1}H NMR Spectra (500 MHz, Benzene-d_6) of 1,3-Di(deamino)-1,3-diazido-2,5-di-*O*-benzylstreptamine (**20**)

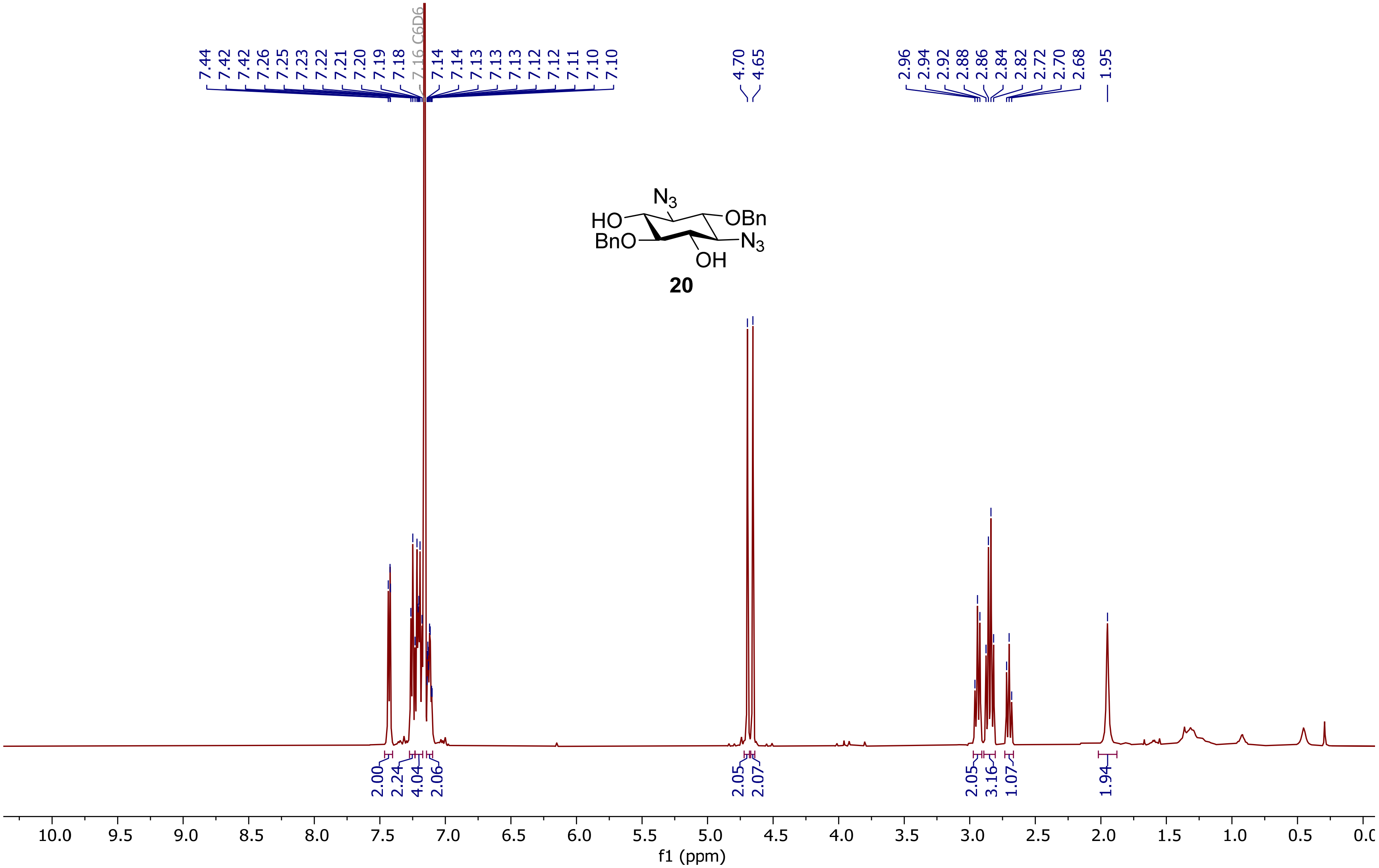

$^{13}C\{^1H\}$ NMR Spectra (126 MHz, Benzene-d_6) of 1,3-Di(deamino)-1,3-diazido-2,5-di-*O*-benzylstreptamine (**20**)

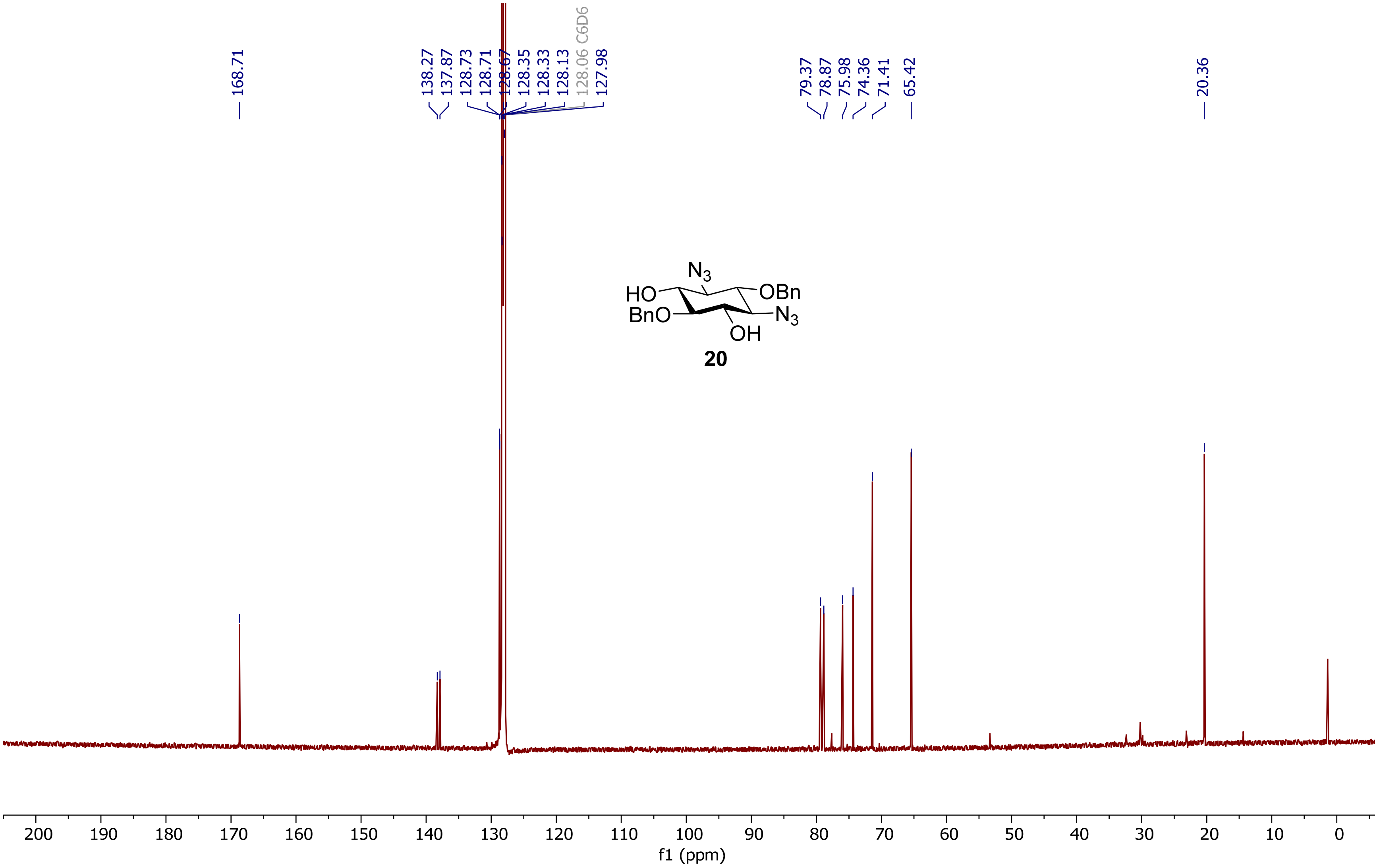

^{1}H-^{1}H COSY NMR Spectra (500 MHz, Benzene-d_6) of 1,3-Di(deamino)-1,3-diazido-2,5-di-*O*-benzylstreptamine (**20**)

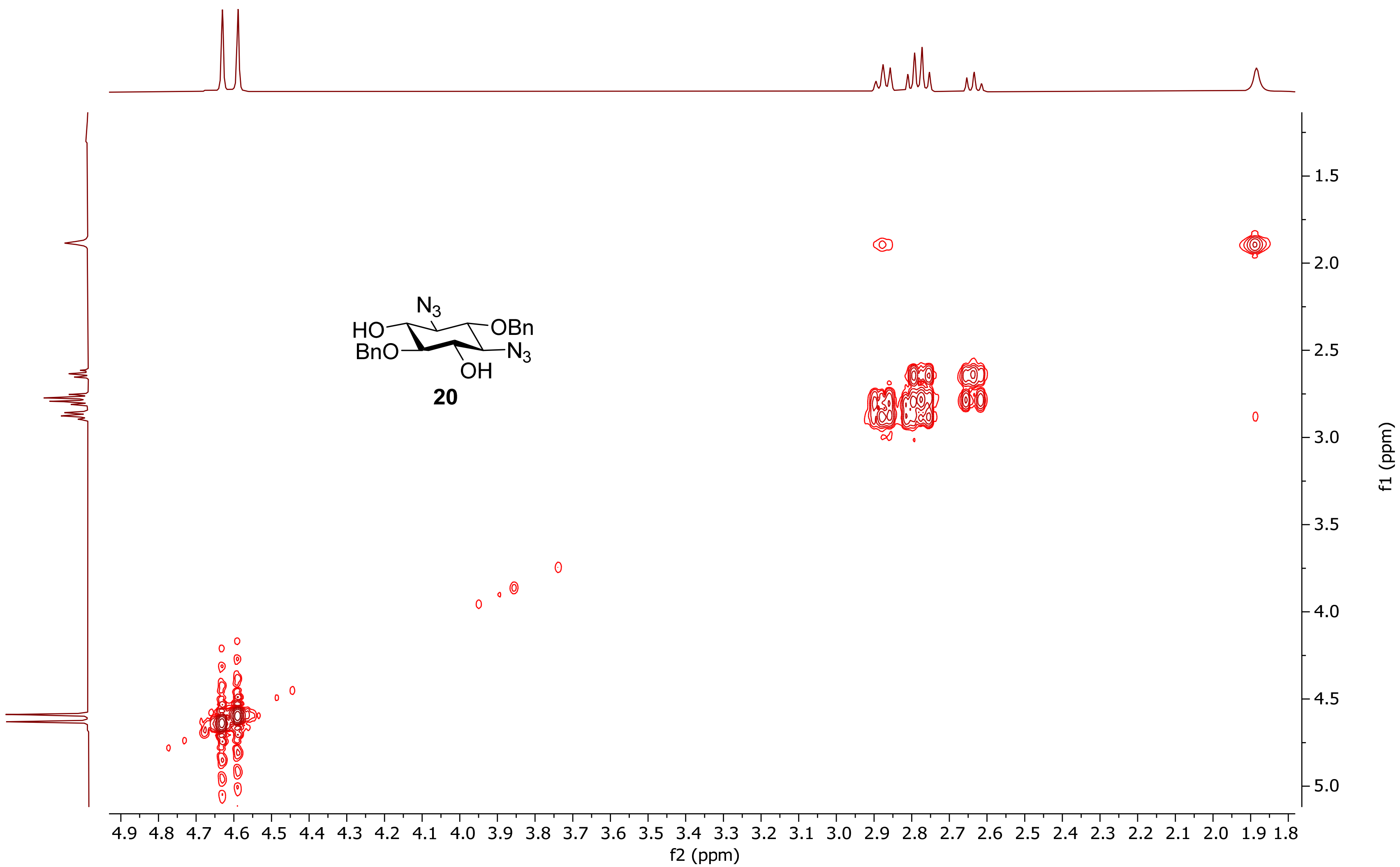

HSQC NMR Spectra (500 MHz, Benzene-d_6) of 1,3-Di(deamino)-1,3-diazido-2,5-di-*O*-benzylstreptamine (**20**)

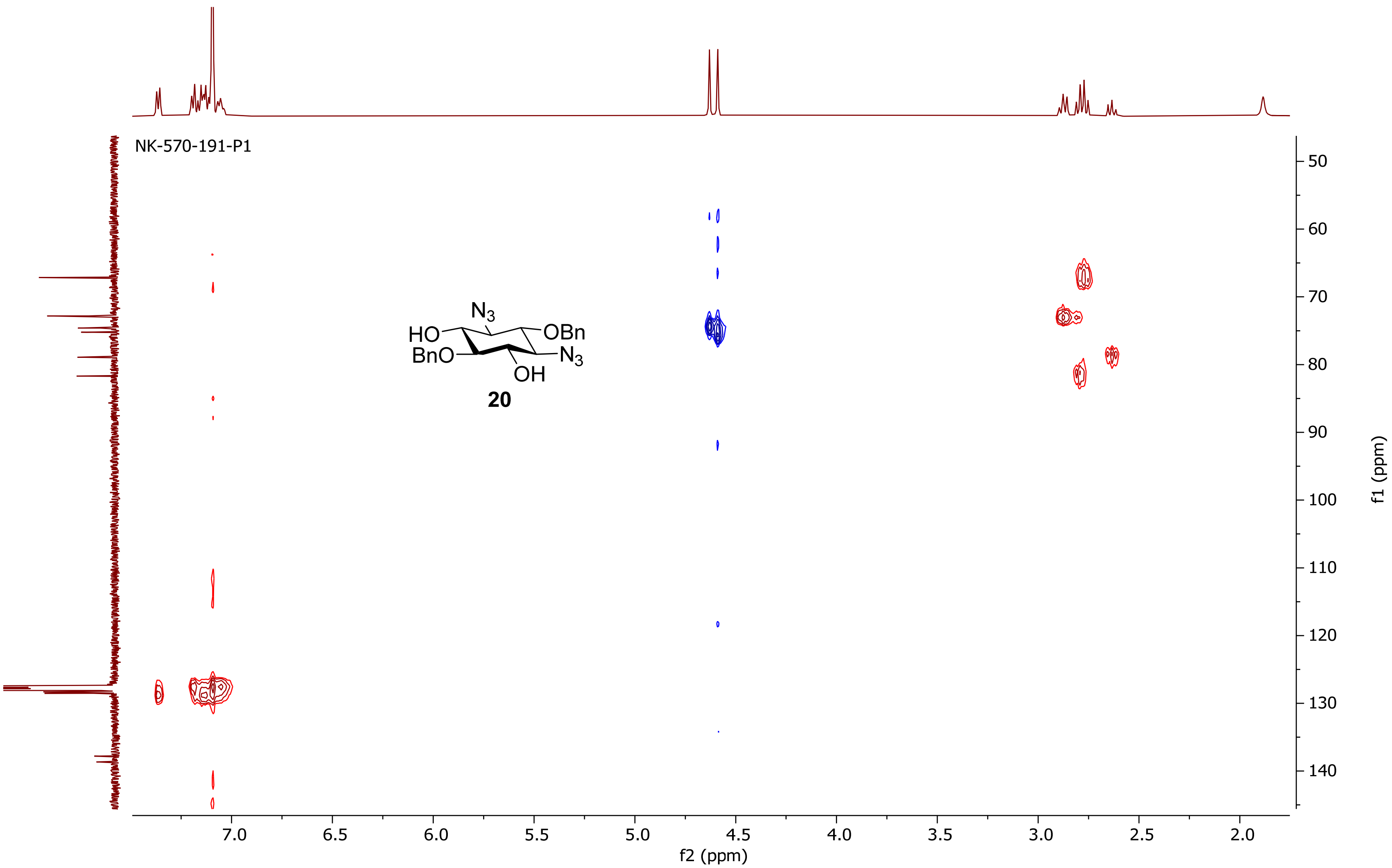

^{1}H NMR Spectra (500 MHz, Benzene-d_6) of 2,4-Di-*O*-acetyl-1,3-di(deamino)-1,3-diazido-5,6-di-*O*-benzylstreptamine (**21**)

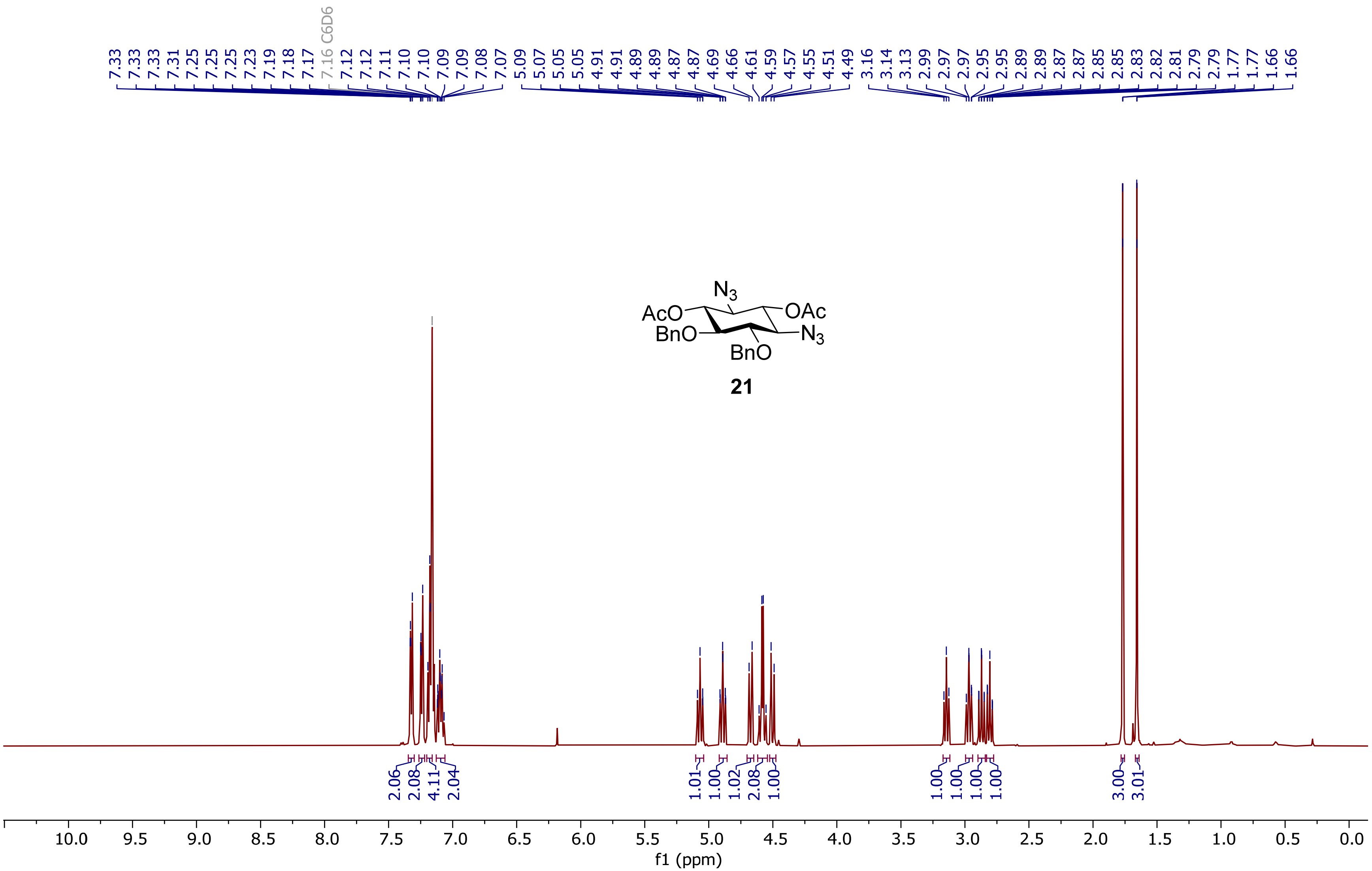

$^{13}C\{^1H\}$ NMR Spectra (126 MHz, Benzene-d_6) of 2,4-Di-*O*-acetyl-1,3-di(deamino)-1,3-diazido-5,6-di-*O*-benzylstreptamine (**21**)

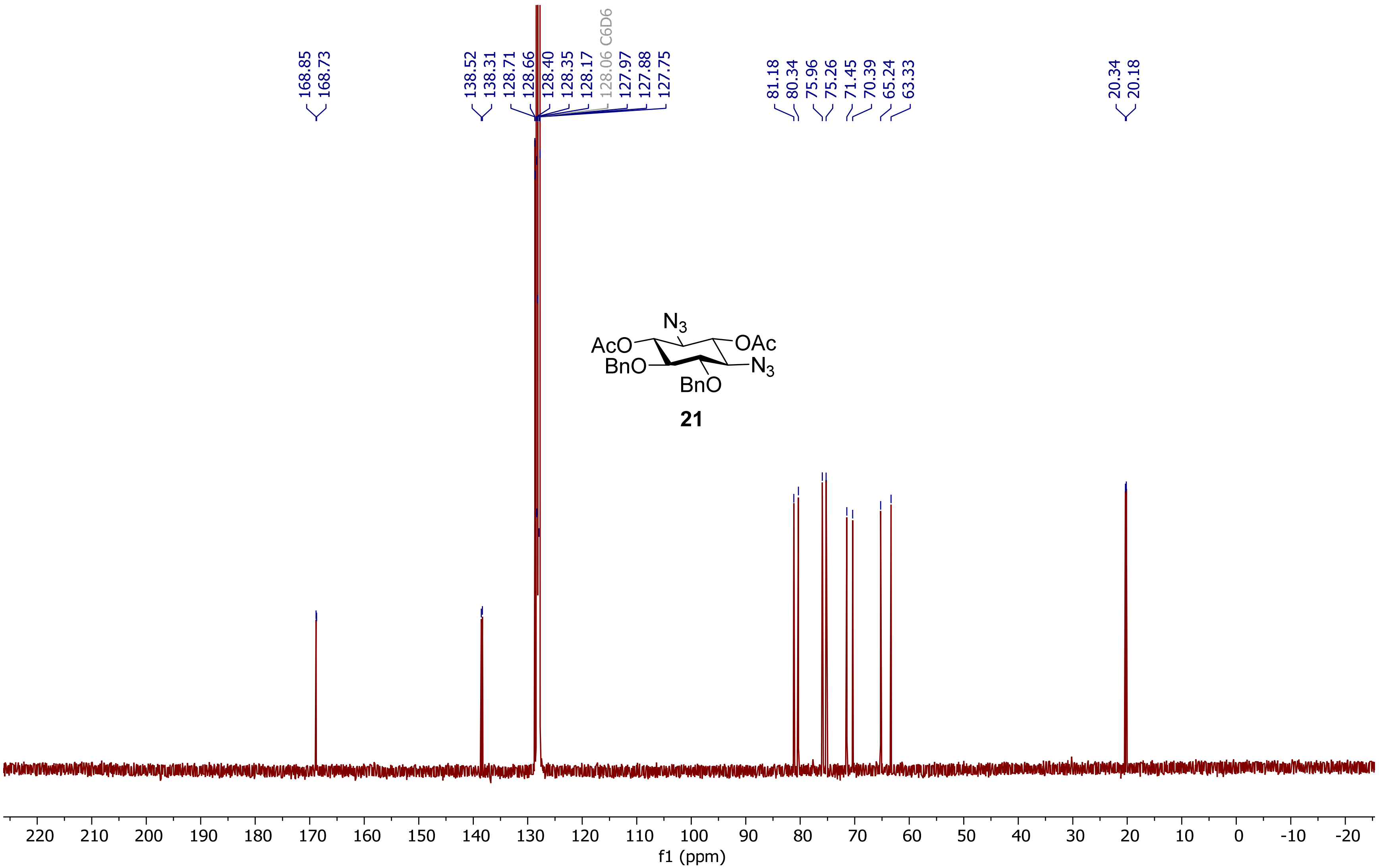

21

^{1}H-^{1}H COSY NMR Spectra (500 MHz, Benzene-d_6) of 2,4-Di-*O*-acetyl-1,3-di(deamino)-1,3-diazido-5,6-di-*O*-benzylstreptamine (**21**)

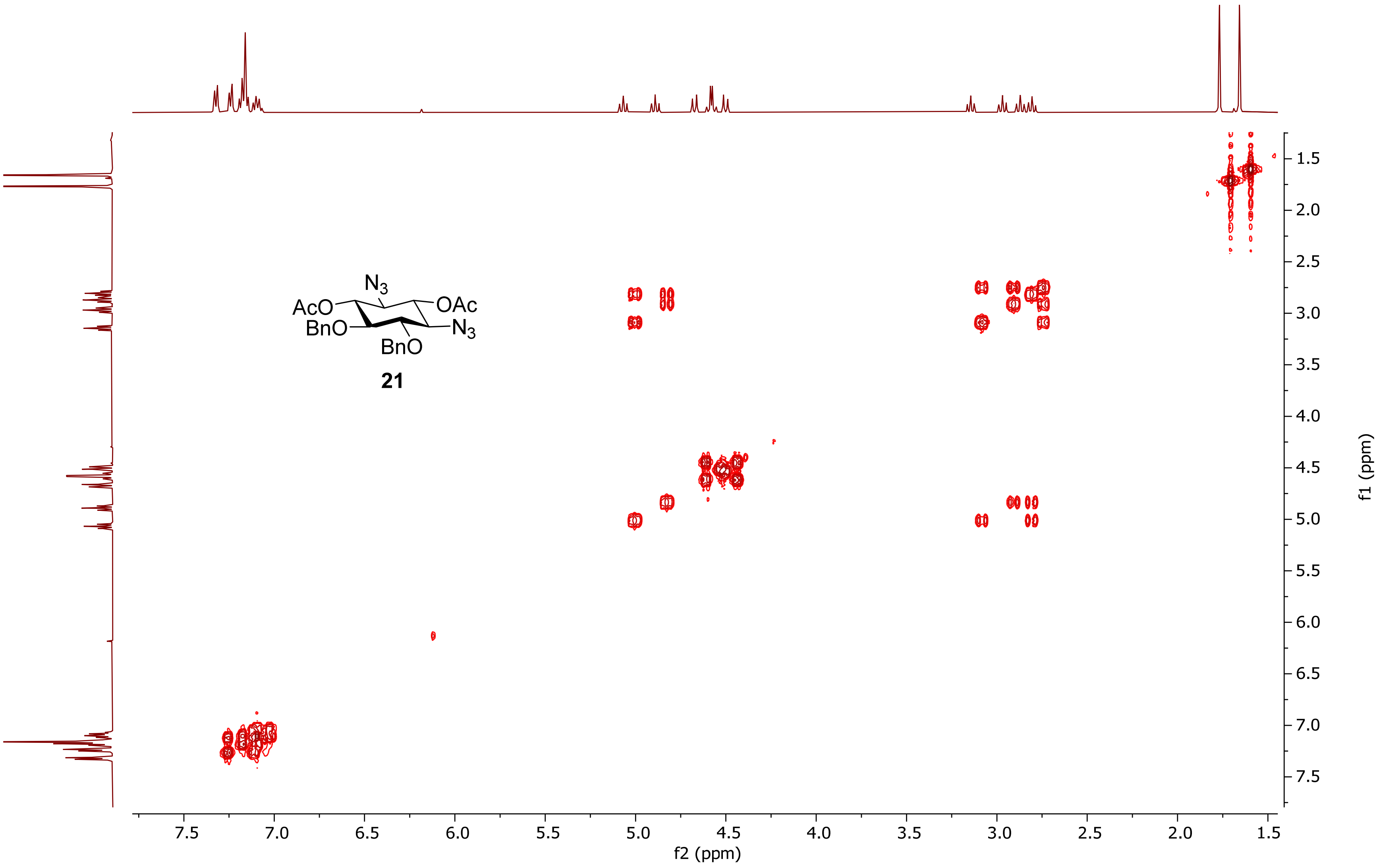

HSQC NMR Spectra (500 MHz, Benzene-d_6) of 2,4-Di-*O*-acetyl-1,3-di(deamino)-1,3-diazido-5,6-di-*O*-benzylstreptamine (**21**)

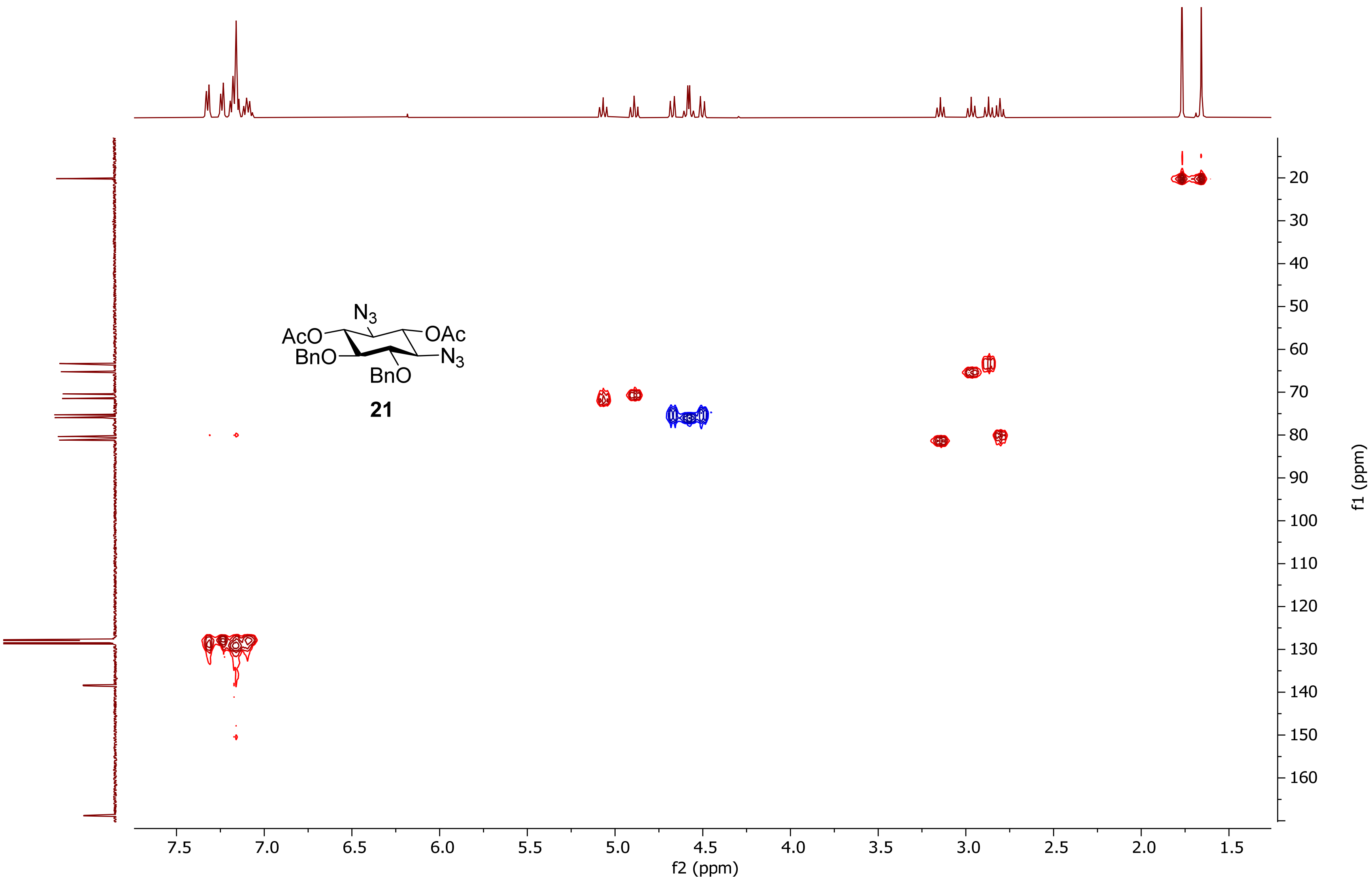

^{1}H NMR Spectra (500 MHz, Benzene-d_6) of 4,6-Di-*O*-acetyl-1,3-di(deamino)-1,3-diazido-2,5-di-*O*-benzylstreptamine (**22**)

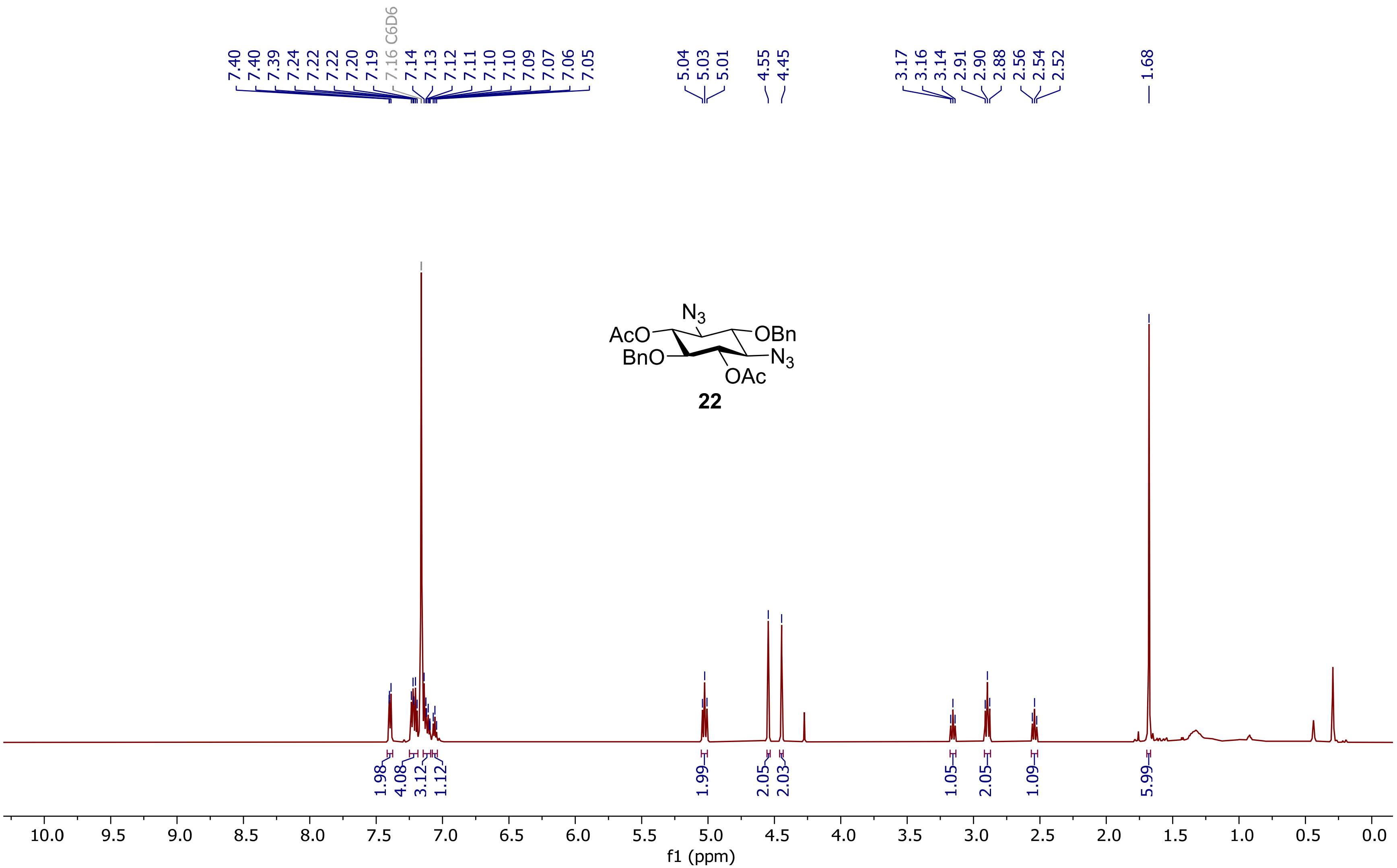

$^{13}C\{^1H\}$ NMR Spectra (500 MHz, Benzene-d_6) of 4,6-Di-*O*-acetyl-1,3-di(deamino)-1,3-diazido-2,5-di-*O*-benzylstreptamine (**22**)

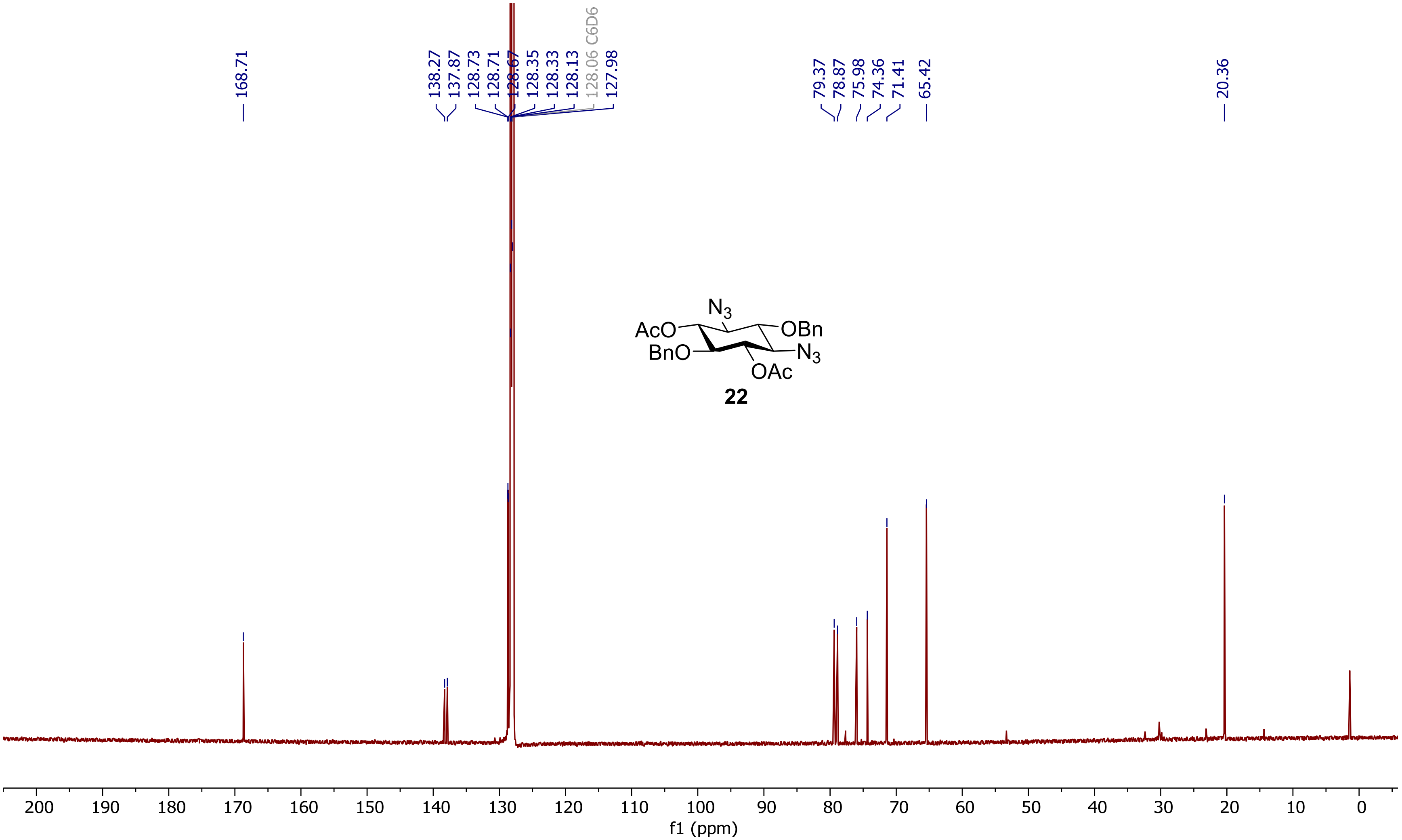

^{1}H-^{1}H COSY NMR Spectra (126 MHz, Benzene-d_6) of 4,6-Di-*O*-acetyl-1,3-di(deamino)-1,3-diazido-2,5-di-*O*-benzylstreptamine (**22**)

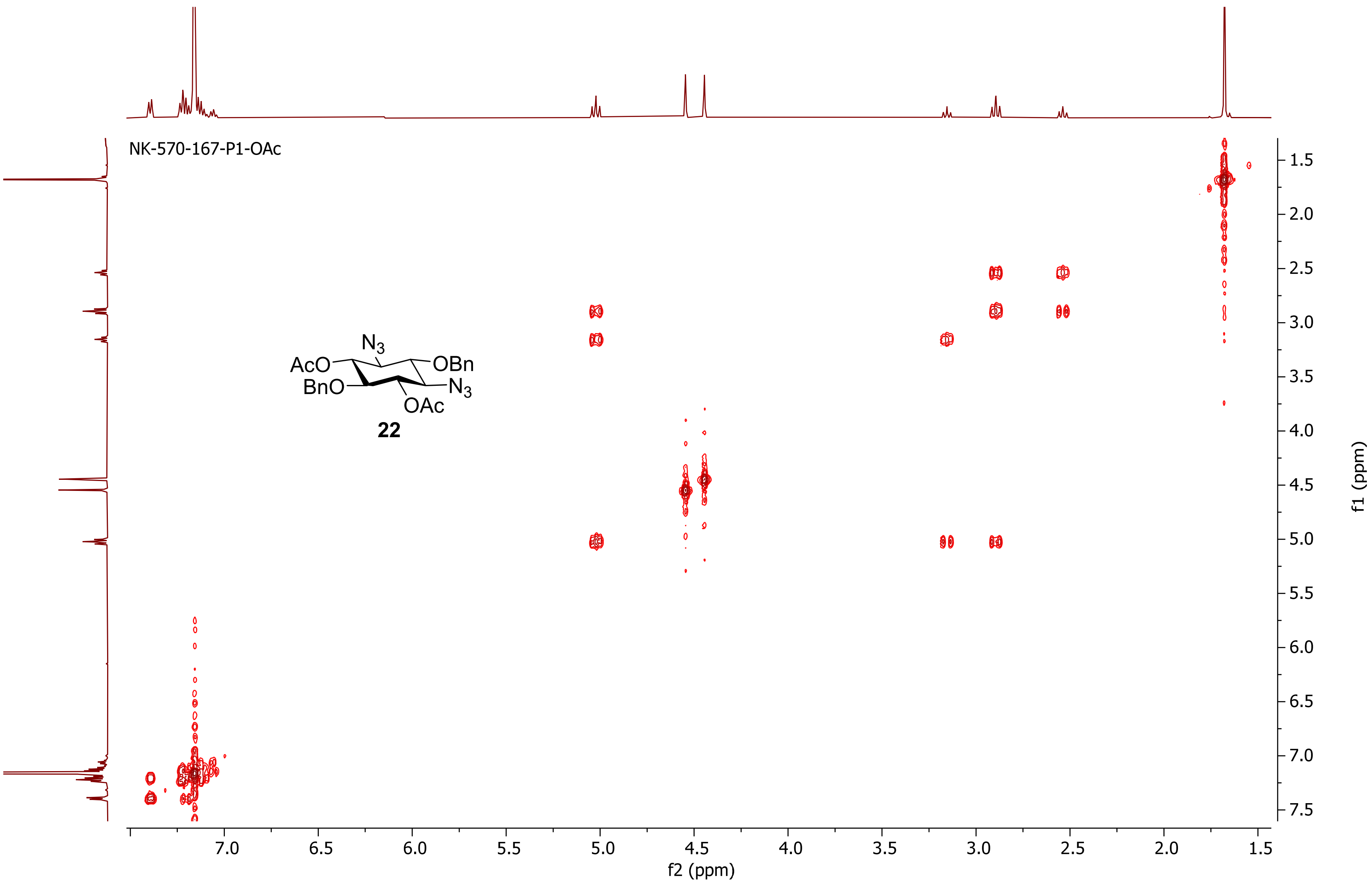

HSQC NMR Spectra (500 MHz, Benzene-d_6) of 4,6-Di-*O*-acetyl-1,3-di(deamino)-1,3-diazido-2,5-di-*O*-benzylstreptamine (**22**)

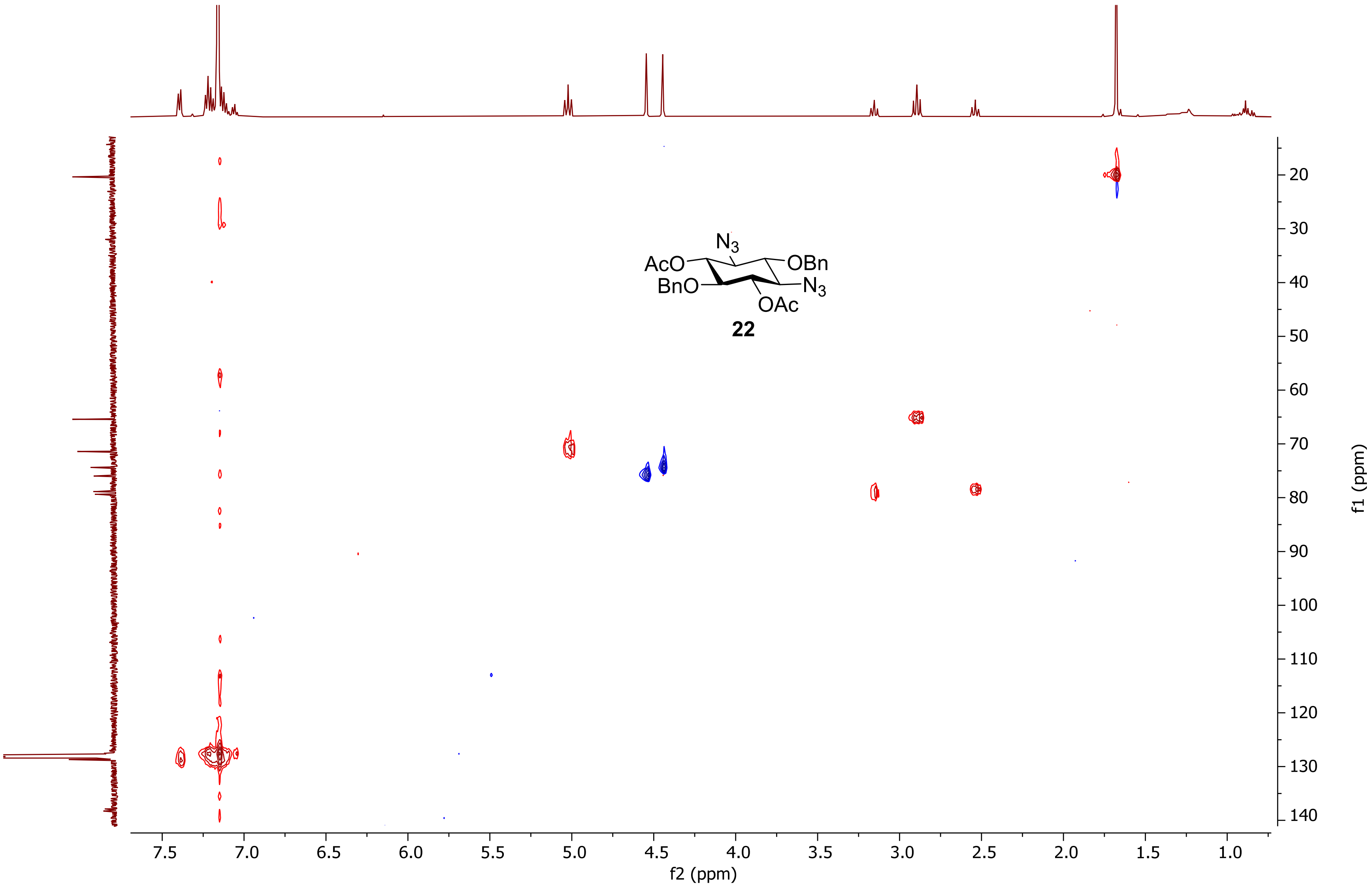

^{1}H NMR Spectra (500 MHz, Benzene-d_6) of 1,3-Di(deamino)-1-azido-3-*N*-tert-butylcarboxy-2,5,6-tri-*O*-benzyl-3,4-oxazolidino-streptamine (**23**)

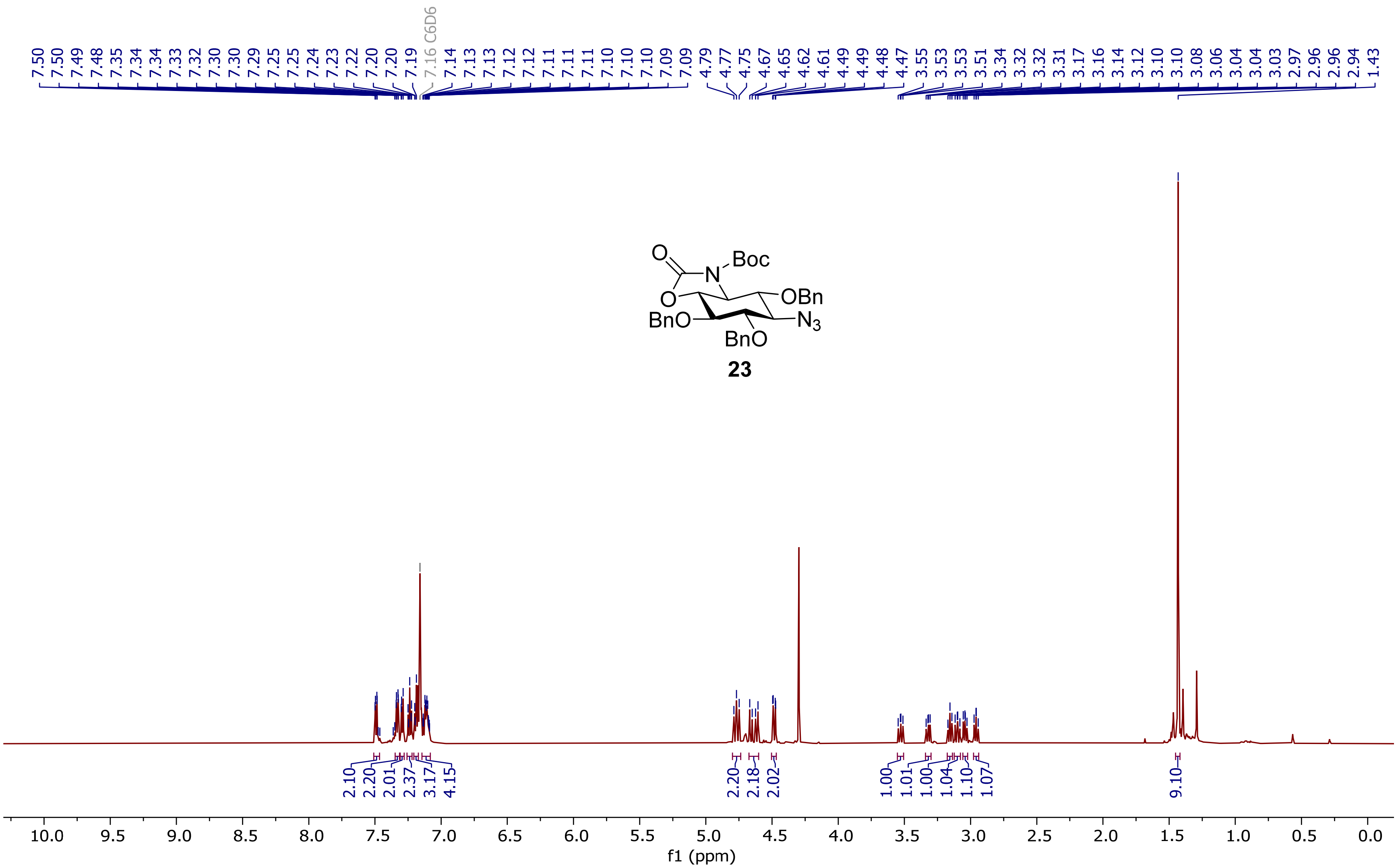

$^{13}C\{^{1}H\}$ NMR Spectra (126 MHz, Benzene-d_6) of 1,3-Di(deamino)-1-azido-3-*N*-tert-butylcarboxy-2,5,6-tri-*O*-benzyl-3,4-oxazolidino-streptamine (**23**)

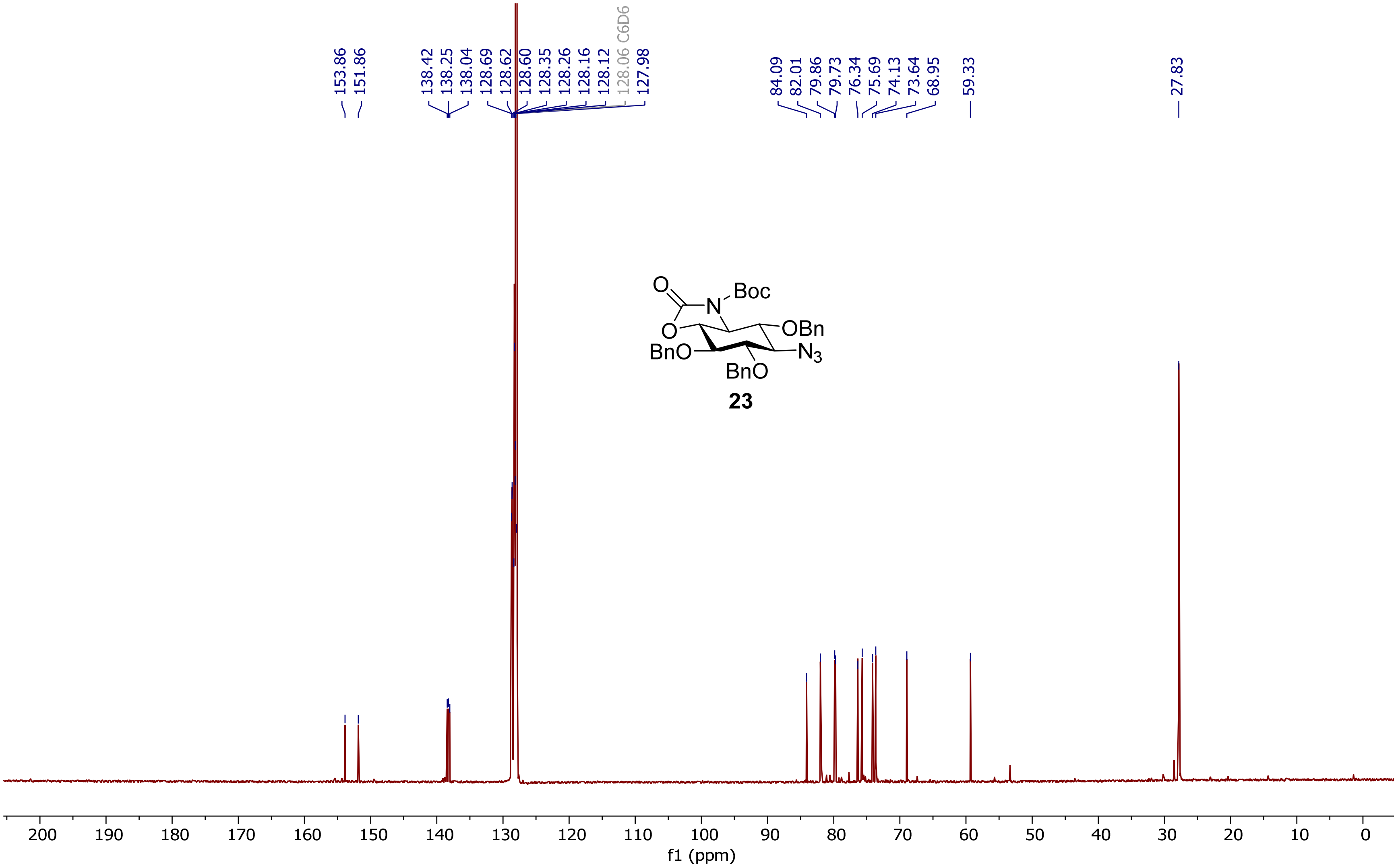

^{1}H-^{1}H COSY NMR Spectra (500 MHz, Benzene-d_6) of 1,3-Di(deamino)-1-azido-3-*N*-tert-butylcarboxy-2,5,6-tri-*O*-benzyl-3,4-oxazolidino-streptamine (**23**)

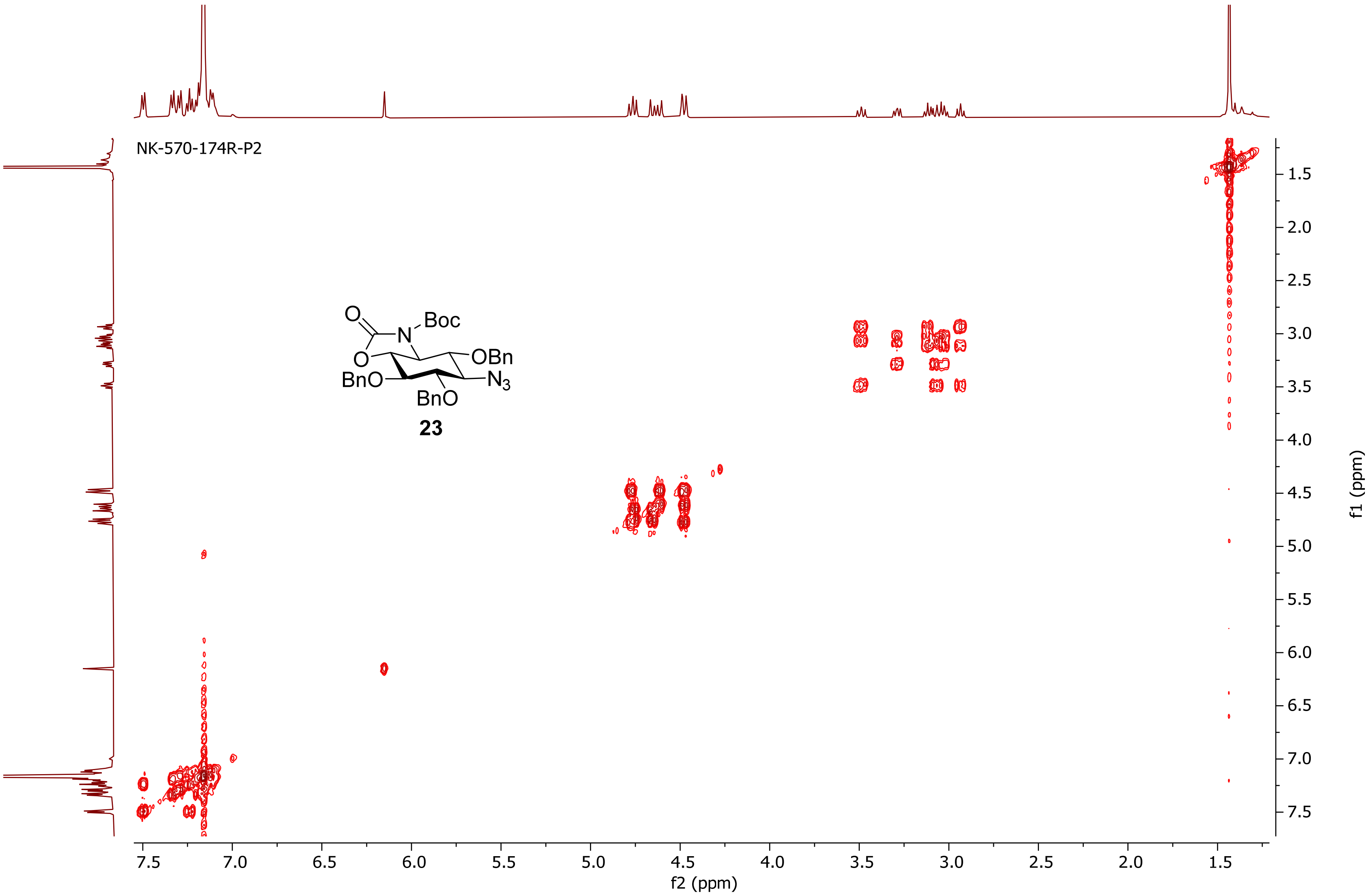

HSQC NMR Spectra (500 MHz, Benzene-d_6) of 1,3-Di(deamino)-1-azido-3-*N*-tert-butylcarboxy-2,5,6-tri-*O*-benzyl-3,4-oxazolidino-streptamine (**23**)

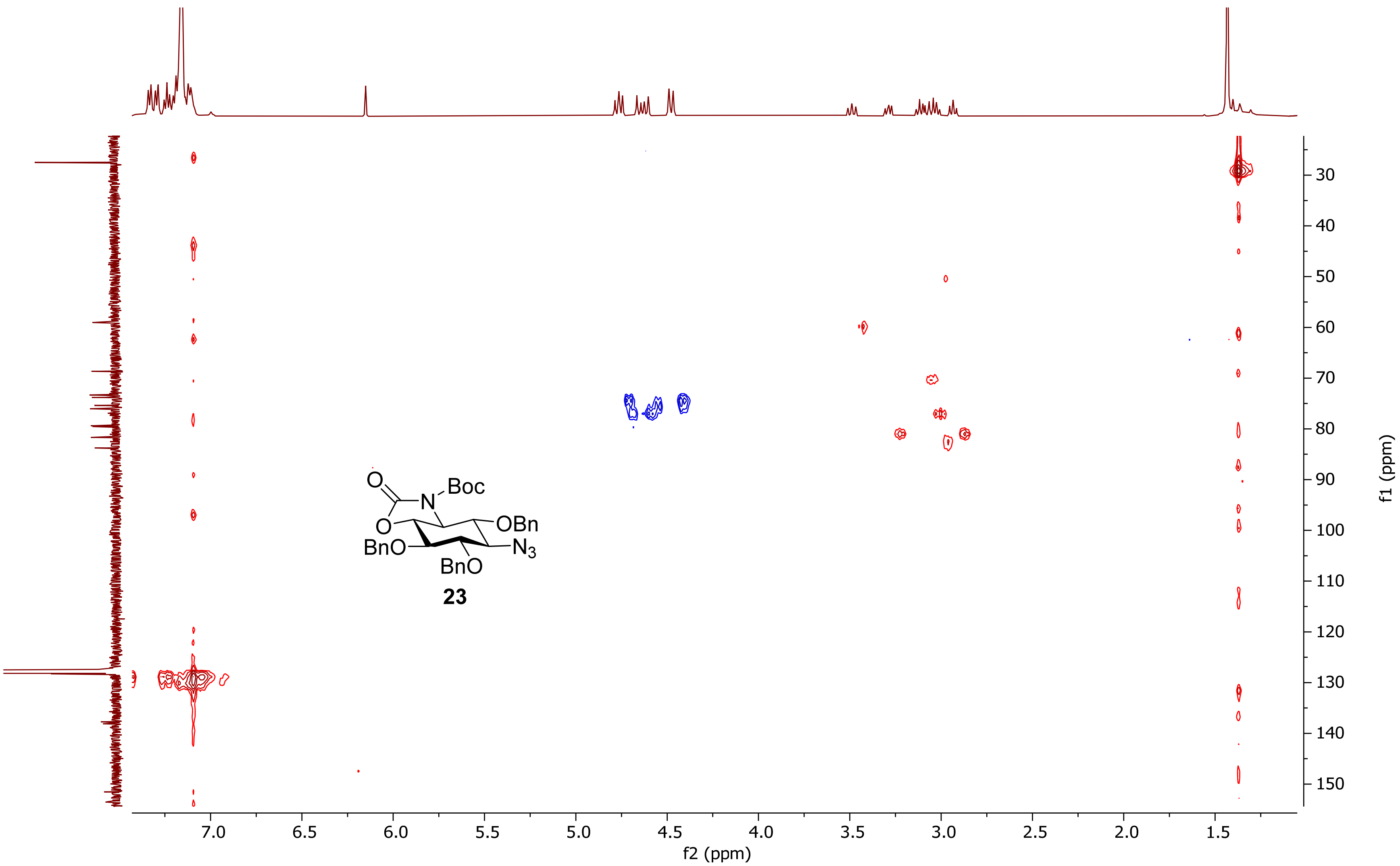

Supplement: Supplementary file 1 — jo3c02922_si_001.pdf [file jo3c02922_si_001.pdf]
